# Synthesis of furan-2-ones and spiro[furan-2,3ʹ-indoline] derivatives using polyether sulfone sulfamic acid catalysis

**DOI:** 10.1038/s41598-024-76707-0

**Published:** 2024-10-29

**Authors:** Seyed-Mola Khatami, Mehdi Khalaj, Maryam Zarandi, Taha Zeynali, Ashraf S. Shahvelayati

**Affiliations:** 1 Department of Chemical Industry, Technical and Vocational University (TVU), Tehran, Iran; 2 Department of Chemistry, Islamic Azad University, Buinzahra Branch, Buinzahra, Iran; 3 Department of Chemistry, College of Basic Sciences, Shahre Rey Branch, Yadegar-e- Imam Khomeini (RAH), Islamic Azad University, Tehran, Iran

**Keywords:** 2ʹ,5-Dioxo-5H-spiro[furan-2,3ʹ-indoline]-3-carboxylate derivatives, Furan-2-ones, Butenolides, Spiro compounds, Polyether sulfone sulfamic acid, Chemistry, Materials science

## Abstract

Polyether sulfone sulfamic acid (PES-NHSO_3_H) was prepared by simple sulfonation of a modified polyether sulfone. The number of acidic sites (SO_3_H) was determined to be 4.23 mmol H^+^/g by acid-base titration and 4.29 mmol H^+^/g by barium sulfate test. PES-NHSO_3_H was used as an efficient acidic catalytic system for the preparation of functionalized furan-2-ones and 2ʹ,5-dioxo-5 H-spiro[furan-2,3 ʹ -indoline]-3-carboxylate derivatives *via* the three-component reaction of anilines, aldehydes/1-ethylindoline-2,3-dione, and diethyl acetylene dicarboxylate in high yields (85–97%). The effect of polar and non-polar solvents on the productivity of the reaction was investigated. The catalyst was recovered 11 times without losses in catalytic potential.

**Supplementary Information:**

The online version contains supplementary material available at 10.1038/s41598-024-76707-0.

## Introduction

The synthesis of organic compounds with structural motifs similar to those found in natural products poses a significant challenge in synthetic chemistry. Natural compounds have evolved over millions of years, often resulting in highly complex structures with unique biological activities. Among these, cyclic esters, known as lactones, play a pivotal role in organic synthesis due to their prevalence in many bioactive natural products. Lactones are integral to the structure of numerous compounds with therapeutic potential, which have made them attractive targets for synthetic efforts. Their ability to serve as a core scaffold in drug design underscores their importance, as they often contribute to critical biological activities^1–4^.

A particularly noteworthy subclass of lactones is the butenolides. These compounds are cyclic γ-lactones containing a double bond, and they are significant due to their widespread biological activities, including antimicrobial, anticancer, anti-inflammatory, and antidiabetic effects. Butenolides are found in various natural products, and their core structure is an essential scaffold for many therapeutic agents^1–4^.

**Scheme 1:**
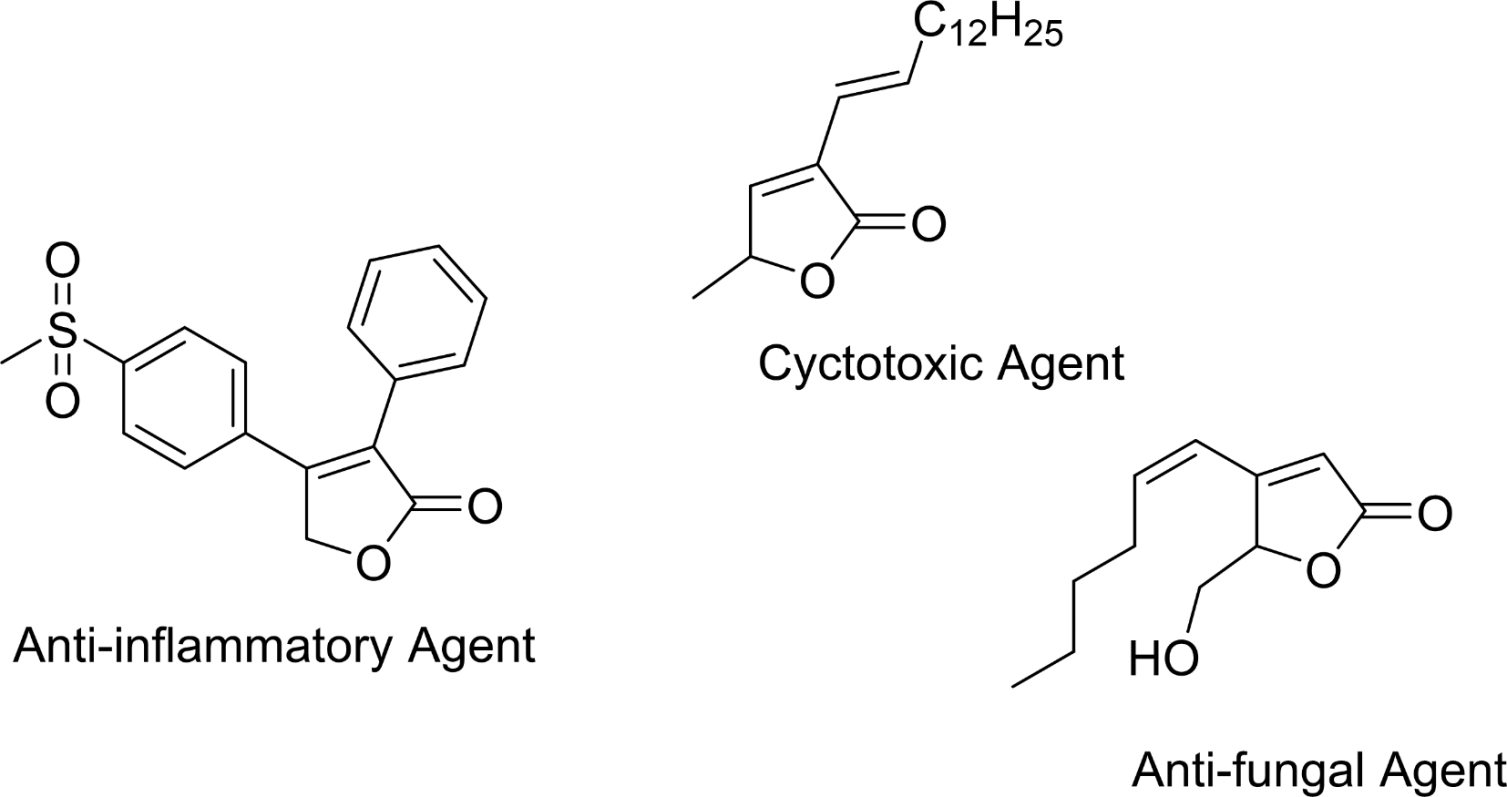
Chemical structure of some butenolides with biological properties.

For instance, butenolides have demonstrated the ability to inhibit the germination of certain plants, making them useful for controlling unwanted vegetation in agriculture^5^. Additionally, they have been shown to inhibit influenza H1N1, making them promising antiviral agents^6^. Butenolides also exhibit potential in medicine, including tyrosinase inhibition, antimicrobial activity, and even cyclooxygenase-2 (COX-2) inhibition, which is relevant to inflammation management^7–9^. Furthermore, their capacity to inhibit α-glucosidase highlights their significance in diabetes treatment, while their antiradical and antibacterial properties make them suitable candidates for a variety of therapeutic applications^9–12^ (Scheme 1).

Although butenolides can be isolated from natural sources, several synthetic strategies have been developed to prepare these compounds. Methods such as electrophilic substitution reactions, for example, using 3-bromo-2-triisopropylsilyloxyfuran^13^, and reactions involving 4-tosyl-2(5*H*)-furanone with boronic acids^14^, have been established for the synthesis of butenolides. Additionally, cyclization reactions of 3-butenoic acids^16^, and functionalization of acrylic acids with formaldehyde^15^, have also been explored. Despite their utility, these methods often require harsh conditions, long reaction times, and multi-step procedures, motivating the search for simpler, more efficient synthetic approaches.

In recent years, multi-component reactions (MCRs) have emerged as a highly effective strategy for the synthesis of butenolides. These reactions offer the advantage of combining several reactants in a single step, thus improving efficiency and minimizing waste. For instance, a notable three-component reaction involves the condensation of indole, Meldrum’s acid, and aryl glyoxal under reflux conditions in acetonitrile, catalyzed by triethylamine. This approach enables the formation of butenolides straightforwardly and efficiently^17^. Furthermore, a similar three-component reaction involving amines, dimethyl acetylenedicarboxylate, and aryl glyoxal was developed by Shahbazi-Alavi et al., using the HPA-ZSM-5 nanocatalyst, demonstrating the power of nanocatalysts in enhancing reaction efficiency^18^. More recently, a comparable method was reported by Ebrahimi et al., utilizing nano-CuO as a catalyst for the same transformation, showcasing the growing role of nanocatalysts in organic synthesis^19^.

Another promising strategy for butenolide synthesis involves the use of aniline derivatives, aldehydes, and acetylenic esters in a three-component reaction. This approach has become an efficient method for generating butenolide derivatives, with the reaction being catalyzed by a variety of Lewis and Brønsted acids. Catalysts such as β-cyclodextrin^20^, SnCl_2_^21^, ZnO nanoparticles^22^, Al(HSO_4_)_3_^23^, and tetra-n-butylammonium bisulfate^24^ have been successfully employed to promote this reaction. These catalysts not only enhance the rate of reaction but also improve the yields and selectivity of the desired butenolide products. Other catalytic systems that have been investigated include PPA/SiO_2_^25^, SnO nanoparticles^26^, HY zeolite^27^, Al-doped ZnO nanostructures^28^, nano-CdZr_4_(PO_4_)_6_^29^, and *N*-methyl-2-pyrrolidonium hydrogen sulfate^30^, all of which have proven effective in driving the reaction towards higher efficiency and greener synthetic practices. The development of these catalytic systems offers significant improvements in the practical synthesis of butenolides, contributing to the sustainability of the process.

According to the above achievements, the synthesis of butenolides remains an important area of research in both organic and medicinal chemistry. The emergence of multi-component reactions, especially those involving novel catalytic systems like nanocatalysts, has revolutionized the synthetic approach to butenolides. These advances enable the efficient and sustainable production of butenolide derivatives, which hold promise for a wide range of applications in drug discovery and agrochemicals. As research in this field continues to evolve, further improvements in reaction efficiency and environmental sustainability will likely open new doors for the application of butenolides in pharmaceuticals and beyond.

On the other hand, the modification of polymers is believed to yield heterogeneous catalysts, which are corrosion-free and stable while reducing the toxic effects of liquid catalysts. Polyether sulfone (PES) is a high-performance thermoplastic known for its remarkable mechanical, thermal, and chemical stability, which makes it ideal for use in harsh environments across diverse industrial sectors. However, PES’s hydrophobic nature limits its functionality in applications requiring high water affinity, such as membrane processes and catalytic reactions. Modification of PES through sulfonation, which introduces sulfonic acid (–SO_3_H) groups, offers a means to overcome these limitations by enhancing its hydrophilicity, ion exchange capacity, and electrical conductivity. These modifications not only improve water uptake but also enable sulfonated polyether sulfone to serve as an effective acid catalyst in various reactions, such as esterification, dehydration, and transesterification. The sulfonic acid groups enhance the material’s thermal stability, making it suitable for catalytic processes conducted under harsh conditions. Furthermore, sulfonated PES exhibits high stability and durability in both homogeneous and heterogeneous systems, demonstrating its potential as a robust and eco-friendly catalyst for green chemistry applications. The combination of its structural stability, catalytic activity, and environmental compatibility positions sulfonated PES as an attractive material for sustainable catalytic processes in a wide range of chemical transformations^31–34^.

**Scheme 2:**
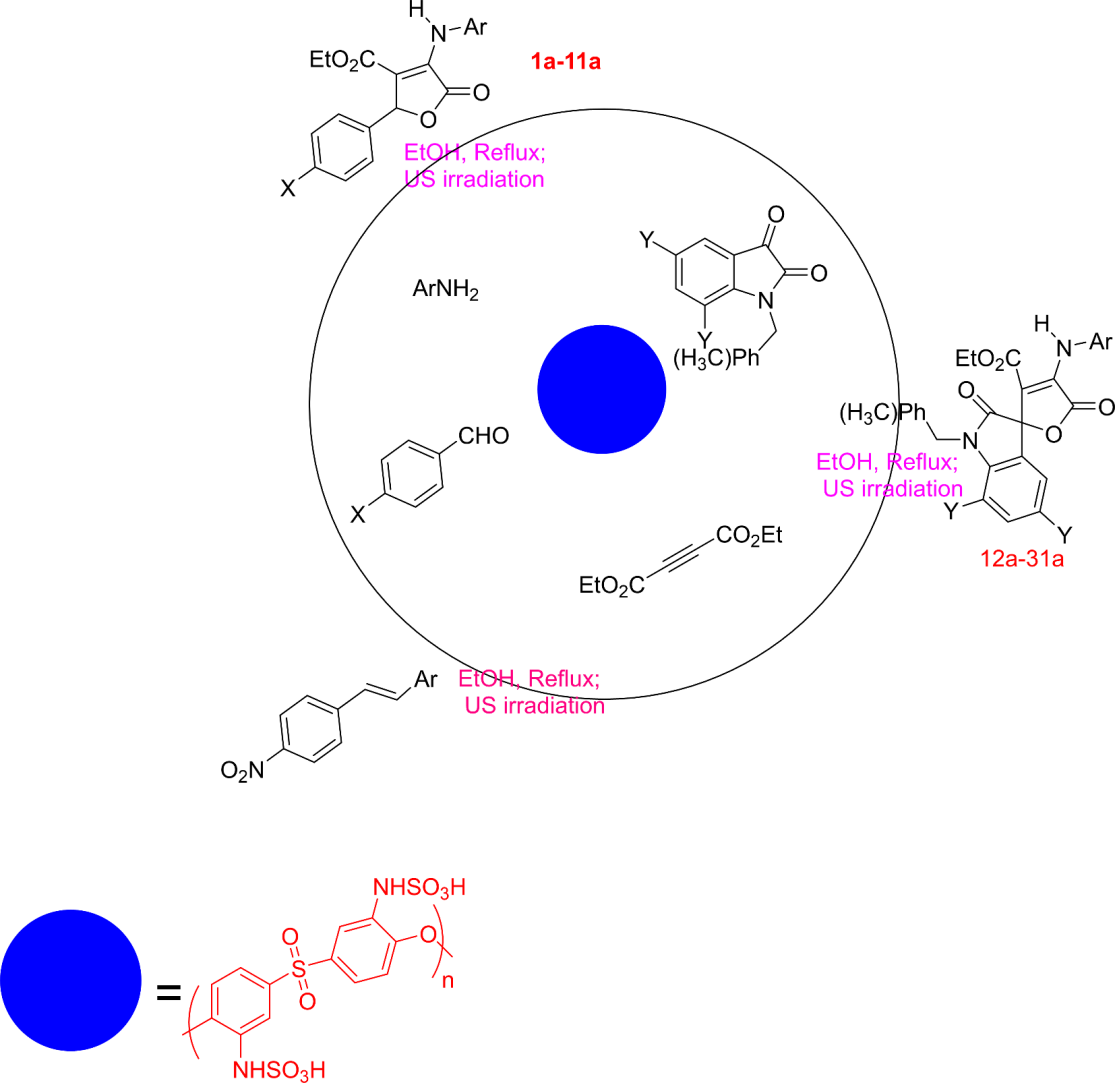
Preparation of **1a-31a** using polyether sulfone sulfamic acid (PES-NHSO_3_H).

Thus in continu of our researches^35–49^, in this work, we wish to report the PES-NHSO_3_H catalyzed synthesis of some butenolide derivatives, including highly functionalized furan-2-ones and spiro[furan-2,3ʹ-indoline] derivatives in high yields (Scheme 2).

## Experimental

### Reagents and instrumentation

All reagents were purchased from Merck and Aldrich Chemical Companies and used without further purification. A Bruker Avance DPX 500 MHz spectrometer was used to record the NMR spectra. A Heraeus CHN-O-Rapid analyzer was applied for elemental analysis. Melting points were measured on an Electrothermal IA9100 melting point apparatus and uncorrected. Thin layer chromatography (TLC) was performed on silica plates as the solid support using hexane/ethyl acetate (90/10) as the eluting solvent.

### Preparation of polyether sulfone amino sulfonic acid

#### (A) Nitration of polyether sulfone

A 500 mL flask was placed on an ice bath and filled with 60 mL of HNO_3_ and 80 mL of H_2_SO_4_. Subsequently, under stirring, 15 g of PES were slowly added to the solution. The reaction mixture was stirred overnight at 65 °C (Scheme 2).

#### (B) Preparation of amino polyether sulfone

In a 500 mL flask equipped with a condenser, 30 g of SnCl_2_ were dispersed in 100 mL of ethanol, followd by the addition of 60 mL of HCl (37%) and stirring until SnCl_2_ was completely solved. Subsequently, 15 g of nitrated polyether sulfone were added slowly into the mixture. The reaction mixture was stirred at 70 °C overnight and subsequently neutralized with NaOH. The product was separated, washed with water, and dried (Scheme S1).

#### (C) Preparation of polyether sulfone sulfamic acid

In a 500 mL flask equipped with a condenser, 10 g of amino polyether sulfone were dispersed in 100 mL of toluene, followd by the addition of ClSO_3_H under stirring. The reaction mixture was stirred at 80 °C overnight. Finally, the product was separated, washed with diethyl ether, and dried (Scheme 2).

## Elemental analysis of polyether sulfone sulfamic acid

The elemental analysis of polyether sulfone sulfamic acid was performed on a CHNS analyzor and the correponding percentages were found to be 34.87, 2.99, 6.34, and 20.78% for C, H, N, and S, repectively.

### Acidity measurement

#### (A) Acid–base titration

1 g of the as prepared catalyst was added to a solution of NaCl (50 mL, 3 M) and the resulting mixture was stirred overnight for the ion exchange between Na^+^ and H^+^ at room temperature. Next, the solid was filtered off and washed with water. The solution was titrated with a solution of sodium hydroxide (0.1 M) using phenolphthalein as the pH indicator. The acidity was determined to be 4.23 mmol H^+^/g.

#### (B) Barium sulfate test

1 g of the as prepared catalyst was dispersed in 100 mL of deionized water and mixed with a solution of H_2_O_2_ (50 mL, 30%) and NaOH (2 g). The resulting mixture was then stirred for 2 h at 50 °C for the complete oxidation of sulfonic groups to sulfate (SO_4_^2−^) ions. Next, the solution was titrated with a solution of barium chloride (1 M). The sample was aged for BaSO_4_ precipitation. The collected barium sulfate was carefully weighed and used to determine the amount of sulfate ions. Accordingly, the H^+^ capacity of the sample was determined to be 4.29 mmol H^+^/g.

### Preparation of 1a-11a

In a 100 mL flask equipped with a condenser containing EtOH (20 mL), aldehyde (1 mmol), aromatic amine (1 mmol), and diethyl acetylene dicarboxylate (1 mmol), polyether sulfone sulfamic acid (0.05 g) was added and the mixture was subjected to ultrasonic irraiation at reflux for periods indicated in Table 2. Upon completion of the reactions (monitored by TLC), the solvent was concentrated, and the crude product was washed with diethyl ether or ethanol to afford the pure product.

### Preparation of 12a-31a

In a 100 mL flask equipped with a condenser containing EtOH (20 mL), 1-ethylindoline-2,3-dione (1 mmol), aromatic amine (1 mmol), and diethyl acetylene dicarboxylate (1 mmol), polyether sulfone sulfamic acid (0.05 g) was added and the mixture was subjected to ultrasonic irraiation at reflux for periods indicated in Tables 2 and 3. Upon completion of the reactions (monitored by TLC), the solvent was concentrated, and the crude product was washed with diethyl ether or ethanol to afford the pure product.

## Results and discussion

**Scheme 3:**
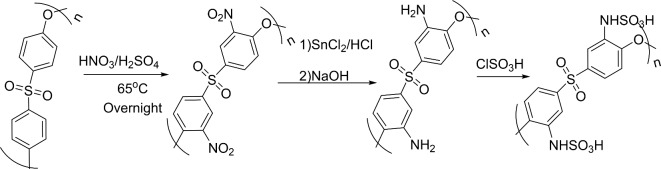
Preparation of polyether sulfone sulfamic acid (PES-NHSO_3_H).

Scheme 3 shows the preparation of PES-NHSO_3_H. Accordingly, for the preparation of polyether sulfone sulfamic acid, polyether sulfone was first modified to nitro polyether sulfone through the nitration reaction using concentrated nitric acid. Nitro groups were then reduced to amino groups to form amino polyether sulfone, which could be easily sulfonated by ClSO_3_H to form PES-NHSO_3_H.

**Fig. 1:**
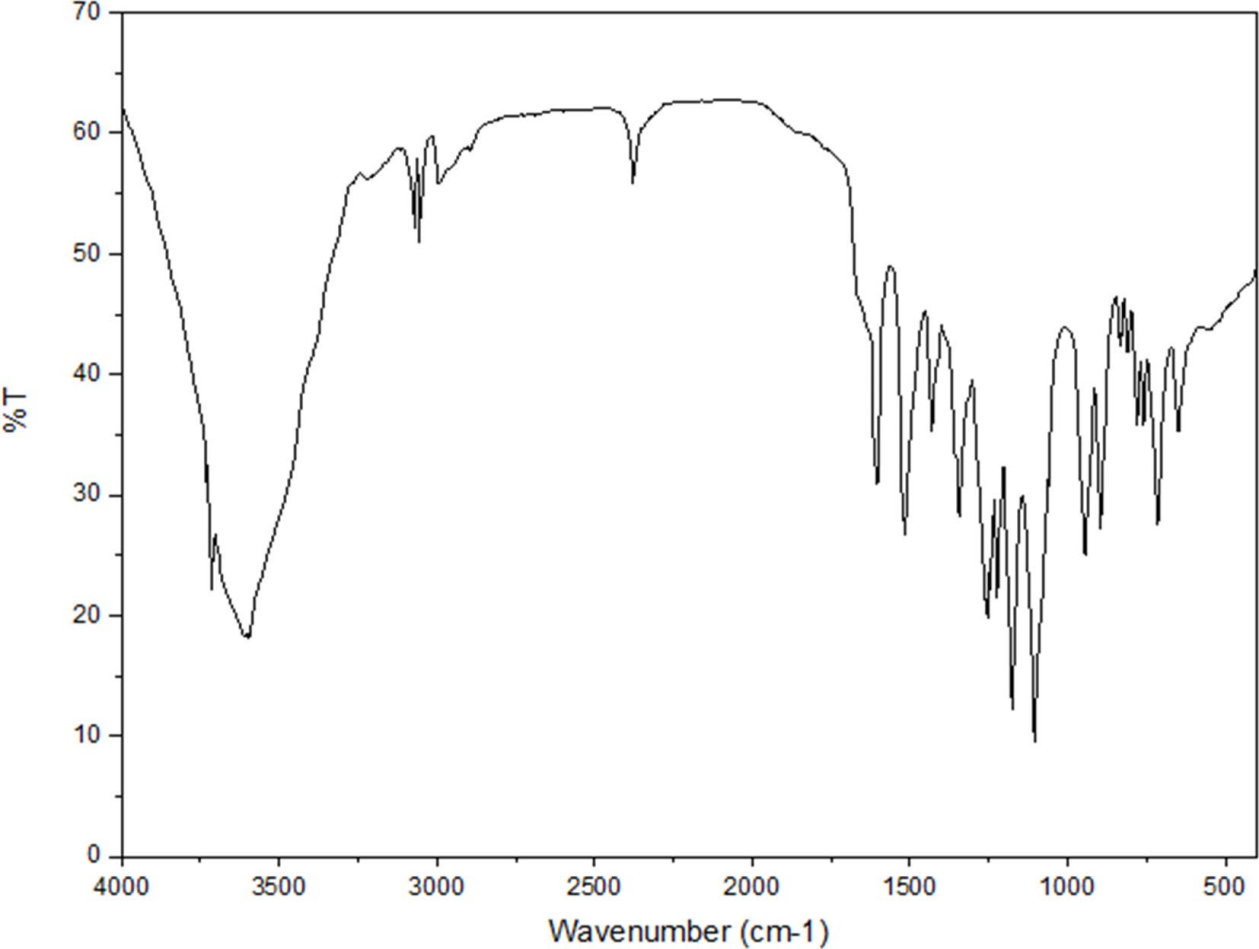
FT-IR spectrum of PES-NHSO_3_H.

Figure 1 shows the FT-IR spectrum of PES-NHSO_3_H. The sample has adsorption bonds at 3713, 3069, 1607, 1517, 1342, 1255, 1172, 1104, 941, and 892 cm^−1^ related to the stretching and bending vibrations of N–H, C–H, C = C, C–O, S = O, and C–N bonds, respectively. The broad bond at 3100–3700 cm^−1^ corresponds to the SO_3_H group vibration.

At first, the preparation of (**1a**) from the reaction of aniline (1 mmol), benzaldehyde (1 mmol), and diethylacetylenedicarboxylate (1 mmol) was investigated under ultrasonic (US) irradiation using PES-NHSO_3_H as the catalyst. In the absence of a catalyst, stirring the reaction mixture at r.t. for 24 h afforded no products. Similarly, when the reaction mixture was refluxed in non-polar and low-polar solvents such as *n*-hexane, *n*-heptane, decane, benzene, toluene, diethyl ether, and dichloromethane, the substrates remained unreacted even after prolonged time (24 h). The reaction gave poor product yields under solvent-free conditions. Similarly, when polar solvents were replaced by non-protic solvents, the product was formed in low yields. Notably, polar protic solvents were found to be more suitable for the reaction in terms of reaction times and yields. Finally, among the solvents screened, EtOH was found more suitable as it formed higher yields of the product (Table 1). In continuation of these investigations, it was found that the optimal dosage of the catalyst (polyether sulfone sulfamic acid) was 0.05 g.

**Table 1:** Optimization of the reaction conditions under US irradiation (preparation of **1a**).


Media	Temperature (°C)	Time (h)	Catalyst (g)	Solvent (Yield (%))
EtOH	25	24	0.05	–
Solvent-free	25	24	0.05	–
Solvent-free	50	5	0.05	–
Solvent-free	80	3	0.05	15
Solvent-free	100	3	0.05	26
Non-polar and low-polar	Reflux	24	0.05	*n*-hexane, *n*-heptane, decane, benzene, toluene, diethyl ether, and dichloromethane
Polar non-protic	Reflux	24	0.05	EtOAc (14), DMF (36), CH_3_CN (18)
Polar protic: MeOH	Reflux	5	0.05	69
EtOH	Reflux	4	0.05	95
H_2_O	Reflux	7	0.05	36
EtOH	Reflux	8	0.01	15
EtOH	Reflux	5	0.025	74
EtOH	Reflux	4	0.075	94

Next, using the optimized conditions, the catalytic potential of polyether sulfone sulfamic acid in the preparation of furan-2(5*H*)-ones was studied using different amines and aldehydes (Table 2). The pure products were isolated in high yields (Table 2, products **1a-9a**,** 11a**). No product was formed when aromatic aldehydes bearing electron-withdrawing groups were used and only an imine product (**10a**) was isolated.

**Table 2:**
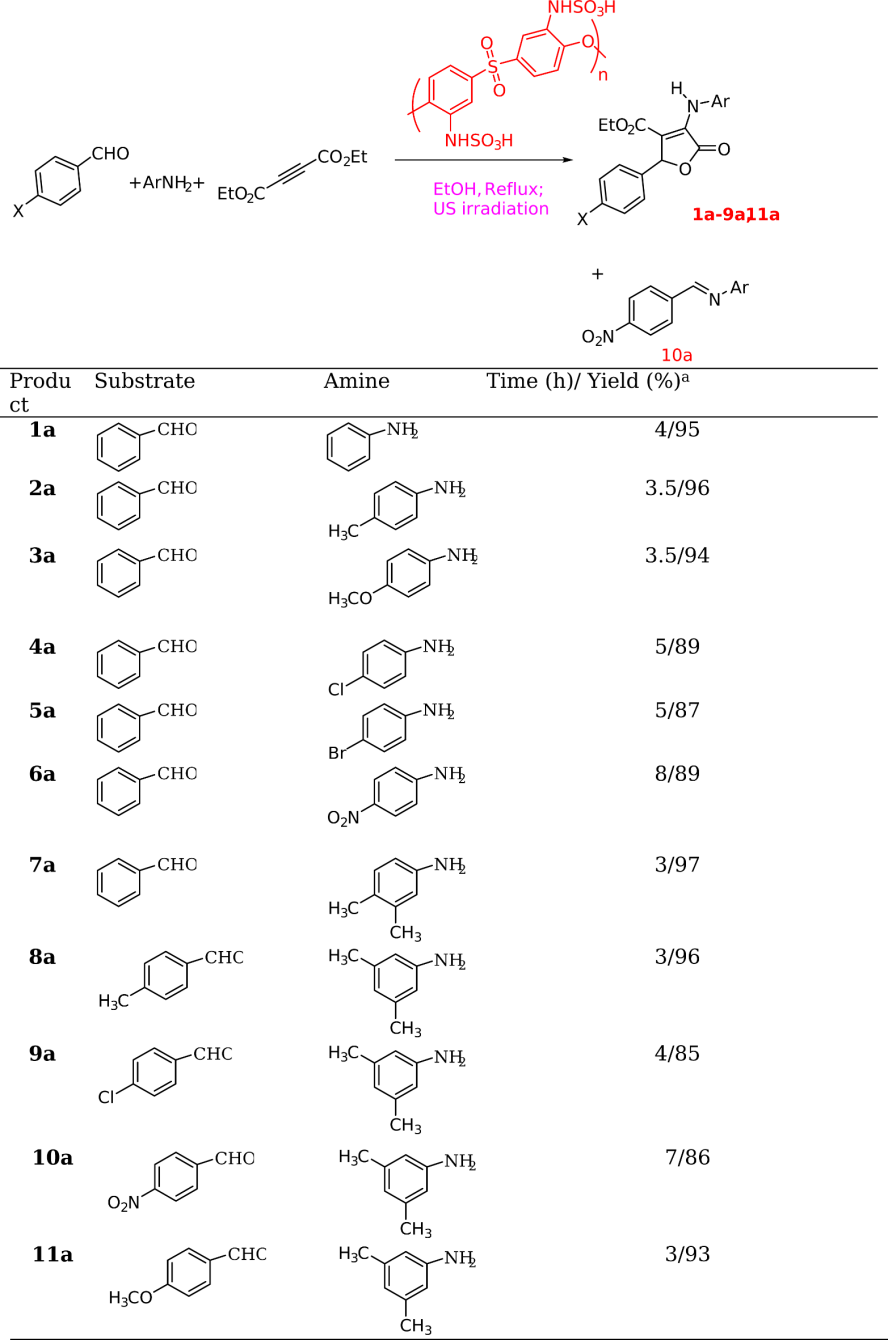
Synthesis of **1a-11a**.

Next, diverse aldehydes were replaced by various indoline-2,3-dione derivatives under optimized conditions, as depicted in Table 1. The results are shown in Table 3. As shown, 2’,5-dioxo-5*H*-spiro[furan-2,3’-indoline]-3-carboxylate derivatives were prepared in high to excellent yields. Electron-withdrawing groups such as NO_2_ and Cl were found to hurt the reaction rate.

**Scheme 4:**
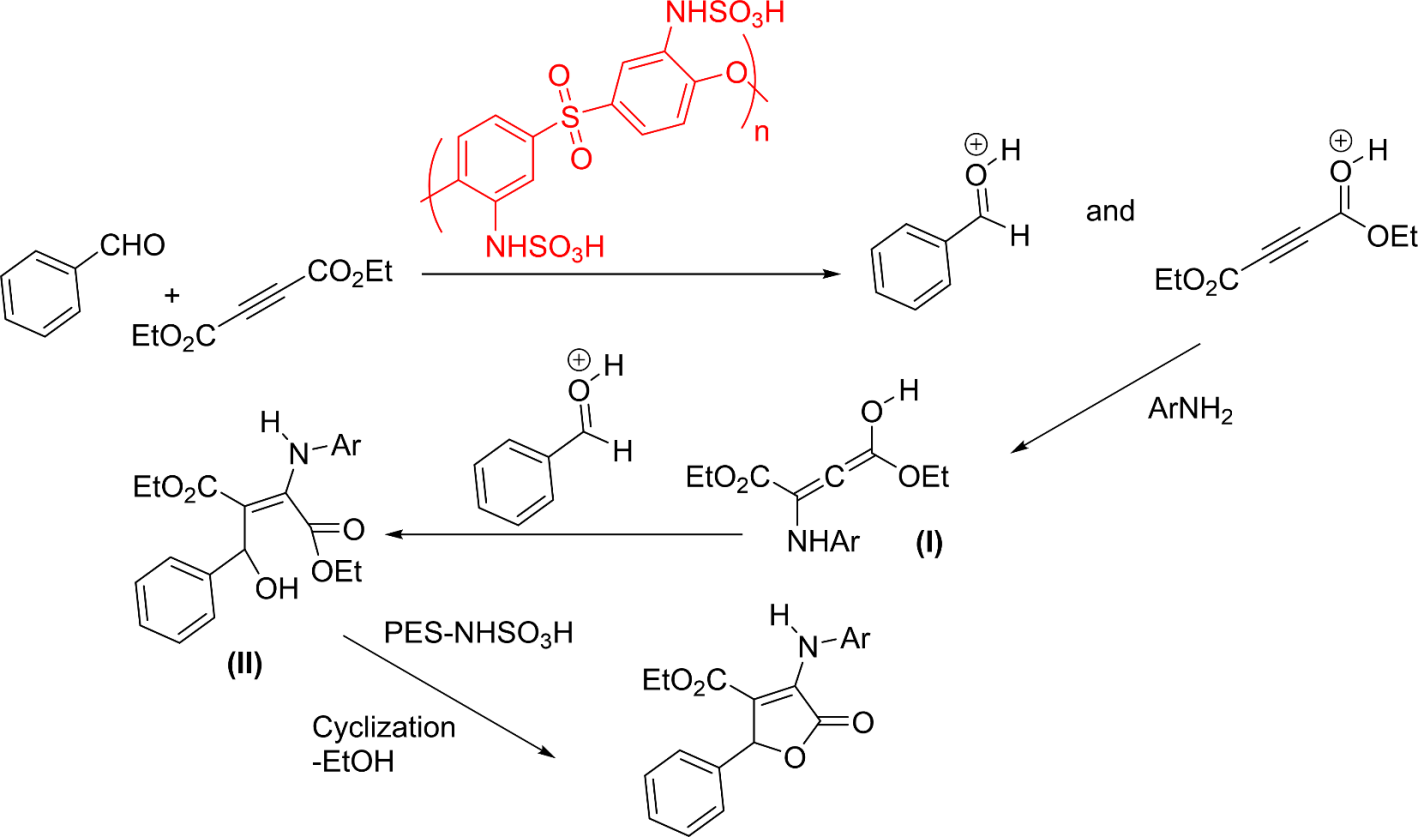
Plausible mechanism for the preparation of target compounds **(1a-31a)**.

The plausible route for preparing final products is shown in Scheme 4. The progress of the reaction starts with the protonation of oxygen atoms in the aldehyde and diethylacetylenedicarboxylate (DMAD). These protonated compounds are more stable in protic solvents thus ethanol is a suitable media to increase the reaction rate. Next, the amine nucleophile attaches to protonated DMAD to form intermediate **(I)**. Afterwards, the reaction proceeds with the reaction between intermediate **(I)** with protonated aldehyde to form intermediate **(II)**. It seems that this step controls the reaction rate. Amines-bearing electron-withdrawing groups are weaker nucleophiles than those of electron-donating groups. Thus, the reaction times are longer when amines with electron-withdrawing groups are used.

**Table 3:**
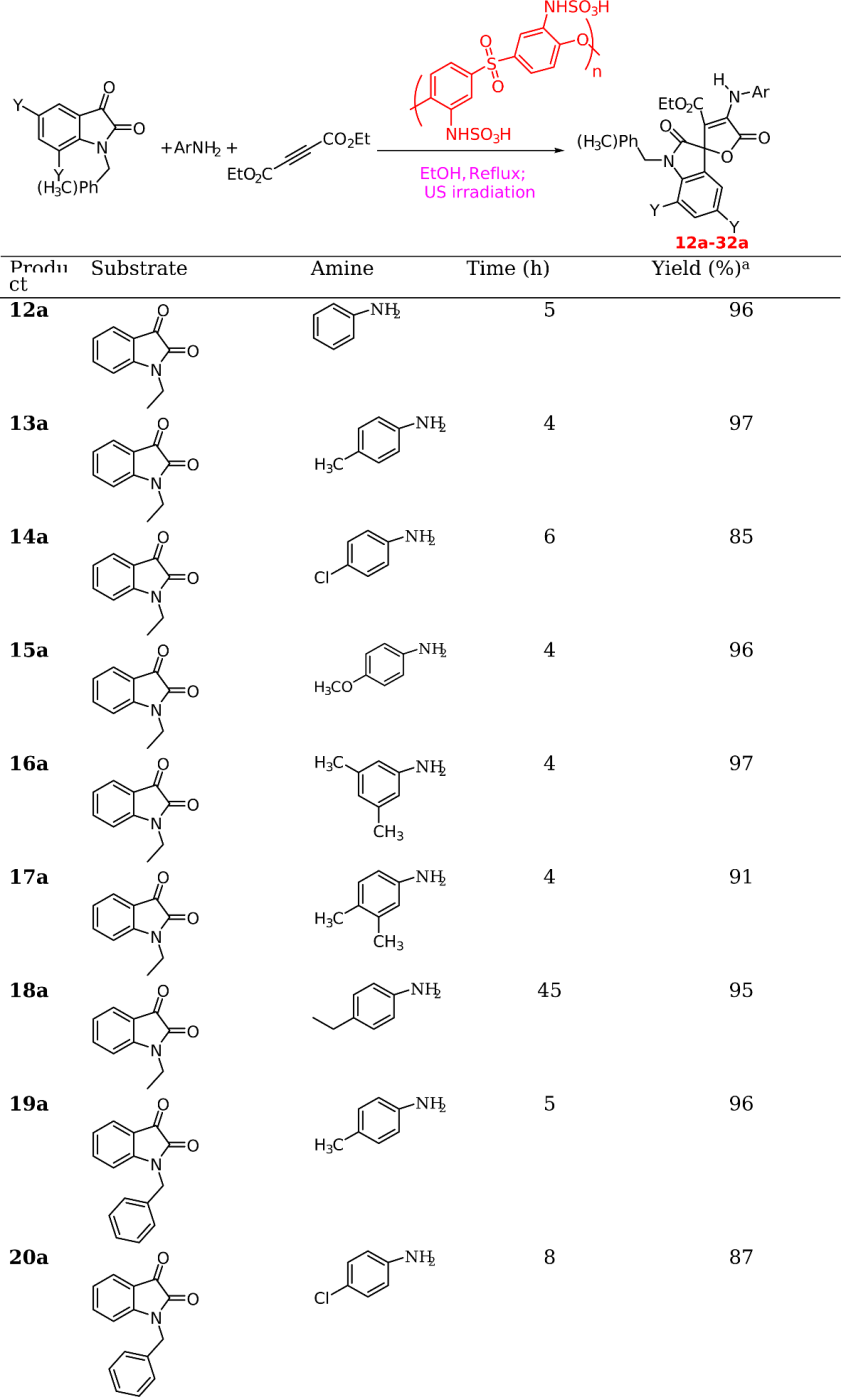

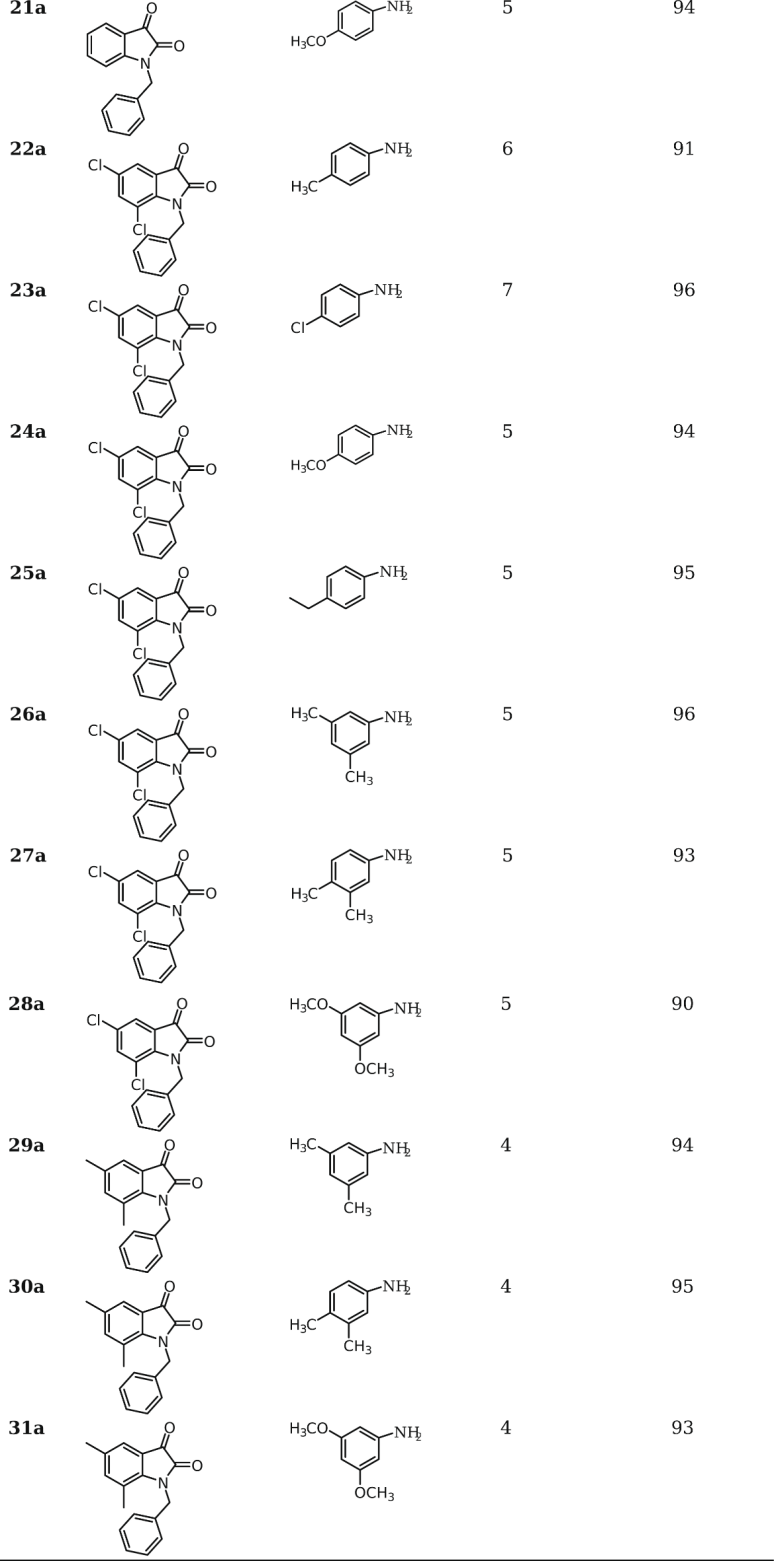
Synthesis of **12a-31a**.

Finally, 2ʹ,5-dioxo-5*H*-spiro[furan-2,3ʹ -indoline]-3-carboxylate derivatives were prepared in good yields. Simple filtration and washing yielded pure products. In addition, this protocol uses a heterogeneous solid acid making it superior to the reactions involving hazardous liquid acidic catalysts.

**Fig. 2:**
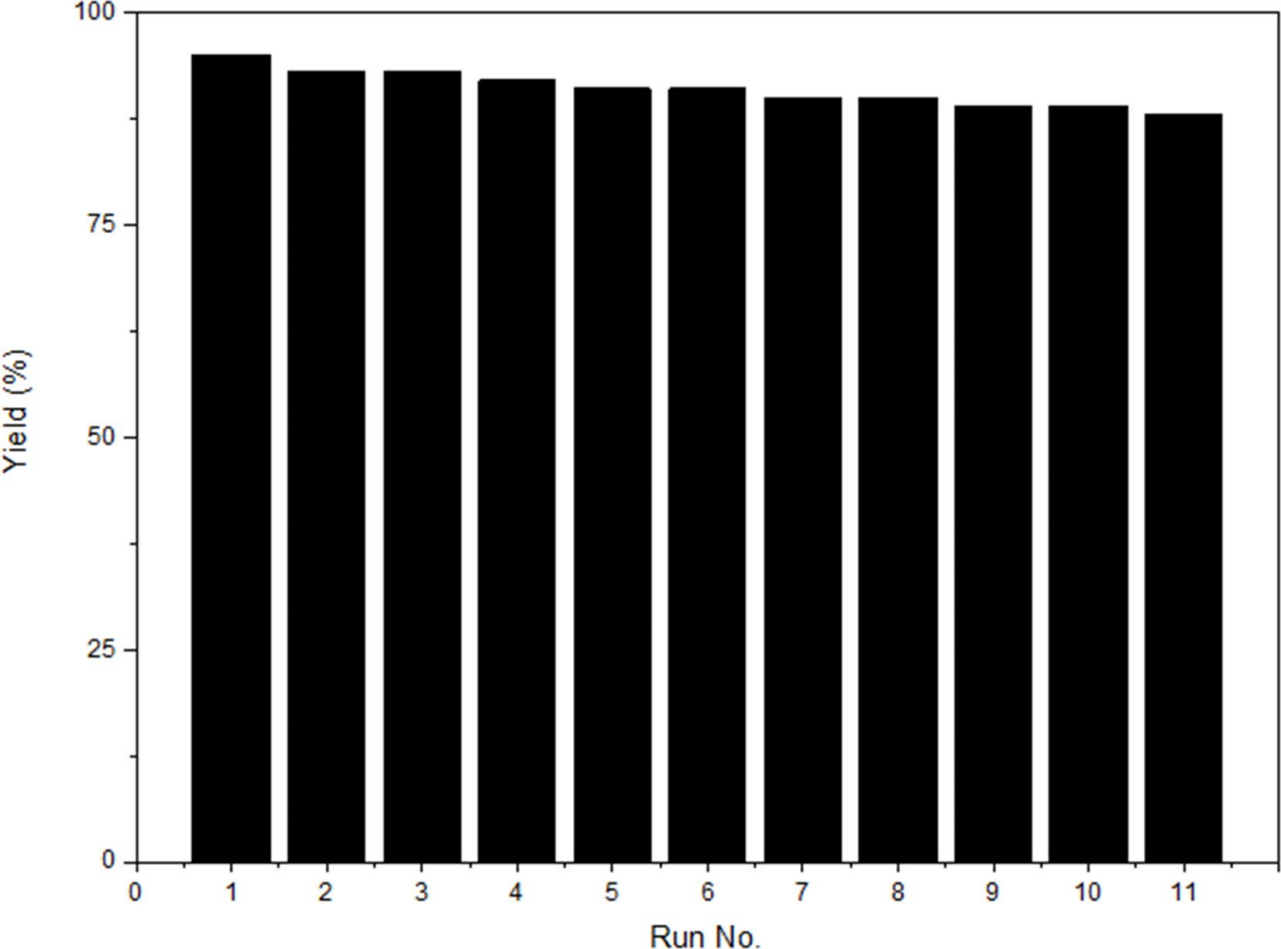
Recovery tests results for PES-NHSO_3_H.

The catalyst recovery tests were performed to investigate the possible stability and reusability of PES-NHSO_3_H. The results are depicted in Fig. 2. Accordingly, the catalyst is stable during the reaction and could be reused successfully for 11 times.

## Conclusion

A simple synthetic method for the preparation of furan-2(5*H*)-ones and 2’,5-dioxo-5*H*-spiro[furan-2,3’-indoline]-3-carboxylate derivatives has been developed, which provides good product yields in short reaction times using polyether sulfone sulfamic acid catalyst. Furthermore, the possibility of a non-toxic catalyst, cheapness, and no need for chromatographic separation of the products, are among the environmental benefits of this process.

## Electronic supplementary material

Below is the link to the electronic supplementary material.

Supplementary Material 1

## Data Availability

The datasets used and/or analysed during the current study available from the corresponding author on reasonable request.
