# Supplementary material for: Synthesis of furan-2-ones and spiro[furan-2,3ʹ-indoline] derivatives using polyether sulfone sulfamic acid catalysis
Source: Sci Rep. 2024 Oct 29;14:26008. doi: 10.1038/s41598-024-76707-0 (PMC11522323; doi:10.1038/s41598-024-76707-0)
Supplement: Supplementary file 1 — Supplementary Material 1 [file 41598_2024_76707_MOESM1_ESM.docx]

**Ultrasonic-assisted multi-component synthesis of highly functionalized furan-2-ones and spiro[furan-2,3'-indoline] derivatives using polyether sulfone sulfamic acid catalysis**

Seyed-Mola Khatami*^a^, Mehdi Khalaj^^[[1]](#footnote-1)^*b^, Maryam Zarandi^b^, Taha Zeynali^b^ and Ashraf S. Shahvelayati^c^

^a^Department of Chemical Industry, Technical and Vocational University (TVU), Tehran, Iran

^b^Department of Chemistry, Islamic Azad University, Buinzahra Branch, Buinzahra, Iran

^c^Department of Chemistry, College of Basic Sciences, Yadegar-e- Imam Khomeini (RAH) Shahre Rey Branch, Islamic Azad University, Tehran, Iran

**Experimental**

**Reagents and Instrumentation:**

All reagents were purchased from Merck and Aldrich and used without further purification. Instruments used for characterization are Bruker Avance DPX 500 MHz instrument for NMR spectra, and Heraeus CHN-O-Rapid analyzer for elemental analysis. Melting points were taken on an Electrothermal IA9100 melting point apparatus and are uncorrected. Thin layer chromatography (TLC) was done on silica plates as the solid support using hexane/ethyl acetate (90/10) as the eluting solvent.

**Preparation of polyether sulfone amino sulfonic acid**

*a) Nitration of polyether sulfone*

A 500 mL balloon was sunk into an ice bath and filled with 60 mL HNO_3_ and 80 mL H_2_SO_4_. Subsequently under stirring 15 g of PES was slowly added to the solution. The reaction was stirred overnight at 65°C to be completed (Scheme S_1_).

*b) Preparation of amino polyether sulfone*

In a 500 mL balloon equipped with a condenser, 30 g SnCl_2_ was dispersed in 100 mL of ethanol, and 60 mL of HCl (37%) was added to the mixture and stirred until SnCl_2_ was completely solved. Subsequently, 15 g of nitrated polyether sulfone was added slowly. The reaction was stirred at 70°C overnight and subsequently neutralized with NaOH. The mass was separated, washed with water, and died (Scheme S_1_).

*c) Preparation of polyether sulfone sulfamic acid*

In a 500 mL balloon equipped with a condenser, 10 g of amino polyether sulfone was dispersed in 100 mL of toluene and subsequently ClSO_3_H was added to the reaction under stirring. The reaction was stirred at 80°C overnight. Finally, the mass was separated, washed with diethyl ether, and died (Scheme S_1_).

**Elemental Analysis of Polyether Sulfone Sulfamic Acid**

The elemental Analysis of polyether sulfone sulfamic acid was performed on a CHNS analyzer and their values were found to be: C, 34.87; H, 2.99; N, 6.34; S, 20.78.

**Acidity measurement:**

**a) Acid-base titration**

1 g of the as-prepared catalyst was added to a solution of NaCl (50mL, 3M) and the resulting mixture was stirred overnight for the ion exchange between Na^+^ and H^+^ at room temperature. Next, the solid was filtered off and washed with water. The solution was titrated with a solution of sodium hydroxide (0.1 M). Phenolphthalein was used as a pH indicator. The amount of acidity was determined to be 4.23 mmol H^+^/g.

**b) Barium sulfate test**

1 g of the as-prepared catalyst was dispersed in 100 mL of deionized water and combined with a solution of H_2_O_2_ (50mL, 30%) and NaOH (2g) and the resulting mixture was stirred for 2h at 50 °C to be completely oxidation of sulfonic groups to sulfate (SO_4_^2-^) ions. Next, the solution was titrated with barium chloride (1 M). The sample was aged for BaSO_4_ precipitation. The collected barium sulfate was carefully weighed and used to determine the amount of sulfate ions. Accordingly, the H^+^ capacity of the sample was determined to be 4.29 mmol H^+^/g.

**Scheme S_1_:** Preparation of polyether sulfone sulfamic acid

**Procedures and Spectroscopic Data**

**Preparation of 1a-11a**

In a 100 mL balloon coupled with a condenser containing EtOH (20mL), aldehyde (1 mmol), aromatic amine (1 mmol), diethyl acetylene dicarboxylate (1 mmol), polyether sulfone sulfamic acid (0.05g) was added and the mixture was irradiated in the water bath of the ultrasonic cleaner at reflux condition for a period as indicated in Tables 2. As the reactions were completed (monitored by TLC), the solvent was concentrated, and the crude product was washed with diethyl ether or ethanol to afford the pure product.

Preparation of **12a-32a**

In a 100 mL balloon coupled with a condenser containing EtOH (20 mL), 1-ethylindoline-2,3-dione (1 mmol), aromatic amine (1 mmol), diethyl acetylene dicarboxylate (1 mmol), polyether sulfone sulfamic acid (0.05g) was added and the mixture was irradiated in the water bath of the ultrasonic cleaner at reflux condition for a period as indicated in Tables 2,3. As the reactions were completed (monitored by TLC), the solvent was concentrated, and the crude product was washed with diethyl ether or ethanol to afford the pure product.

Ethyl 5-oxo-2-phenyl-4-(phenylamino)-2,5-dihydrofuran-3-carboxylate **(Table 2, 1a):** Solid powder, m.p.: 171-173°C; ^1^H-NMR (500 MHz, DMSO-d_6_): δ = 8.26 (s, 1H, NH), 7.39 (d, *J* = 7.8 Hz, 2H), 7.33 (t, *J* = 7.8 Hz, 1H), 7.26 (t, *J* = 7.8 Hz, 2H), 7.21 (t, *J* = 7.8 Hz, 2H), 7.07 (t, *J* = 7.8 Hz, 1H), 6.98 (d, *J* = 7.8 Hz, 2H), 5.77 (s, 1H), 4.10 (q, *J* = 6.8 Hz, 2H), 1.28 (t, *J* = 6.8 Hz, 3H) ppm; ^13^C-NMR (125 MHz, DMSO-d_6_): δ = 14.7, 31.8, 62.3, 63.1, 114.7, 123.6, 126.4, 127.8, 128.7, 129.4, 130.1, 137.2, 151.8, 164.3, 166.9 ppm; Found: C, 70.48; H, 5.36; N, 4.41% C_19_H_17_NO_4_; requires: C, 70.58; H, 5.30; N, 4.33%.

Ethyl 5-oxo-2-phenyl-4-(*p*-tolylamino)-2,5-dihydrofuran-3-carboxylate **(Table 2, 2a):** Solid powder, m.p.: 197-199°C; ^1^H-NMR (500 MHz, DMSO-d_6_): δ = 8.41 (s, 1H, NH), 7.39 (d, *J* = 7.8 Hz, 2H), 7.28-7.34 (m, 3H), 7.22 (d, *J* = 7.8 Hz, 2H), 7.06 (d, *J* = 7.8 Hz, 2H), 5.66 (s, 1H), 4.08-4.13 (q, *J* = 6.8 Hz, 2H), 2.27 (s, 3H, CH_3_), 1.27 (t, *J* = 6.8 Hz, 3H) ppm; ^13^C-NMR (125 MHz, DMSO-d_6_): δ = 14.6, 20.7, 31.6, 62.4, 63.8, 115.2, 119.1, 123.2, 127.8, 129.3, 130.2, 136.6, 137.5, 151.6, 164.1, 166.4 ppm; Found: C, 71.11; H, 5.53; N, 4.48% C_20_H_19_NO_4_; requires: C, 71.20; H, 5.68; N, 4.15%.

Ethyl 4-((4-methoxyphenyl)amino)-5-oxo-2-phenyl-2,5-dihydrofuran-3-carboxylate **(Table 2, 3a):** Solid powder, m.p.: 207-209°C; ^1^H-NMR (500 MHz, DMSO-d_6_): δ = 8.56 (s, 1H, NH), 7.39 (d, *J* = 7.8 Hz, 2H), 7.27-7.34 (m, 3H), 7.12 (d, *J* = 7.8 Hz, 2H), 6.97 (d, *J* = 7.8 Hz, 2H), 5.63 (s, 1H), 4.08-4.13 (q, *J* = 6.8 Hz, 2H), 3.72 (s, 3H, OCH_3_), 1.27 (t, *J* = 6.8 Hz, 3H) ppm; ^13^C-NMR (125 MHz, DMSO-d_6_): δ = 14.6, 31.2, 55.7, 62.1, 63.4, 110.7, 115.4, 123.6, 127.5, 129.6, 130.4, 135.3, 151.4, 155.6, 164.3, 166.1 ppm; Found: C, 67.89; H, 5.34; N, 3.92% C_20_H_19_NO_5_; requires: C, 67.98; H, 5.42; N, 3.96%.

Ethyl 4-((4-chlorophenyl)amino)-5-oxo-2-phenyl-2,5-dihydrofuran-3-carboxylate **(Table 2, 4a):** Solid powder, m.p.: 218-220°C; ^1^H-NMR (500 MHz, DMSO-d_6_): δ = 8.97 (s, 1H, NH), 7.23-7.42 (m, 9H), 5.77 (s, 1H), 4.10-4.15 (q, *J* = 6.7 Hz, 2H), 1.28 (t, *J* = 6.7 Hz, 3H) ppm; ^13^C-NMR (125 MHz, DMSO-d_6_): δ = 14.9, 31.6, 62.5, 63.7, 118.9, 123.7, 127.7, 128.9, 129.6, 130.5, 138.8, 144.1, 151.5, 164.1, 166.7 ppm; Found: C, 63.69; H, 4.45; N, 3.88% C_19_H_16_ClNO_4_; requires: C, 63.78; H, 4.51; N, 3.91%.

Ethyl 4-((4-bromophenyl)amino)-5-oxo-2-phenyl-2,5-dihydrofuran-3-carboxylate **(Table 2, 5a):** Solid powder, m.p.: 226-228°C; ^1^H-NMR (500 MHz, DMSO-d_6_): δ = 9.02 (s, 1H, NH), 7.64 (d, *J* = 7.8 Hz, 2H), 7.39 (d, *J* = 7.8 Hz, 2H),7.25-7.34 (m, 5H), 5.77 (s, 1H), 4.10-4.14 (q, *J* = 6.8 Hz, 2H), 1.28 (t, *J* = 6.8 Hz, 3H) ppm; ^13^C-NMR (125 MHz, DMSO-d_6_): δ = 14.9, 31.7, 62.8, 63.7, 119.7, 123.8, 127.6, 129.5, 129.9, 130.8, 139.8, 146.7, 151.7, 164.1, 167.1 ppm; Found: C, 56.78; H, 4.09; N, 3.44% C_19_H_16_BrNO_4_; requires: C, 56.73; H, 4.01; N, 3.48%.

Ethyl 4-((4-nitrophenyl)amino)-5-oxo-2-phenyl-2,5-dihydrofuran-3-carboxylate **(Table 2, 6a):** Solid powder, m.p.: 224-226°C; ^1^H-NMR (500 MHz, DMSO-d_6_): δ = 9.04 (s, 1H, NH), 8.07 (d, *J* = 7.8 Hz, 2H), 7.76 (d, *J* = 7.8 Hz, 2H), 7.39 (d, *J* = 7.8 Hz, 2H), 7.28-7.34 (m, 3H), 5.77 (s, 1H), 4.09-4.13 (q, *J* = 6.7 Hz, 2H), 1.28 (t, *J* = 6.8 Hz, 3H) ppm; ^13^C-NMR (125 MHz, DMSO-d_6_): δ = 15.3, 32.3, 62.9, 64.2, 123.7, 126.9, 127.8, 129.4, 130.6, 131.4, 139.7, 140.4, 151.7, 164.7, 167.6 ppm; Found: C, 62.07; H, 4.44; N, 7.55% C_19_H_16_N_2_O_6_; requires: C, 61.96; H, 4.38; N, 7.61%.

Ethyl 4-((3,4-dimethylphenyl)amino)-5-oxo-2-phenyl-2,5-dihydrofuran-3-carboxylate **(Table 2, 7a):** Solid powder, m.p.: 203-205°C; ^1^H-NMR (500 MHz, DMSO-d_6_): δ = 8.14 (s, 1H, NH), 6.27-7.35 (m, 5H), 6.89-6.92 (m, 2H), 6.66 (s, 1H), 5.78 (s, 1H), 4.05-4.09 (q, *J* = 6.8 Hz, 2H), 2.37 (s, 3H), 2.17 (s, 3H), 1.28 (t, *J* = 6.8 Hz, 3H) ppm; ^13^C-NMR (125 MHz, DMSO-d_6_): δ = 14.6, 19.8, 20.5, 31.7, 62.1, 63.6, 113.2, 114.6, 118.4, 122.8, 128.7, 129.4, 130.1, 136.7, 137.2, 137.6, 148.8, 162.7, 167.2 ppm; Found: C, 71.85; H, 6.14; N, 3.96% C_21_H_21_NO_4_; requires: C, 71.78; H, 6.02; N, 3.99%.

Ethyl 4-((3,5-dimethylphenyl)amino)-5-oxo-2-(*p*-tolyl)-2,5-dihydrofuran-3-carboxylate **(Table 2, 8a):** Solid powder, m.p.: 224-226°C; ^1^H-NMR (500 MHz, DMSO-d_6_): δ = 8.66 (s, 1H, NH), 7.13 (d, *J* = 7.6 Hz, 2H), 7.08 (d, *J* = 7.6 Hz, 2H), 6.78 (s, 1H), 6.65 (s, 2H), 5.75 (s, 1H), 4.07-4.11 (q, *J* = 6.8 Hz, 2H), 2.27 (s, 3H), 2.41 (s, 6H), 1.29 (t, *J* = 6.8 Hz, 3H) ppm; ^13^C-NMR (125 MHz, DMSO-d_6_): δ = 14.6, 20.9, 21.7, 31.8, 62.3, 63.5, 113.4, 114.5, 115.4, 126.2, 128.9, 136.8, 137.4, 137.6, 149.2, 162.2, 167.0 ppm; Found: C, 72.38; H, 6.44; N, 3.96% C_22_H_23_NO_4_; requires: C, 72.31; H, 6.34; N, 3.83%.

Ethyl 2-(4-chlorophenyl)-4-((3,5-dimethylphenyl)amino)-5-oxo-2,5-dihydrofuran-3-carboxylate **(Table 2, 9a):** Solid powder, m.p.: 233-235°C; ^1^H-NMR (500 MHz, DMSO-d_6_): δ = 8.93 (s, 1H, NH), 7.44 (d, *J* = 7.6 Hz, 2H), 7.29 (d, *J* = 7.6 Hz, 2H), 6.81 (s, 1H), 6.66 (s, 2H), 5.83 (s, 1H), 4.07-4.12 (q, *J* = 6.9 Hz, 2H), 2.43 (s, 6H), 1.29 (t, *J* = 6.9 Hz, 3H) ppm; ^13^C-NMR (125 MHz, DMSO-d_6_): δ = 14.7, 20.7, 32.1, 62.4, 63.9, 113.7, 114.6, 115.5, 129.2, 130.9, 136.8, 137.4, 143.1, 152.5, 162.7, 167.4 ppm; Found: C, 65.49; H, 5.27; N, 3.55% C_21_H_20_ClNO_4_; requires: C, 65.37; H, 5.22; N, 3.63%.

(*E*)-N-(3,5-dimethylphenyl)-1-(4-nitrophenyl)methanimine **(Table 2, 10a):** Solid powder, m.p.: 115-117°C; ^1^H-NMR (500 MHz, DMSO-d_6_): δ = 9.97 (s, 1H, CH=N), 8.27 (d, *J* = 7.8 Hz, 2H), 7.89 (d, *J* = 7.8 Hz, 2H), 7.16 (s, 2H), 6.87 (s, 1H), 2.63 (s, 6H) ppm; Found: C, 70.96; H, 5.66; N, 11.11% C_15_H_14_N_2_O_2_; requires: C, 70.85; H, 5.55; N, 11.02%.

Ethyl 4-((3,5-dimethylphenyl)amino)-2-(4-methoxyphenyl)-5-oxo-2,5-dihydrofuran-3-carboxylate **(Table 2, 11a):** Solid powder, m.p.: 211-213°C; ^1^H-NMR (500 MHz, DMSO-d_6_): δ = 8.47 (s, 1H, NH), 7.24 (d, *J* = 7.6 Hz, 2H), 7.05 (d, *J* = 7.6 Hz, 2H), 6.80 (s, 1H), 6.64 (s, 2H), 5.76 (s, 1H), 4.07-4.12 (q, *J* = 6.9 Hz, 2H), 3.89 (s, 3H, OCH_3_), 2.41 (s, 6H), 1.28 (t, *J* = 6.9 Hz, 3H) ppm; ^13^C-NMR (125 MHz, DMSO-d_6_): δ = 14.3, 20.8, 31.8, 55.7, 61.9, 63.6, 110.8, 113.3, 114.4, 115.4, 120.2, 136.8, 137.5, 146.2, 155.3, 162.0, 167.2 ppm; Found: C, 69.39; H, 6.14; N, 3.75% C_22_H_23_NO_5_; requires: C, 69.28; H, 6.08; N, 3.67%.


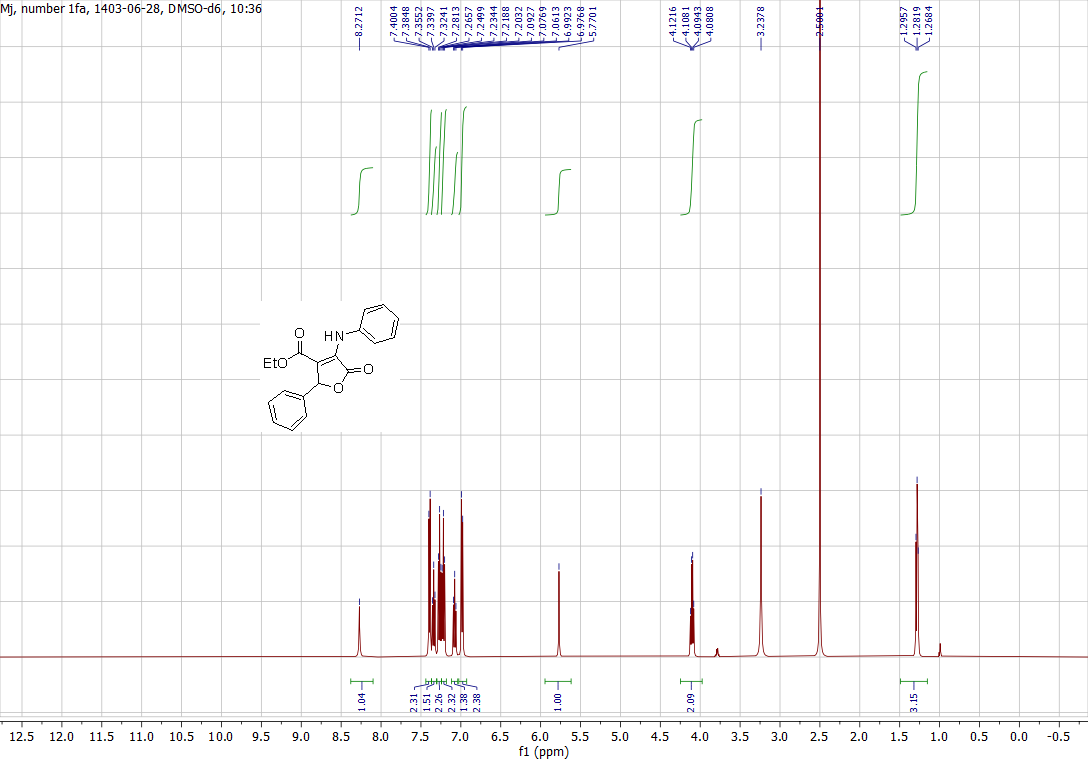


**Figure S_1_:** ^1^H-NMR spectrum of **1a**

**
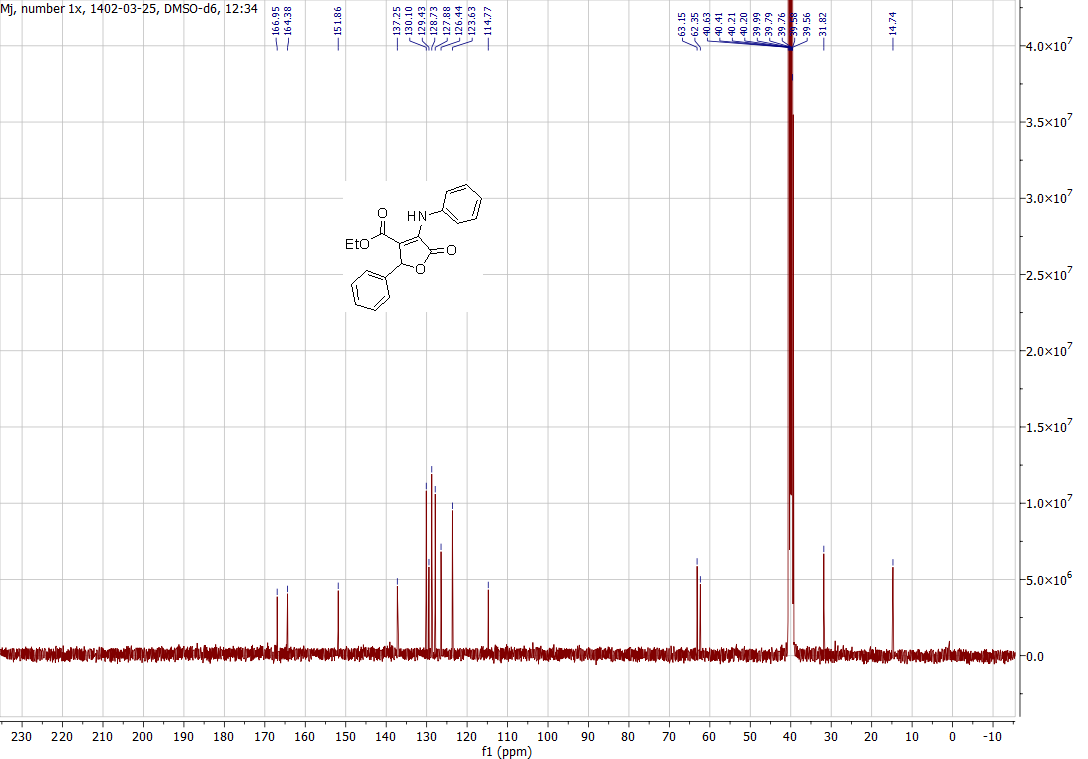
**

**Figure S_2_:** ^13^C-NMR spectrum of 1**a**


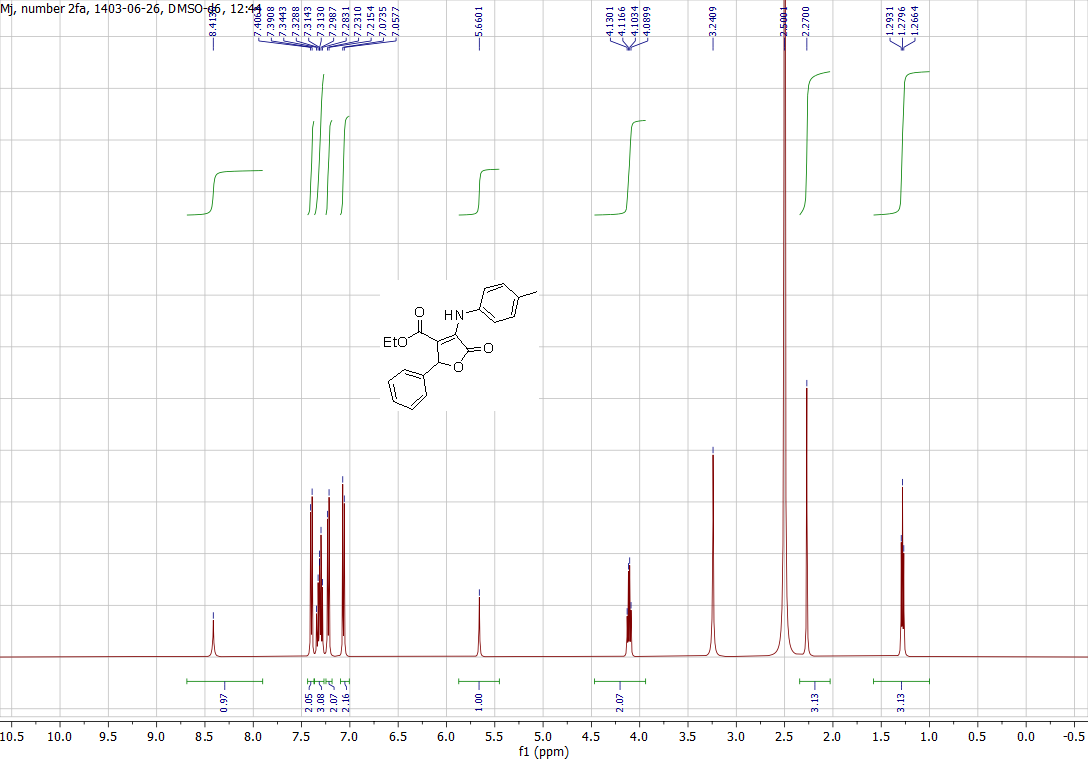


**Figure S_3_:** ^1^H-NMR spectrum of **2a**


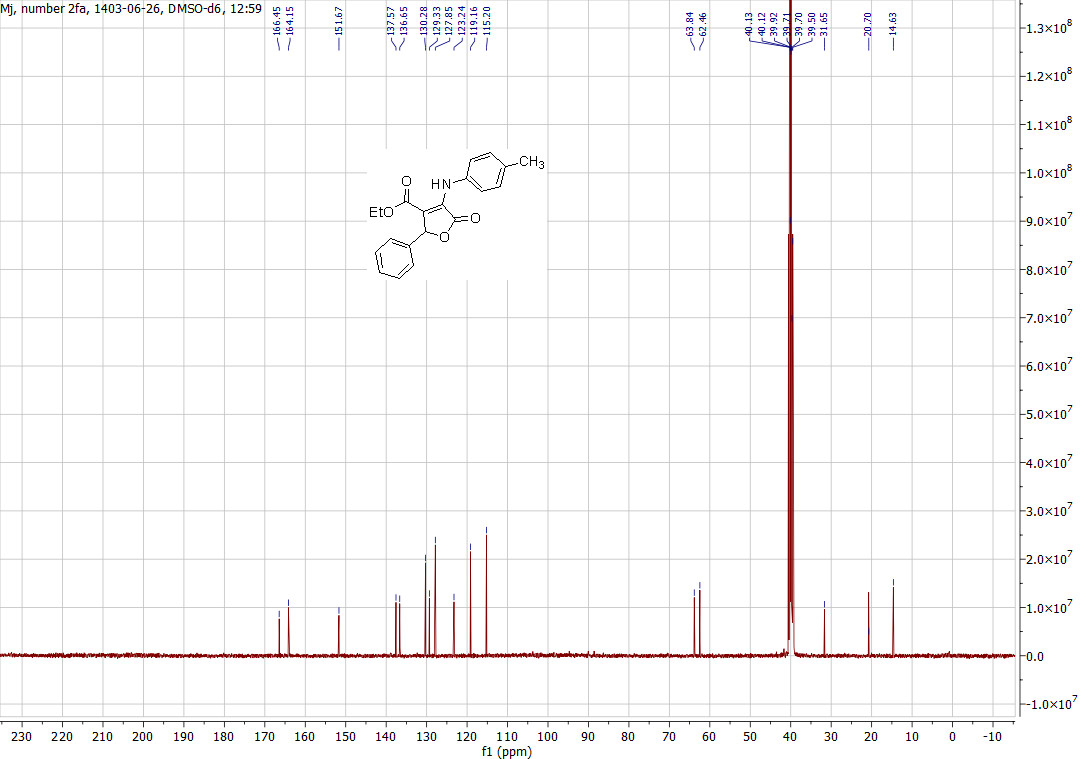


**Figure S_4_:** ^13^C-NMR spectrum of **2a**


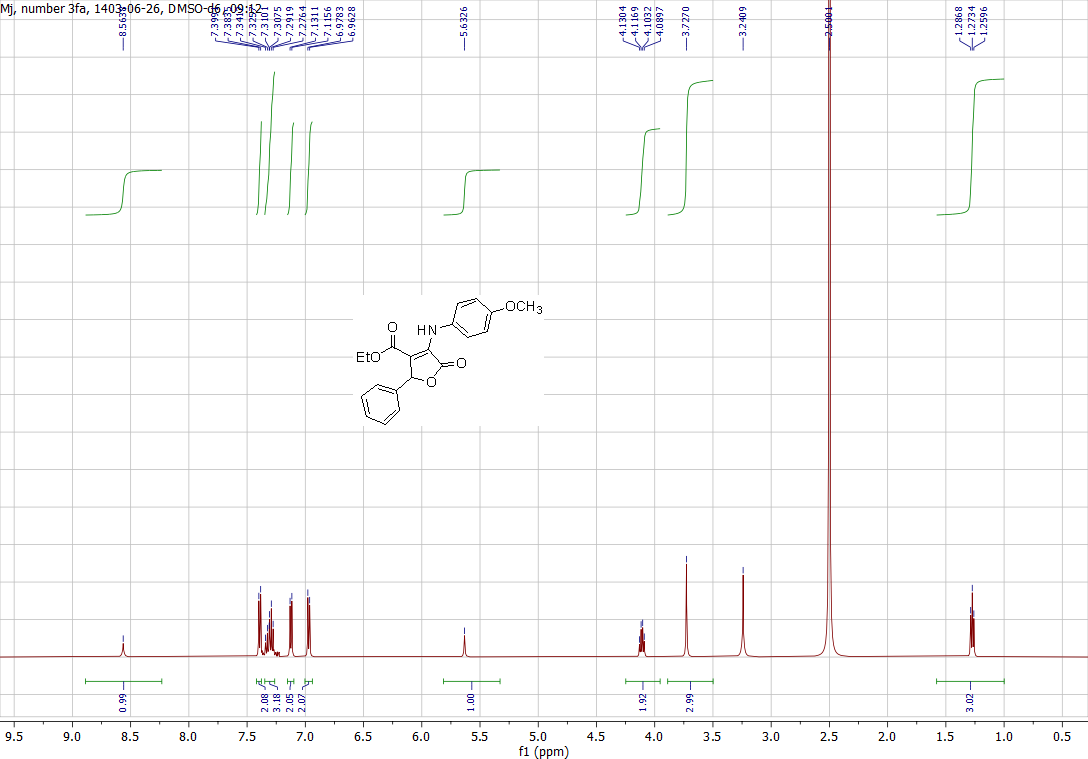


**Figure S_5_:** ^1^H-NMR spectrum of **3a**


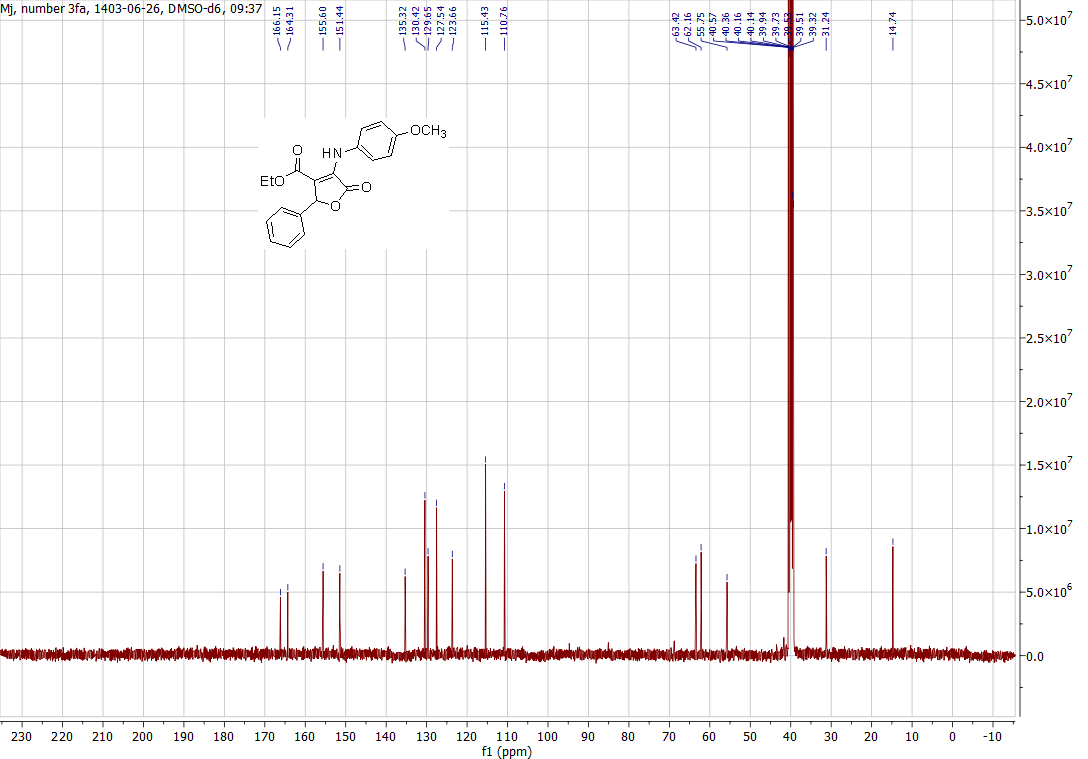


**Figure S_6_:** ^13^C-NMR spectrum of **3a**


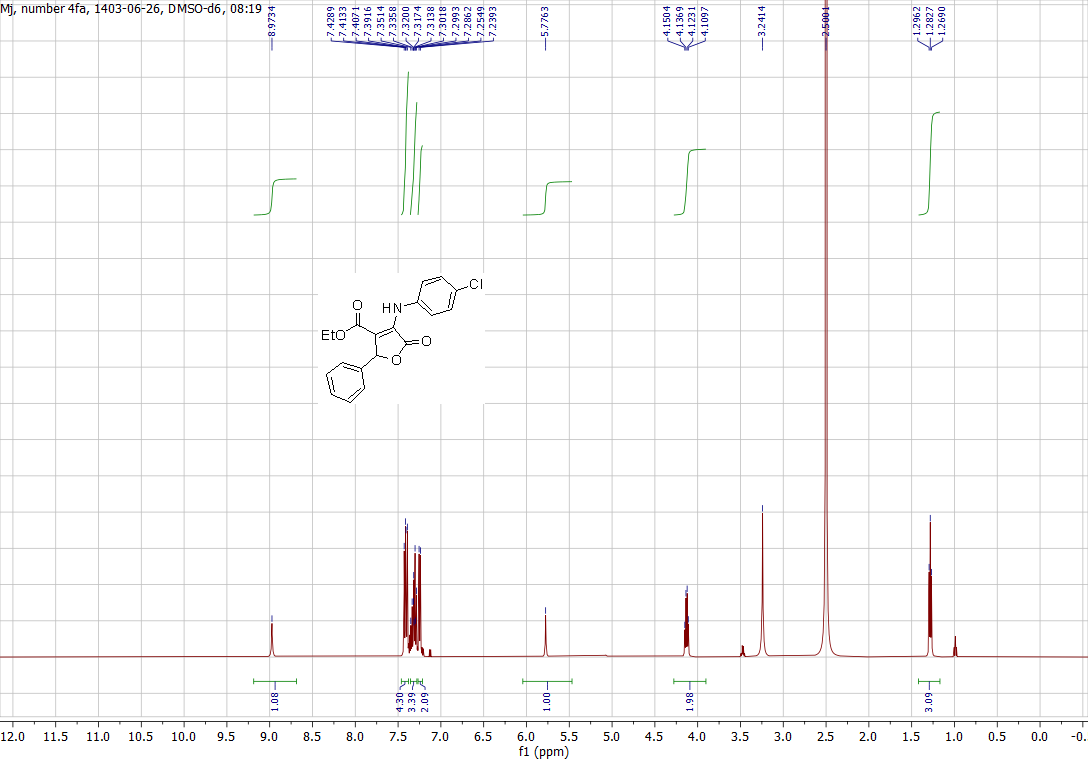


**Figure S_7_:** ^1^H-NMR spectrum of **4a**


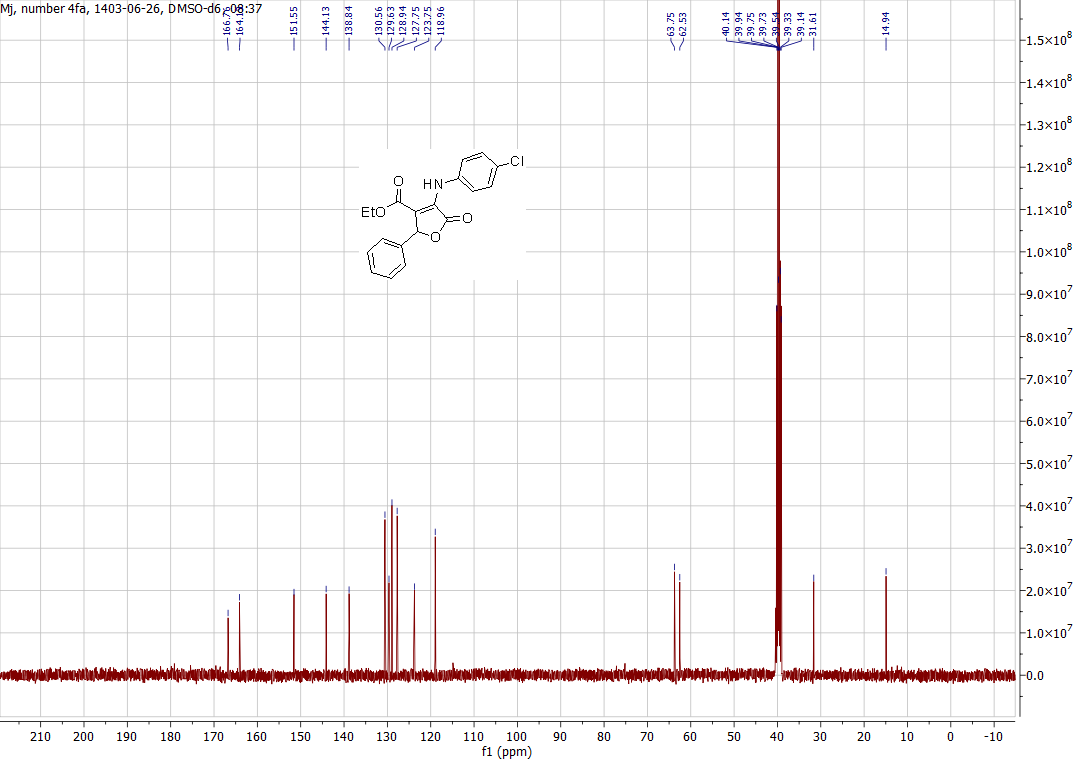


**Figure S_8_:** ^13^C-NMR spectrum of **4a**


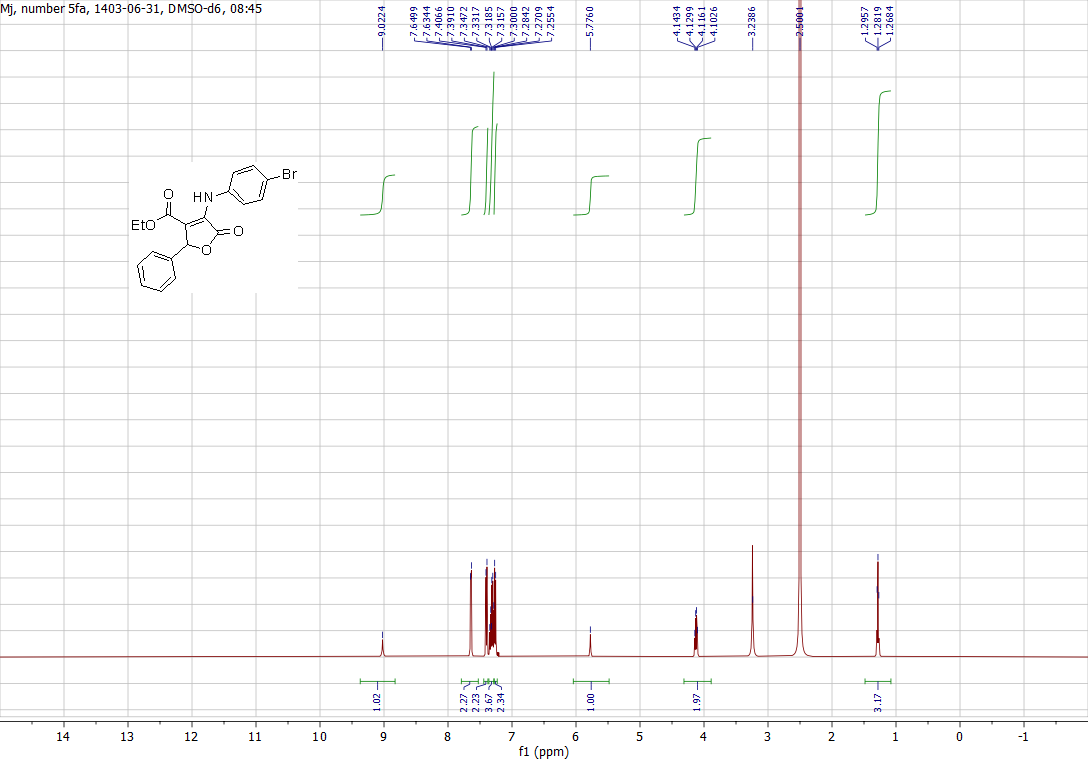


**Figure S_9_:** ^1^H-NMR spectrum of **5a**


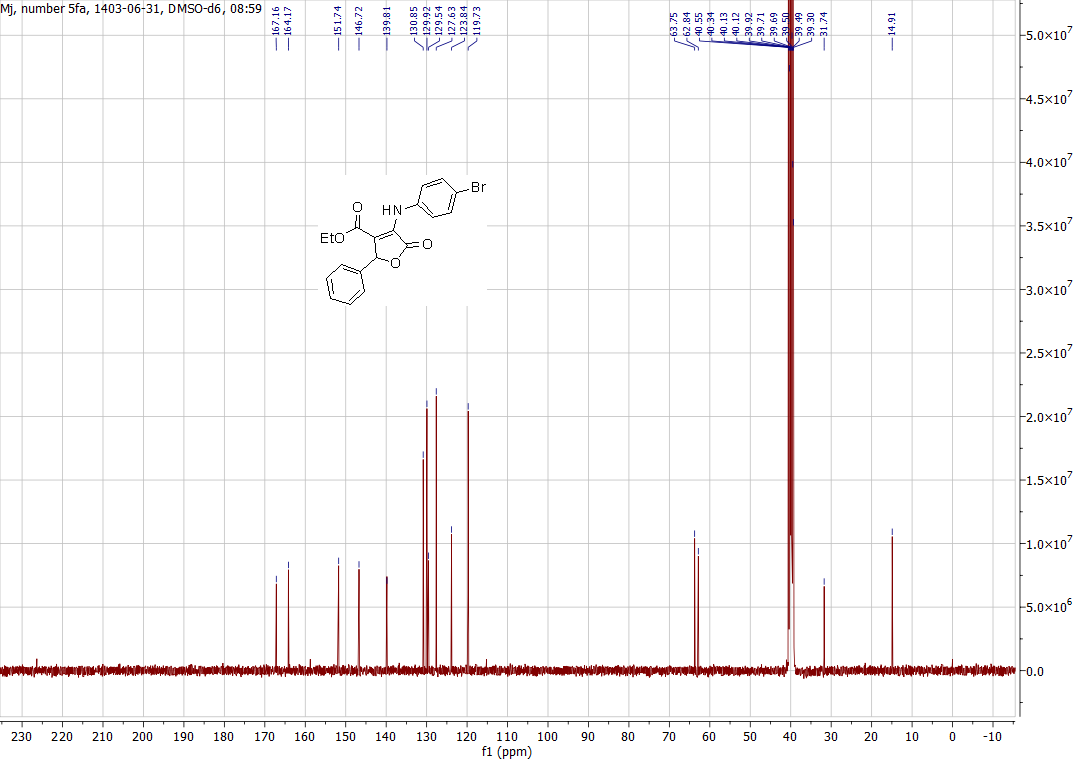


**Figure S_10_:** ^13^C-NMR spectrum of **5a**


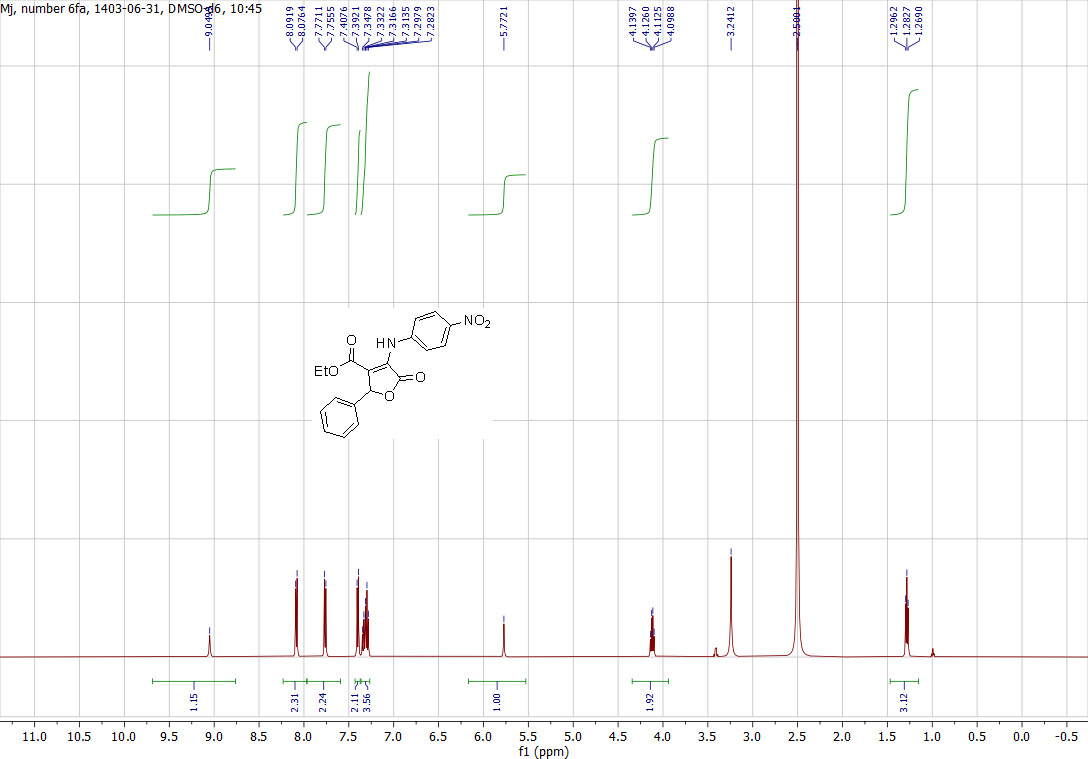


**Figure S_11_:** ^1^H-NMR spectrum of **6a**


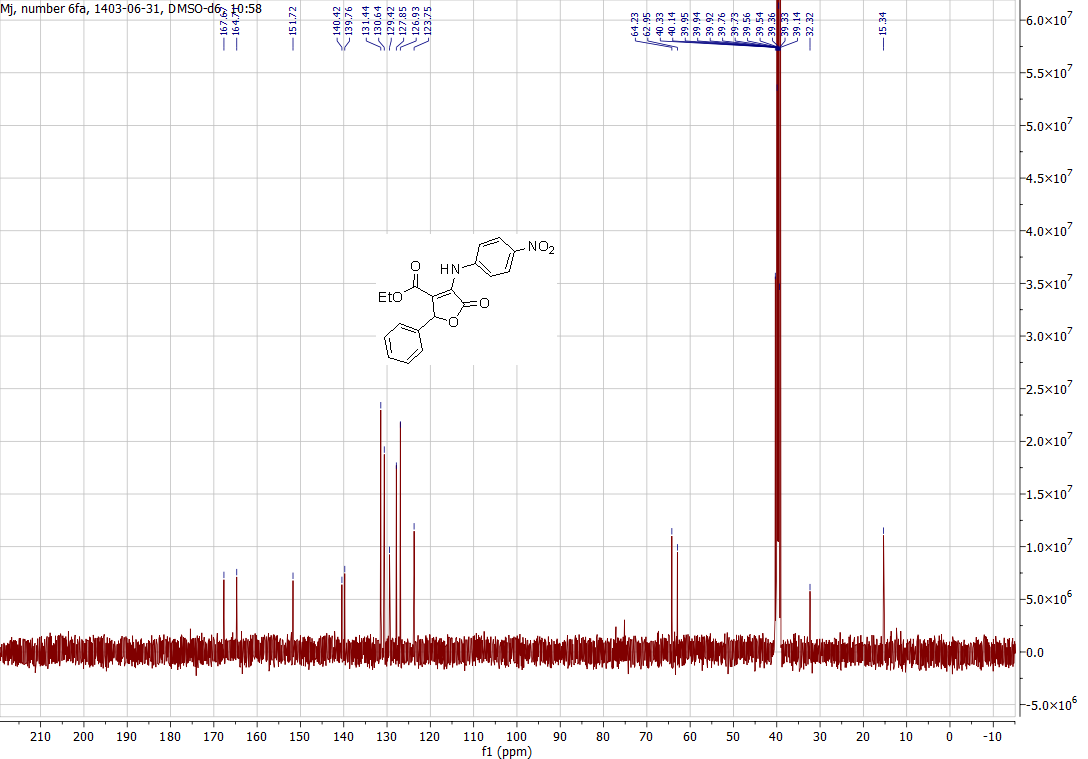


**Figure S_12_:** ^13^C-NMR spectrum of **6a**


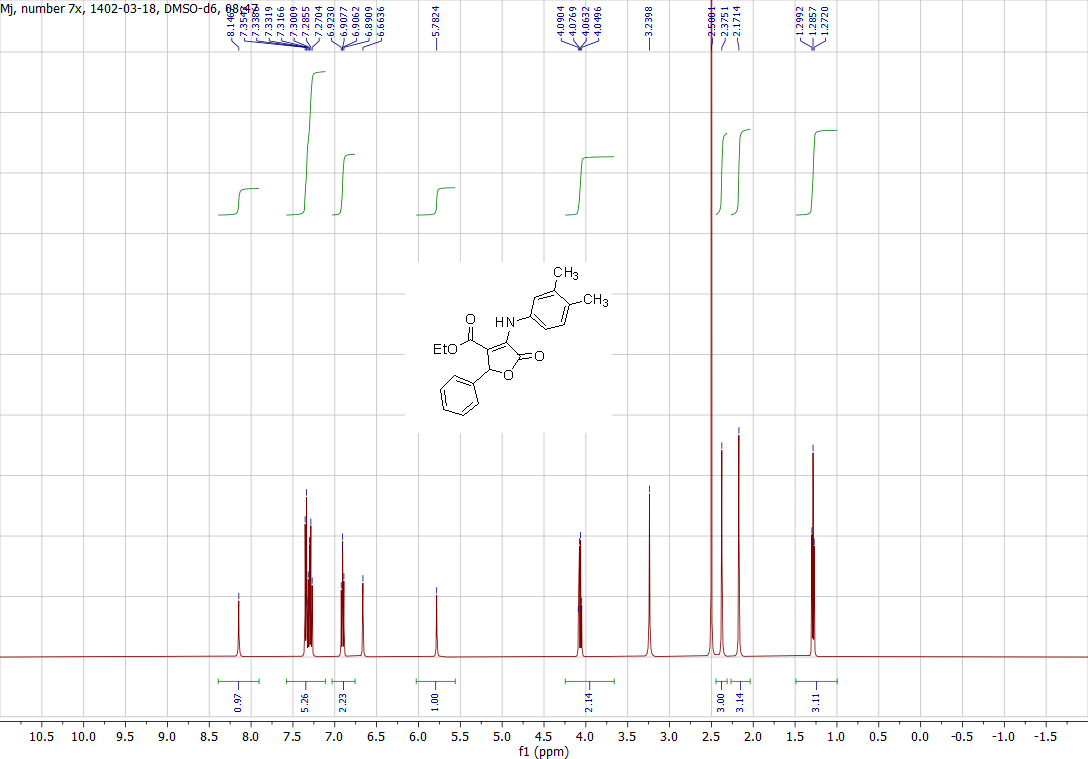


**Figure S_13_:** ^1^H-NMR spectrum of **7a**

**
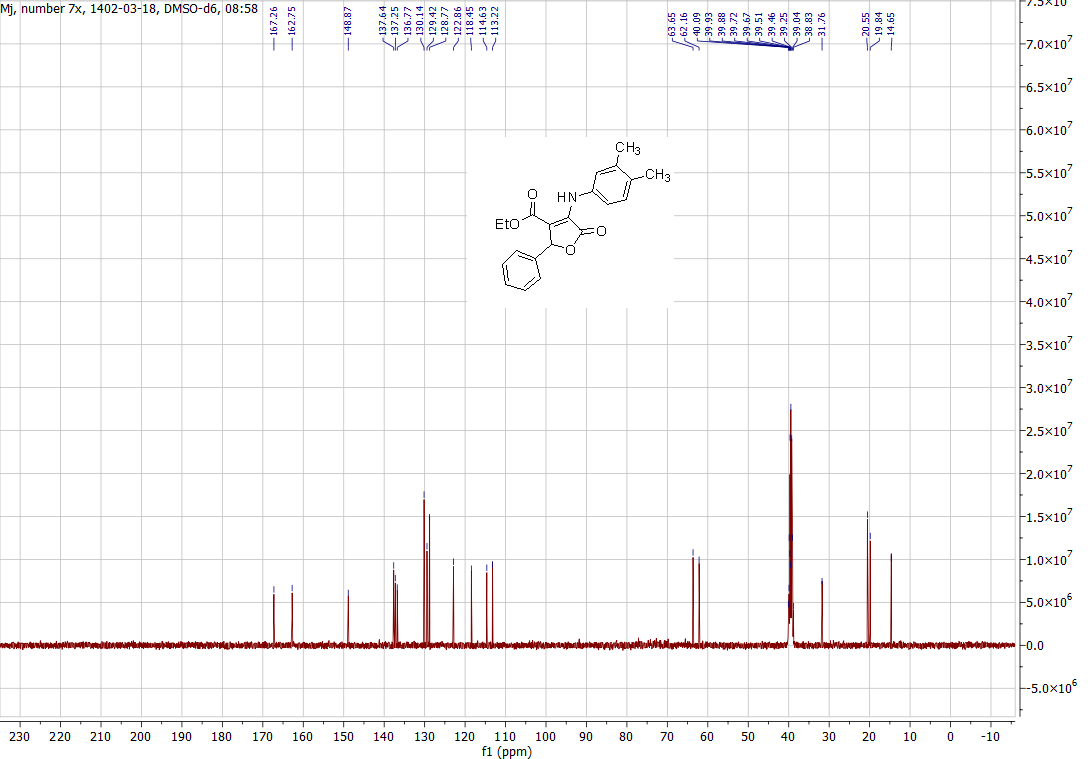
**

**Figure S_14_:** ^13^C-NMR spectrum of **7a**


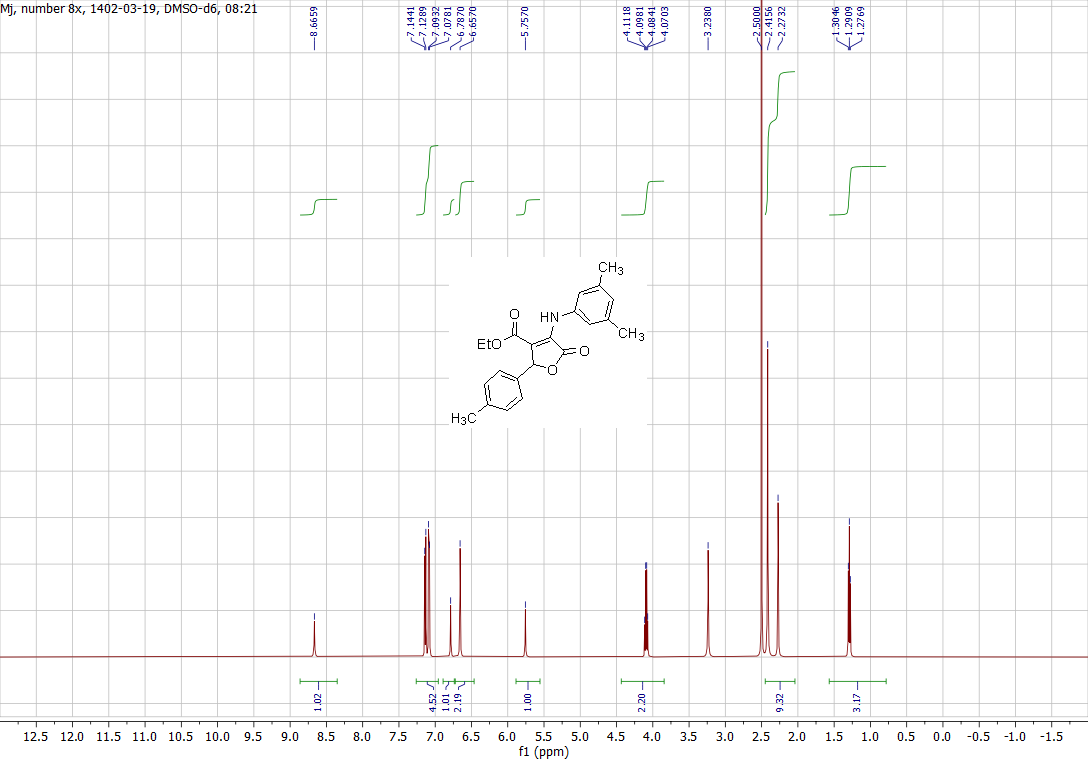


**Figure S_15_:** ^1^H-NMR spectrum of **8a**

**
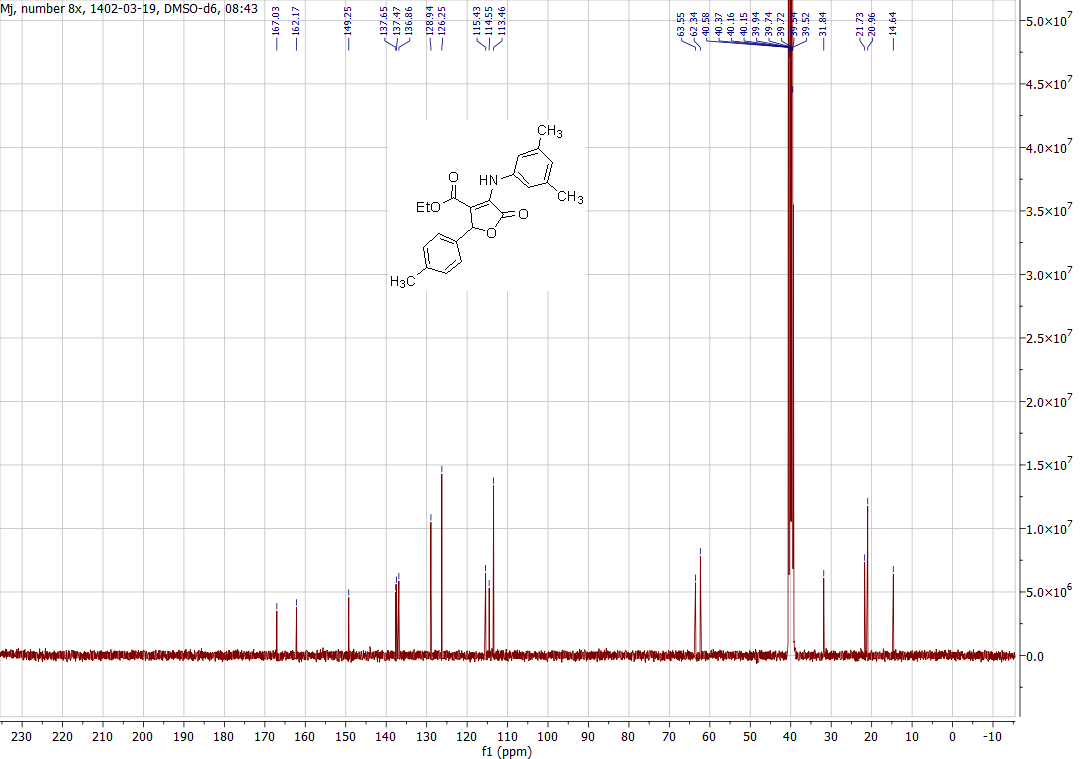
**

**Figure S_16_:** ^13^C-NMR spectrum of **8a**


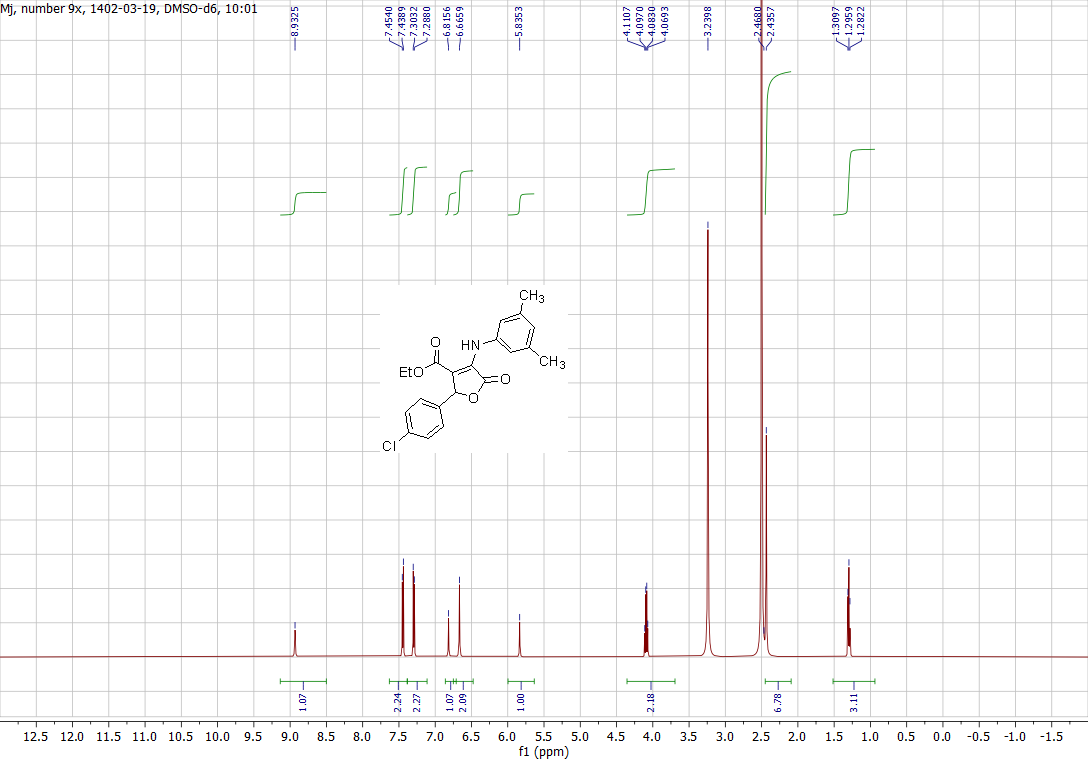


**Figure S_17_:** ^1^H-NMR spectrum of **9a**


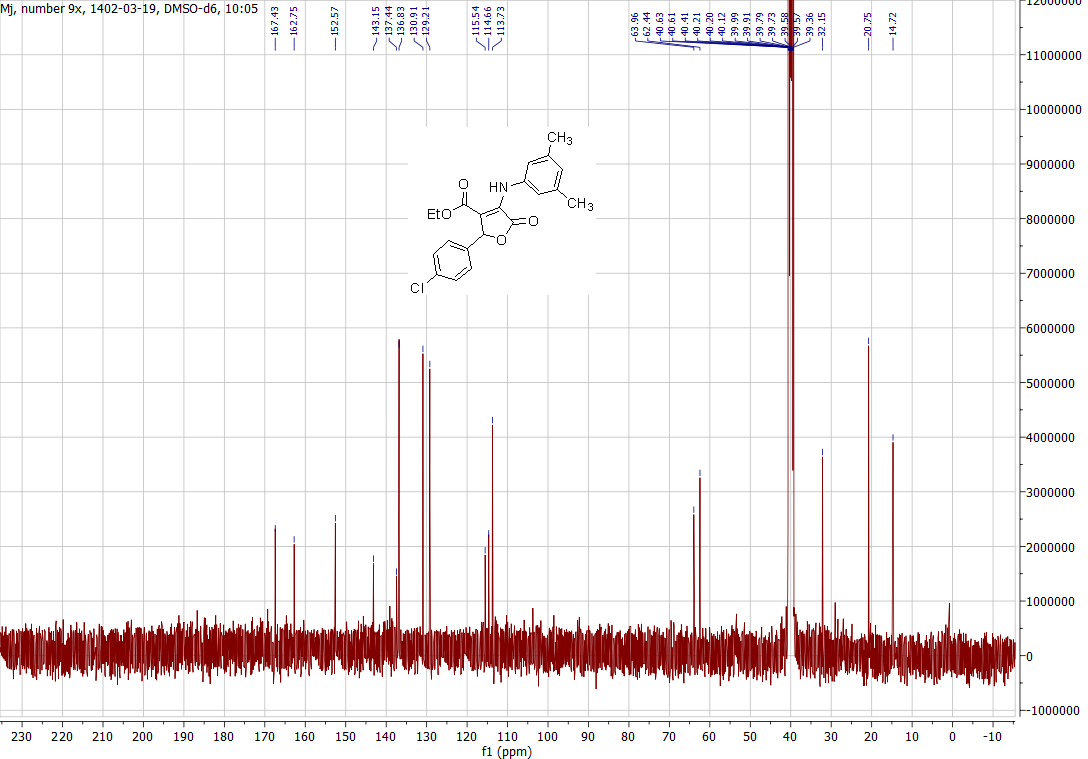


**Figure S_18_:** ^13^C-NMR spectrum of **9a**


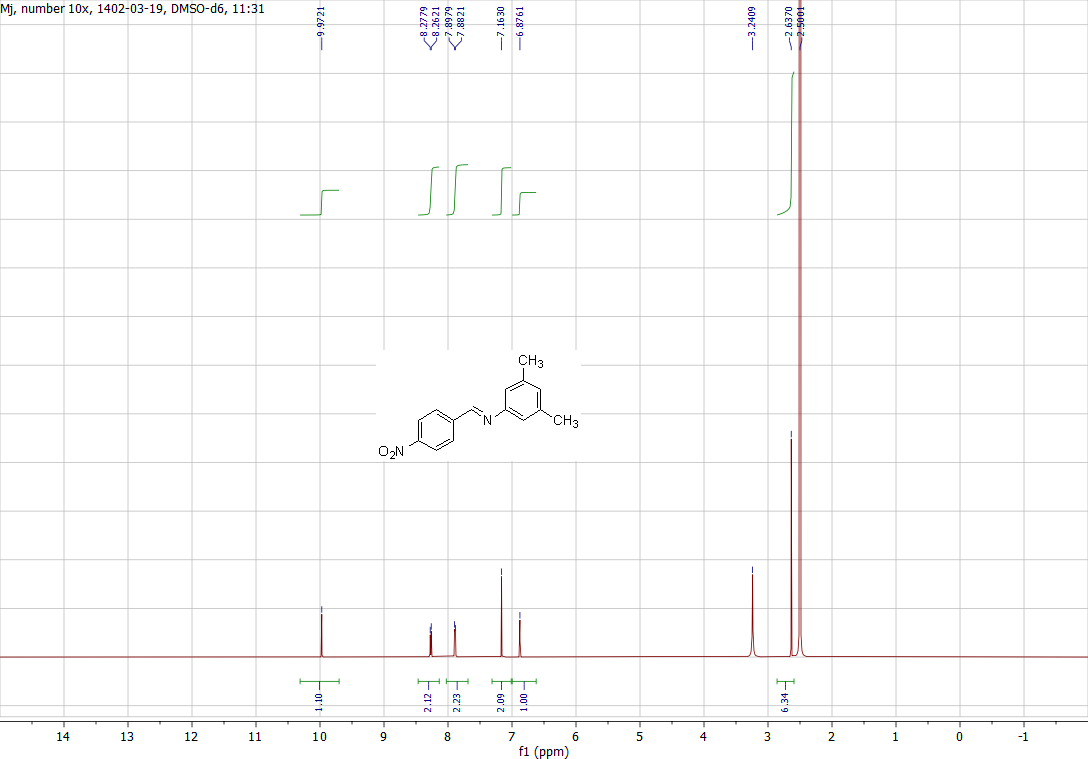


**Figure S_19_:** ^1^H-NMR spectrum of **10a**


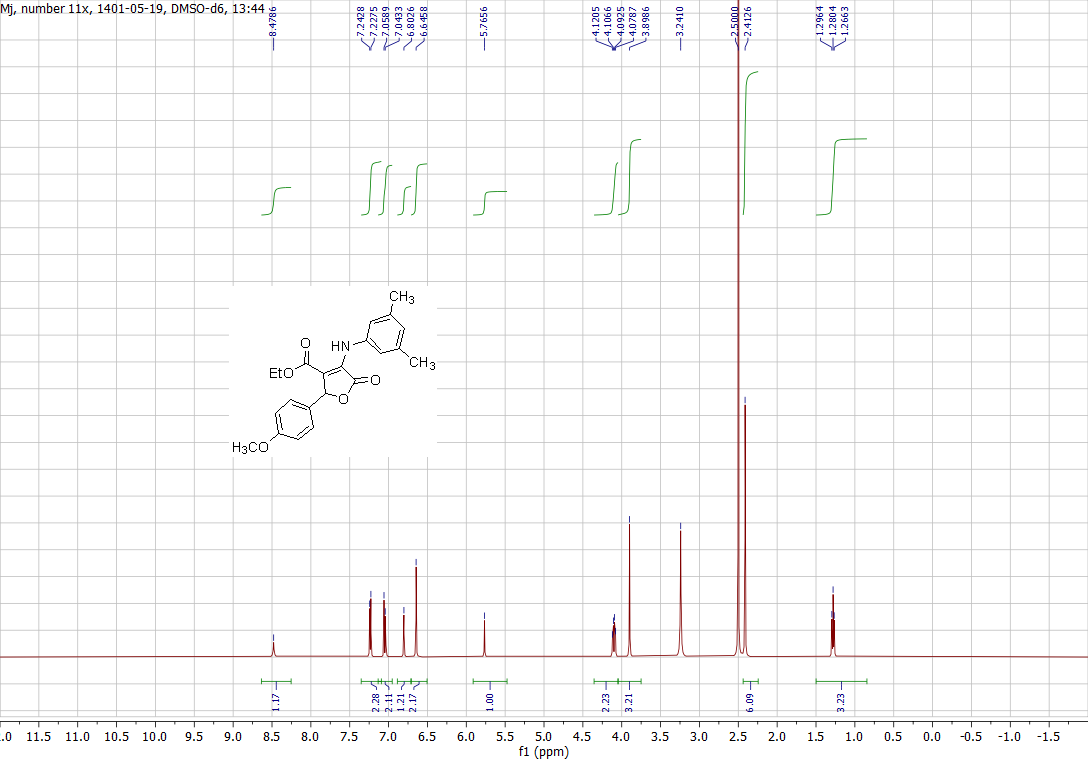


**Figure S_20_:** ^1^H-NMR spectrum of **11a**


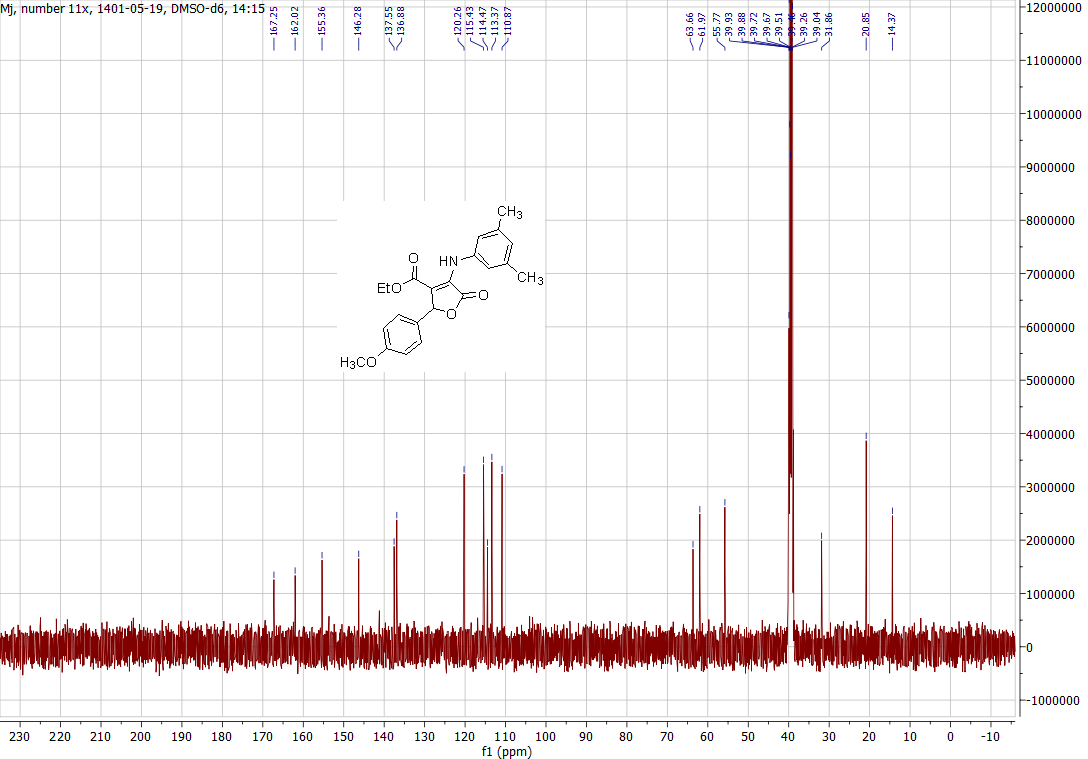


**Figure S_21_:** ^13^C-NMR spectrum of **11a**

Ethyl 1'-ethyl-2',5-dioxo-4-(phenylamino)-5H-spiro[furan-2,3'-indoline]-3-carboxylate **(Table 3, 12a):** Solid powder, m.p.: 219-221°C; ^1^H-NMR (500 MHz, DMSO-d_6_): δ = 8.95 (s, 1H), 7.47 (t, *J* = 7.8 Hz, 1H), 7.31 (d, *J* = 7.8 Hz, 1H), 7.25 (t, *J* = 7.8 Hz, 2H), 7.16 (t, *J* = 7.8 Hz, 1H), 7.06-7.10 (m, 3H), 6.96 (d, *J* = 7.8 Hz, 1H), 4.11 (q, *J* = 6.8 Hz, 2H), 3.95 (q, *J* = 7.1 Hz, 2H), 1.24-1.28 (m, 6H) ppm; ^13^C-NMR (125 MHz, DMSO-d_6_): δ = 14.2, 14.7, 48.7, 56.7, 89.4, 104.2, 114.1, 119.2, 120.3, 123.1, 124.5, 129.9, 134.5, 135.1, 143.8, 151.7, 155.1, 164.5, 169.3, 179.1 ppm; Found: C, 67.26; H, 5.02; N, 7.09% C_22_H_20_N_2_O_5_; requires: C, 67.34; H, 5.14; N, 7.14%.

Ethyl 1'-ethyl-2',5-dioxo-4-(*p*-tolylamino)-5H-spiro[furan-2,3'-indoline]-3-carboxylate **(Table 3, 13a):** Solid powder, m.p.: 223-225°C; ^1^H-NMR (500 MHz, DMSO-d_6_): δ = 8.56 (s, 1H), 7.48 (t, *J* = 7.9 Hz, 1H), 7.32 (d, *J* = 7.9 Hz, 1H), 7.14-7.19 (m, 3H), 6.97-7.01 (m, 3H), 4.13 (q, *J* = 7.0 Hz, 2H), 3.97 (q, *J* = 7.3 Hz, 2H), 2.29 (s, 3H), 1.22-1.29 (m, 6H) ppm; ^13^C-NMR (125 MHz, DMSO-d_6_): δ = 14.3, 14.6, 21.4, 48.8, 56.4, 89.6, 104.1, 107.7, 112.3, 120.1, 124.6, 129.7, 134.1, 135.3, 137.1, 143.6, 151.9, 155.4, 164.8, 169.7, 178.7 ppm; Found: C, 67.87; H, 5.53; N, 6.83% C_23_H_22_N_2_O_5_; requires: C, 67.97; H, 5.46; N, 6.89%.

Ethyl 4-((4-chlorophenyl)amino)-1'-ethyl-2',5-dioxo-5H-spiro[furan-2,3'-indoline]-3-carboxylate **(Table 3, 14a):** Solid powder, m.p.: 245-247°C; ^1^H-NMR (500 MHz, DMSO-d_6_): δ = 9.32 (s, 1H), 7.49 (t, *J* = 8.2 Hz, 1H), 7.38 (d, *J* = 8.0 Hz, 2H), 7.32 (d, *J* = 8.1 Hz, 1H), 7.17 (t, *J* = 8.2 Hz, 1H), 7.09 (d, *J* = 8.0 Hz, 2H), 6.97 (d, *J* = 8.2 Hz, 1H), 3.96-4.15 (m, 4H), 1.21-1.29 (m, 6H) ppm; ^13^C-NMR (125 MHz, DMSO-d_6_): δ = 14.4, 15.1, 49.2, 56.5, 91.2, 106.3, 118.7, 120.3, 125.2, 127.9, 129.8, 134.4, 136.1, 143.7, 144.2, 152.3, 156.1, 165.7, 170.1, 179.4 ppm; Found: C, 61.86; H, 4.55; N, 6.63% C_22_H_19_ClN_2_O_5_; requires: C, 61.90; H, 4.49; N, 6.56%.

Ethyl 1'-ethyl-2',5-dioxo-4-((4-methoxyphenyl)amino)-5H-spiro[furan-2,3'-indoline]-3-carboxylate **(Table 3, 15a):** Solid powder, m.p.: 236-238°C; ^1^H-NMR (500 MHz, DMSO-d_6_): δ = 9.12 (s, 1H), 7.47 (t, *J* = 8.0 Hz, 1H), 7.31 (d, *J* = 8.0 Hz, 1H), 7.16 (t, *J* = 8.0 Hz, 1H), 7.03 (d, *J* = 8.1 Hz, 2H), 6.96 (d, *J* = 8.0 Hz, 1H), 6.87 (d, *J* = 8.1 Hz, 2H), 4.09 (q, *J* = 6.9 Hz, 2H), 3.96 (q, *J* = 6.8 Hz, 2H), 3.74 (s, 3H), 1.20-1.29 (m, 6H) ppm; ^13^C-NMR (125 MHz, DMSO-d_6_): δ = 14.4, 14.9, 48.3, 55.4, 56.3, 89.2, 103.2, 106.2, 110.3, 119.8, 124.1, 129.9, 134.4, 134.8, 143.2, 151.1, 155.1, 155.7, 164.1, 167.3, 176.1 ppm; Found: C, 65.33; H, 5.26; N, 6.58% C_23_H_22_N_2_O_6_; requires: C, 65.40; H, 5.25; N, 6.63%.

Ethyl 4-((3,5-dimethylphenyl)amino)-1'-ethyl-2',5-dioxo-5H-spiro[furan-2,3'-indoline]-3-carboxylate **(Table 3, 16a):** Solid powder, m.p.: 249-251°C; ^1^H-NMR (500 MHz, DMSO-d_6_): δ = 8.41 (s, 1H), 7.46 (t, *J* = 8.0 Hz, 1H), 7.30 (d, *J* = 8.0 Hz, 1H), 7.15 (t, *J* = 8.0 Hz, 1H), 6.95 (d, *J* = 8.0 Hz, 1H), 6.88 (s, 1H), 6.85 (s, 2H), 4.03 (q, *J* = 7.0 Hz, 2H), 3.92 (q, *J* = 7.1 Hz, 2H), 2.27 (s, 6H), 1.20-1.27 (m, 6H) ppm; ^13^C-NMR (125 MHz, DMSO-d_6_): δ = 14.3, 15.0, 21.6, 48.6, 56.5, 89.0, 105.2, 111.2, 116.7, 119.7, 124.2, 129.7, 134.1, 135.8, 137.3, 143.1, 151.0, 155.5, 163.8, 166.0, 178.1 ppm; Found: C, 68.45; H, 5.66; N, 6.58% C_24_H_24_N_2_O_5_; requires: C, 68.56; H, 5.75; N, 6.66%.

Ethyl 4-((3,4-dimethylphenyl)amino)-1'-ethyl-2',5-dioxo-5H-spiro[furan-2,3'-indoline]-3-carboxylate **(Table 3, 17a):** Solid powder, m.p.: 247-249°C; ^1^H-NMR (500 MHz, DMSO-d_6_): δ = 7.89 (s, 1H), 7.46 (t, *J* = 8.2 Hz, 1H), 7.31 (d, *J* = 8.2 Hz, 1H), 7.15 (t, *J* = 8.2 Hz, 1H), 7.07 (d, *J* = 7.8 Hz, 1H), 7.04 (d, *J* = 7.8 Hz, 1H), 6.98 (s, 1H), 6.95 (d, *J* = 8.2 Hz, 1H), 4.04 (q, *J* = 7.0 Hz, 2H), 3.93 (q, *J* = 7.0 Hz, 2H), 2.78 (s, 3H), 2.26 (s, 3H), 1.27 (t, *J* = 7.0 Hz, 3H), 1.22 (t, *J* = 7.0 Hz, 3H) ppm; ^13^C-NMR (125 MHz, DMSO-d_6_): δ = 14.3, 14.9, 20.8, 21.3, 48.7, 56.5, 89.4, 105.7, 109.9, 112.4, 116.1, 119.8, 124.5, 129.8, 134.2, 135.4, 136.8, 137.1, 143.3, 151.1, 155.6, 163.7, 166.2, 177.7 ppm; Found: C, 68.47; H, 5.69; N, 6.55% C_24_H_24_N_2_O_5_; requires: C, 68.56; H, 5.75; N, 6.66%.

Ethyl 1'-ethyl-4-((4-ethylphenyl)amino)-2',5-dioxo-5H-spiro[furan-2,3'-indoline]-3-carboxylate **(Table 3, 18a):** Solid powder, m.p.: 231-233°C; ^1^H-NMR (500 MHz, DMSO-d_6_): δ = 7.96 (s, 1H), 7.47 (t, *J* = 8.0 Hz, 1H), 7.32 (d, *J* = 8.0 Hz, 1H), 7.16 (t, *J* = 8.0 Hz, 1H), 7.09 (d, *J* = 7.8 Hz, 2H), 7.01 (d, *J* = 7.8 Hz, 2H), 6.96 (d, *J* = 8.0 Hz, 1H), 4.08 (q, *J* = 6.8 Hz, 2H), 3.96 (q, *J* = 6.8 Hz, 2H), 2.19 (q, *J* = 7.0 Hz, 2H), 1.22-1.30 (m, 6H), 1.04 (t, *J* = 7.0 Hz, 3H) ppm; ^13^C-NMR (125 MHz, DMSO-d_6_): δ = 13.9, 14.4, 15.0, 24.8, 48.6, 56.5, 89.1, 104.5, 111.1, 115.3, 119.9, 124.5, 129.8, 134.2, 135.4, 137.7, 143.4, 151.2, 155.6, 164.2, 168.7, 177.1 ppm; Found: C, 68.66; H, 5.89; N, 6.74% C_24_H_24_N_2_O_5_; requires: C, 68.56; H, 5.75; N, 6.66%.

Ethyl 1'-benzyl-2',5-dioxo-4-(*p*-tolylamino)-5H-spiro[furan-2,3'-indoline]-3-carboxylate **(Table 3, 19a):** Solid powder, m.p.: 261-263°C; ^1^H-NMR (500 MHz, DMSO-d_6_): δ = 7.76 (s, 1H), 7.49 (t, *J* = 8.0 Hz, 1H), 7.34 (d, *J* = 8.0 Hz, 1H), 7.13-7.25 (m, 6H), 7.03 (d, *J* = 8.0 Hz, 2H), 6.97 (d, *J* = 8.0 Hz, 1H), 6.91 (d, *J* = 8.0 Hz, 2H), 4.58 (d, *J* = 10.8 Hz, 1H), 4.48 (d, *J* = 10.8 Hz, 1H), 4.16 (q, *J* = 6.8 Hz, 2H), 2.28 (s, 3H), 1.31 (t, *J* = 6.8 Hz, 3H) ppm; ^13^C-NMR (125 MHz, DMSO-d_6_): δ = 15.8, 21.4, 56.7, 74.6, 91.7, 105.7, 112.2, 116.3, 121.2, 124.7, 127.6, 128.8, 129.1, 129.8, 134.6, 136.7, 137.8, 138.6, 143.2, 151.5, 155.1, 167.2, 169.7, 177.7 ppm; Found: C, 71.85; H, 5.22; N, 5.91% C_28_H_24_N_2_O_5_; requires: C, 71.78; H, 5.16; N, 5.98%.

Ethyl 1'-benzyl-4-((4-chlorophenyl)amino)-2',5-dioxo-5H-spiro[furan-2,3'-indoline]-3-carboxylate **(Table 3, 20a):** Solid powder, m.p.: 271-273°C; ^1^H-NMR (500 MHz, DMSO-d_6_): δ = 8.87 (s, 1H), 7.50 (t, *J* = 8.2 Hz, 1H), 7.41 (d, *J* = 7.8 Hz, 2H), 7.35 (d, *J* = 8.2 Hz, 1H), 7.14-7.26 (m, 6H), 7.08 (d, *J* = 7.8 Hz, 2H), 6.96 (d, *J* = 8.2 Hz, 1H), 4.60 (d, *J* = 10.9 Hz, 1H), 4.51 (d, *J* = 10.8 Hz, 1H), 4.14 (q, *J* = 6.8 Hz, 2H), 1.30 (t, *J* = 6.9 Hz, 3H) ppm; ^13^C-NMR (125 MHz, DMSO-d_6_): δ = 16.1, 57.1, 74.9, 92.3, 106.1, 121.3, 124.8, 127.2, 127.8, 128.4, 129.0, 129.4, 130.1, 134.7, 136.6, 138.7, 143.6, 144.2, 151.4, 155.3, 167.7, 170.1, 178.4 ppm; Found: C, 66.42; H, 4.39; N, 5.67% C_27_H_21_ClN_2_O_5_; requires: C, 66.33; H, 4.33; N, 5.73%.

Ethyl 1'-benzyl-4-((4-methoxyphenyl)amino)-2',5-dioxo-5H-spiro[furan-2,3'-indoline]-3-carboxylate **(Table 3, 21a):** Solid powder, m.p.: 277-279°C; ^1^H-NMR (500 MHz, DMSO-d_6_): δ = 8.47 (s, 1H), 7.49 (t, *J* = 8.2 Hz, 1H), 7.35 (d, *J* = 8.1 Hz, 1H), 7.25 (d, *J* = 7.8 Hz, 2H), 713-721 (m, 4H), 6.96 (d, *J* = 8.1 Hz, 1H), 6.91 (d, *J* = 7.8 Hz, 2H), 6.81 (d, *J* = 7.8 Hz, 2H), 4.60 (d, *J* = 11.0 Hz, 1H), 4.50 (d, *J* = 11.0 Hz, 1H), 4.11 (q, *J* = 6.8 Hz, 2H), 3.69 (s, 3H), 1.30 (t, *J* = 6.8 Hz, 3H) ppm; ^13^C-NMR (125 MHz, DMSO-d_6_): δ = 16.0, 55.2, 56.8, 74.3, 92.0, 105.8, 107.3, 110.8, 121.0, 124.4, 127.4, 128.2, 129.2, 130.0, 134.3, 135.1, 138.5, 143.4, 151.2, 155.4, 155.7, 166.3, 169.1, 175.2 ppm; Found: C, 69.49; H, 5.11; N, 5.68% C_28_H_24_N_2_O_6_; requires: C, 69.41; H, 4.99; N, 5.78%.

Ethyl 1'-benzyl-5'-chloro-2',5-dioxo-4-(p-tolylamino)-5H-spiro[furan-2,3'-indoline]-3-carboxylate **(Table 3, 22a):** Solid powder, m.p.: 281-283°C; ^1^H-NMR (500 MHz, DMSO-d_6_): δ = 8.87 (s, 1H), 6.93-7.29 (m, 12H), 4.62 (d, *J* = 11.0 Hz, 1H), 4.53 (d, *J* = 11.0 Hz, 1H), 4.10 (q, *J* = 6.8 Hz, 2H), 2.25 (s, 3H), 1.28 (t, *J* = 6.8 Hz, 3H) ppm; ^13^C-NMR (125 MHz, DMSO-d_6_): δ = 16.3, 21.2, 56.7, 74.7, 93.1, 106.8, 112.3, 116.8, 122.3, 126.7, 127.4, 128.4, 129.3, 132.3, 135.3, 136.4, 137.5, 143.6, 144.6, 151.5, 155.6, 166.7, 170.1, 176.4 ppm; Found: C, 66.77; H, 4.55; N, 5.49% C_28_H_23_ClN_2_O_5_; requires: C, 66.87; H, 4.61; N, 5.57%.

Ethyl 1'-benzyl-5'-chloro-4-((4-chlorophenyl)amino)-2',5-dioxo-5H-spiro[furan-2,3'-indoline]-3-carboxylate **(Table 3, 23a):** Solid powder, m.p.: 278-280°C; ^1^H-NMR (500 MHz, DMSO-d_6_): δ = 8.96 (s, 1H), 7.41 (d, *J* = 7.8 Hz, 2H), 7.14-7.30 (m, 7H), 7.09 (d, *J* = 7.8 Hz, 2H), 6.96 (d, *J* = 8.0 Hz, 1H), 4.61 (d, *J* = 10.6 Hz, 1H), 4.51 (d, *J* = 10.6 Hz, 1H), 4.11 (q, *J* = 6.8 Hz, 2H), 1.29 (t, *J* = 6.9 Hz, 3H) ppm; ^13^C-NMR (125 MHz, DMSO-d_6_): δ = 16.4, 56.6, 76.1, 94.2, 106.7, 122.1, 126.4, 127.1, 127.6, 128.1, 128.5, 129.4, 132.1, 135.4, 136.8, 143.2, 143.8, 144.5, 151.6, 155.7, 166.5, 170.6, 178.4 ppm; Found: C, 61.84; H, 3.77; N, 5.26% C_27_H_20_Cl_2_N_2_O_5_; requires: C, 61.96; H, 3.85; N, 5.35%.

Ethyl 1'-benzyl-5'-chloro-4-((4-methoxyphenyl)amino)-2',5-dioxo-5H-spiro[furan-2,3'-indoline]-3-carboxylate **(Table 3, 24a):** Solid powder, m.p.: 272-274°C; ^1^H-NMR (500 MHz, DMSO-d_6_): δ = 8.56 (s, 1H), 7.13-7.29 (m, 7H), 6.96 (d, *J* = 8.2 Hz, 1H), 6.89 (d, *J* = 7.8 Hz, 2H), 6.77 (d, *J* = 7.9 Hz, 2H), 4.59 (d, *J* = 10.8 Hz, 1H), 4.49 (d, *J* = 10.8 Hz, 1H), 4.09 (q, *J* = 6.9 Hz, 2H), 3.89 (s, 3H), 1.28 (t, *J* = 6.9 Hz, 3H) ppm; ^13^C-NMR (125 MHz, DMSO-d_6_): δ = 15.8, 55.4, 56.1, 75.2, 93.8, 106.1, 108.4, 112.3, 121.7, 126.0, 127.3, 128.4, 129.2, 132.0, 135.1, 135.4, 143.1, 144.3, 151.2, 155.3, 155.5, 165.2, 169.6, 177.1 ppm; Found: C, 64.75; H, 4.41; N, 5.33% C_28_H_23_ClN_2_O_6_; requires: C, 64.81; H, 4.47; N, 5.40%.

Ethyl 1'-benzyl-5'-chloro-4-((4-ethylphenyl)amino)-2',5-dioxo-5H-spiro[furan-2,3'-indoline]-3-carboxylate **(Table 3, 25a):** Solid powder, m.p.: 275-277°C; ^1^H-NMR (500 MHz, DMSO-d_6_): δ = 8.36 (s, 3H), 6.95-7.30 (m, 12H), 4.59 (d, *J* = 10.8 Hz, 1H), 4.46 (d, *J* = 10.8 Hz, 1H), 4.08 (q, *J* = 6.8 Hz, 2H), 2.35 (q, *J* = 6.8 Hz, 2H), 1.29 (t, *J* = 6.8 Hz, 3H), 0.98 (t, *J* = 6.8 Hz, 3H) ppm; ^13^C-NMR (125 MHz, DMSO-d_6_): δ = 14.6, 15.7, 24.3, 56.1, 75.1, 93.5, 106.1, 112.4, 117.3, 121.8, 125.9, 127.3, 128.4, 129.2, 132.1, 135.3, 135.8, 136.8, 143.1, 144.4, 151.2, 155.5, 165.6, 169.1, 177.4 ppm; Found: C, 67.43; H, 4.96; N, 5.48% C_29_H_25_ClN_2_O_5_; requires: C, 67.38; H, 4.87; N, 5.42%.

Ethyl 1'-benzyl-5',7'-dichloro-4-((3,5-dimethylphenyl)amino)-2',5-dioxo-5H-spiro[furan-2,3'-indoline]-3-carboxylate **(Table 3, 26a):** Solid powder, m.p.: 279-281°C; ^1^H-NMR (500 MHz, DMSO-d_6_): δ = 8.68 (s, 1H, NH), 8.03 (s, 1H), 7.89 (s, 1H), 7.31 (d, *J* = 7.6 Hz, 2H), 7.24 (t, *J* = 7.6 Hz, 1H), 7.17 (t, *J* = 7.6 Hz, 2H), 6.86 (s, 1H), 6.71 (s, 2H), 4.61 (d, *J* = 10.8 Hz, 1H), 4.53 (d, *J* = 10.8 Hz, 1H), 4.11 (q, *J* = 6.8 Hz, 2H), 2.42 (s, 6H), 1.30 (t, *J* = 6.8 Hz, 3H) ppm; ^13^C-NMR (125 MHz, DMSO-d_6_): δ = 15.9, 19.8, 57.4, 75.3, 94.4, 107.3, 112.4, 113.6, 115.4, 122.6, 127.1, 128.4, 129.6, 132.4, 136.4, 136.8, 137.5, 143.6, 144.1, 152.5, 155.7, 165.5, 168.7, 175.4 ppm; Found: C, 63.24; H, 4.46; N, 5.17% C_29_H_24_Cl_2_N_2_O_5_; requires: C, 63.17; H, 4.39; N, 5.08%.

Ethyl 1'-benzyl-5',7'-dichloro-4-((3,4-dimethylphenyl)amino)-2',5-dioxo-5H-spiro[furan-2,3'-indoline]-3-carboxylate **(Table 3, 27a):** Solid powder, m.p.: 283-285°C; ^1^H-NMR (500 MHz, DMSO-d_6_): δ = 8.53 (s, 1H, NH), 8.04 (s, 1H), 7.91 (s, 1H), 7.32 (d, *J* = 7.6 Hz, 2H), 7.24 (t, *J* = 7.6 Hz, 1H), 7.18 (t, *J* = 7.6 Hz, 2H), 7.06 (d, *J* = 7.8 Hz, 1H), 6.96 (d, *J* = 7.8 Hz, 1H), 6.73 (s, 1H), 4.63 (d, *J* = 11.0 Hz, 1H), 4.54 (d, *J* = 11.0 Hz, 1H), 4.10 (q, *J* = 6.8 Hz, 2H), 2.23 (s, 3H), 2.17 (s, 3H), 1.30 (t, *J* = 6.8 Hz, 3H) ppm; ^13^C-NMR (125 MHz, DMSO-d_6_): δ = 15.8, 19.6, 20.4, 57.6, 75.4, 95.6, 107.9, 112.9, 115.4, 116.6, 118.8, 122.6, 127.1, 128.3, 129.4, 132.6, 136.4, 136.5, 137.4, 143.9, 144.4, 152.4, 155.3, 164.1, 167.4, 176.2 ppm; Found: C, 63.29; H, 4.42; N, 5.09% C_29_H_24_Cl_2_N_2_O_5_; requires: C, 63.17; H, 4.39; N, 5.08%.

Ethyl 1'-benzyl-5',7'-dichloro-4-((3,5-dimethoxyphenyl)amino)-2',5-dioxo-5H-spiro[furan-2,3'-indoline]-3-carboxylate **(Table 3, 28a):** Solid powder, m.p.: 269-271°C; ^1^H-NMR (500 MHz, DMSO-d_6_): δ = 8.19 (s, 1H, NH), 8.00 (s, 1H), 7.90 (s, 1H), 7.31 (d, *J* = 7.8 Hz, 2H), 7.25 (t, *J* = 7.8 Hz, 1H), 7.16 (t, *J* = 7.8 Hz, 2H), 6.68 (s, 1H), 6.52 (s, 2H), 4.60 (d, *J* = 10.9 Hz, 1H), 4.51 (d, *J* = 10.9 Hz, 1H), 4.10 (q, *J* = 6.7 Hz, 2H), 3.62 (s, 6H), 1.29 (t, *J* = 6.7 Hz, 3H) ppm; ^13^C-NMR (125 MHz, DMSO-d_6_): δ = 15.4, 55.1, 57.6, 74.8, 97.4, 105.9, 108.2, 109.6, 115.4, 122.7, 127.2, 128.7, 129.4, 132.3, 136.9, 137.7, 143.8, 144.3, 148.1, 154.9, 155.6, 161.1, 167.8, 174.3 ppm; Found: C, 59.78; H, 4.21; N, 4.93% C_29_H_24_Cl_2_N_2_O_7_; requires: C, 59.70; H, 4.15; N, 4.80%.

Ethyl 1'-benzyl-4-((3,5-dimethylphenyl)amino)-5',7'-dimethyl-2',5-dioxo-5H-spiro[furan-2,3'-indoline]-3-carboxylate **(Table 3, 29a):** Solid powder, m.p.: 255-257°C; ^1^H-NMR (500 MHz, DMSO-d_6_): δ = 8.48 (s, 1H, NH), 7.29 (d, *J* = 7.8 Hz, 2H), 7.23 (t, *J* = 7.8 Hz, 1H), 7.16 (t, *J* = 7.8 Hz, 2H), 7.11 (s, 1H), 6.98 (s, 1H), 6.92 (s, 1H), 6.76 (s, 2H), 4.58 (d, *J* = 10.7 Hz, 1H), 4.47 (d, *J* = 10.7 Hz, 1H), 4.09 (q, *J* = 6.8 Hz, 2H), 2.37 (s, 3H), 2.34 (s, 3H), 2.24 (s, 6H), 1.29 (t, *J* = 6.8 Hz, 3H) ppm; ^13^C-NMR (125 MHz, DMSO-d_6_): δ = 16.2, 19.9, 20.3, 21.6, 57.6, 75.9, 95.4, 108.9, 112.8, 114.2, 115.7, 120.9, 127.2, 128.7, 128.9, 129.1, 129.4, 130.4, 136.1, 137.1, 137.5, 150.5, 154.7, 163.5, 165.7, 172.4 ppm; Found: C, 73.06; H, 6.02; N, 5.58% C_31_H_30_N_2_O_5_; requires: C, 72.92; H, 5.92; N, 5.49%.

Ethyl 1'-benzyl-4-((3,4-dimethylphenyl)amino)-5',7'-dimethyl-2',5-dioxo-5H-spiro[furan-2,3'-indoline]-3-carboxylate **(Table 3, 30a):** Solid powder, m.p.: 255-257°C; ^1^H-NMR (500 MHz, DMSO-d_6_): δ = 8.34 (s, 1H, NH), 7.30 (d, *J* = 7.8 Hz, 2H), 7.23 (t, *J* = 7.8 Hz, 1H), 7.18 (t, *J* = 7.8 Hz, 2H), 7.10 (s, 1H), 7.08 (d, *J* = 7.8 Hz, 1H), 7.01 (s, 1H), 6.98 (d, *J* = 7.8 Hz, 1H), 6.77 (s, 1H), 4.55 (d, *J* = 10.8 Hz, 1H), 4.45 (d, *J* = 10.8 Hz, 1H), 4.07 (q, *J* = 6.8 Hz, 2H), 2.36 (s, 3H), 2.32 (s, 3H), 2.25 (s, 3H), 2.19 (s, 3H), 1.28 (t, *J* = 6.8 Hz, 3H) ppm; ^13^C-NMR (125 MHz, DMSO-d_6_): δ = 16.4, 19.8, 20.1, 20.4, 21.9, 58.1, 76.4, 96.4, 108.8, 112.7, 115.7, 119.2, 122.3, 125.4, 127.2, 128.6, 128.9, 129.3, 129.5, 130.8, 136.3, 136.7, 137.1, 137.6, 150.1, 154.6, 163.1, 165.7, 172.7 ppm; Found: C, 73.01; H, 5.85; N, 5.43% C_31_H_30_N_2_O_5_; requires: C, 72.92; H, 5.92; N, 5.49%.

Ethyl 1'-benzyl-4-((3,5-dimethoxyphenyl)amino)-5',7'-dimethyl-2',5-dioxo-5H-spiro[furan-2,3'-indoline]-3-carboxylate **(Table 3, 31a):** Solid powder, m.p.: 259-261°C; ^1^H-NMR (500 MHz, DMSO-d_6_): δ = 8.22 (s, 1H, NH), 7.29 (d, *J* = 7.8 Hz, 2H), 7.24 (t, *J* = 7.7 Hz, 1H), 7.17 (t, *J* = 7.7 Hz, 2H), 7.10 (s, 1H), 6.97 (s, 1H), 6.66 (s, 1H), 6.49 (s, 2H), 4.56 (d, *J* = 11.00 Hz, 1H), 4.47 (d, *J* = 11.00 Hz, 1H), 4.08 (q, *J* = 6.9 Hz, 2H), 3.89 (s, 6H), 2.37 (s, 3H), 2.33 (s, 3H), 1.29 (t, *J* = 6.9 Hz, 3H) ppm; ^13^C-NMR (125 MHz, DMSO-d_6_): δ = 16.0, 20.4, 21.7, 55.1, 57.9, 76.2, 98.1, 105.4, 108.6, 109.8, 115.4, 121.2, 127.1, 128.6, 128.9, 129.0, 129.4, 130.7, 137.0, 137.6, 147.5, 154.1, 154.7, 161.2, 165.8, 173.4 ppm; Found: C, 68.71; H, 5.69; N, 5.23% C_31_H_30_N_2_O_7_; requires: C, 68.62; H, 5.57; N, 5.16%; MASS(M/Z): 563 M+Na), 496 (100%).


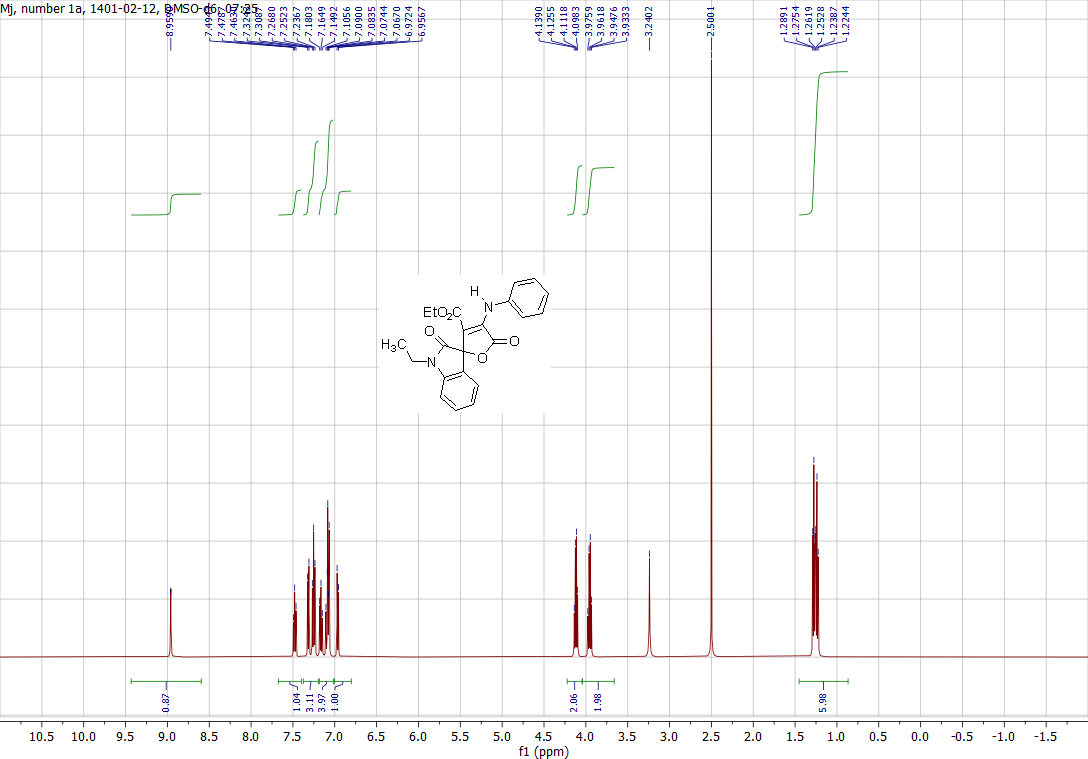


**Figure S_22_:** ^1^H-NMR spectrum of **12a**


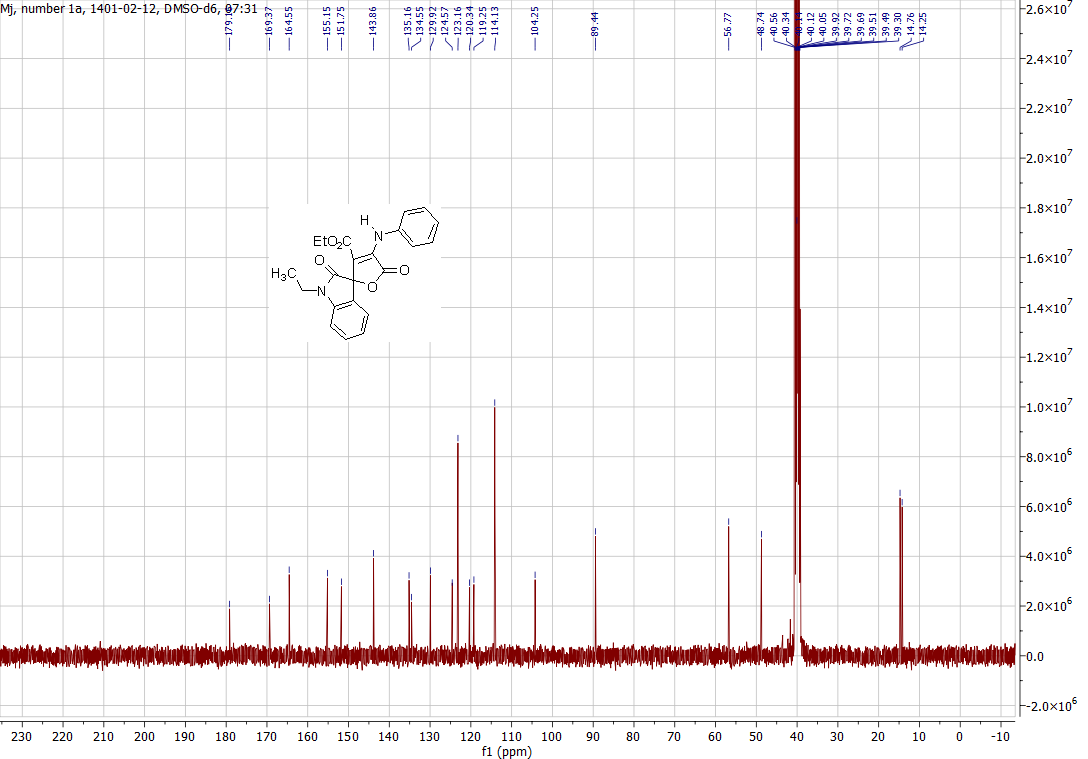


**Figure S_23_:** ^13^C-NMR spectrum of **12a**


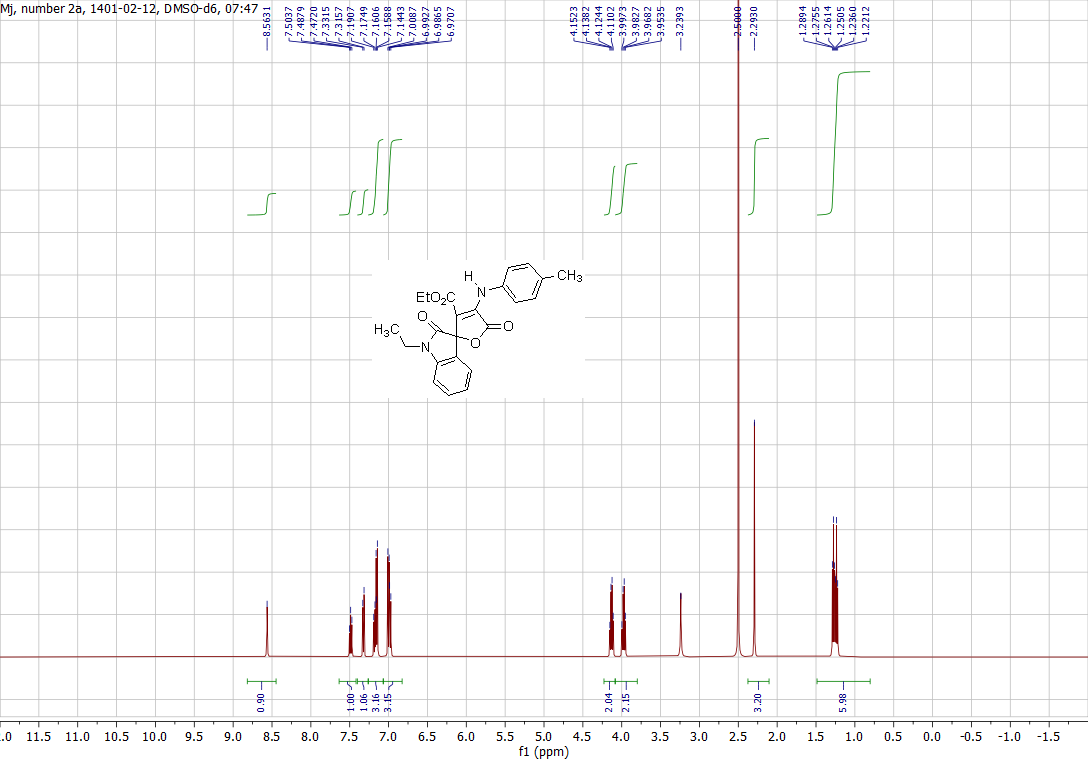


**Figure S_24_:** ^1^H-NMR spectrum of **13a**


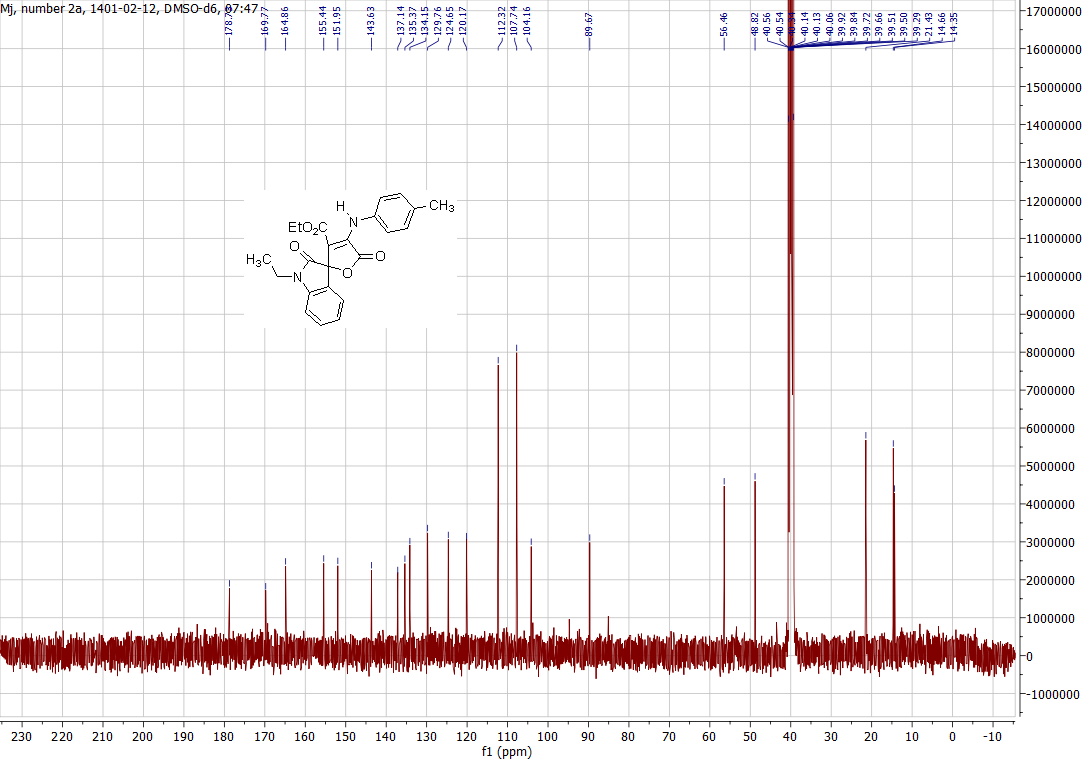


**Figure S_25_:** ^13^C-NMR spectrum of **13a**


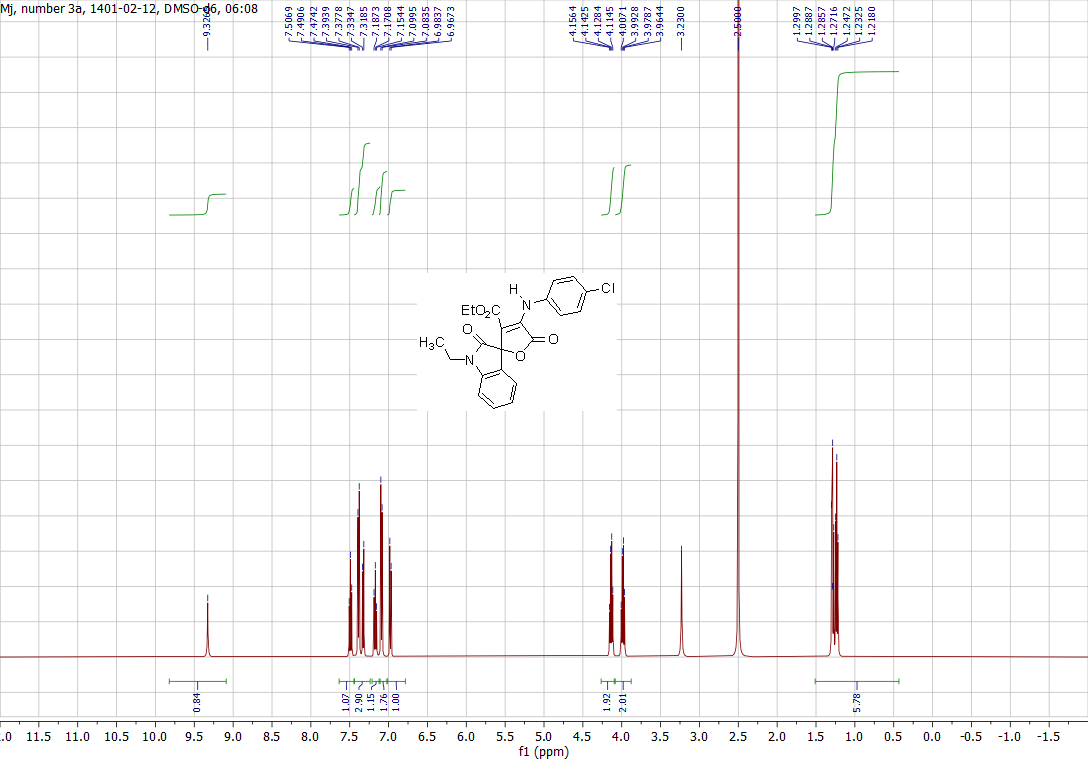


**Figure S_26_:** ^1^H-NMR spectrum of **14a**


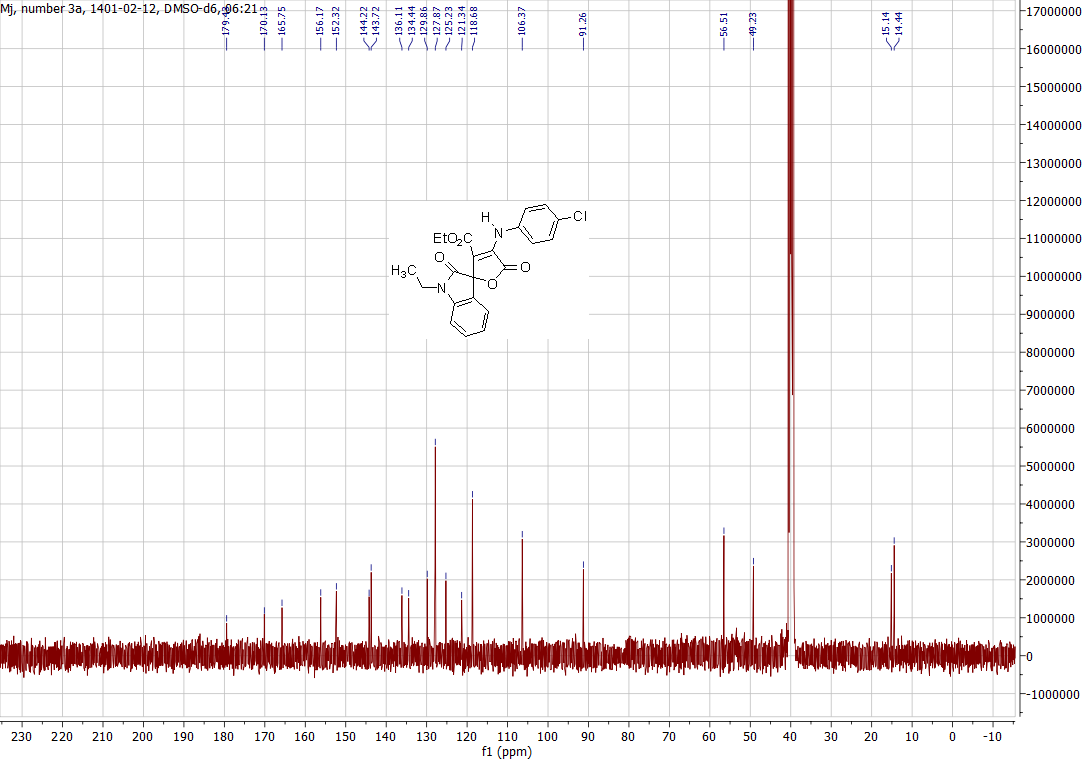


**Figure S_27_:** ^13^C-NMR spectrum of **14a**


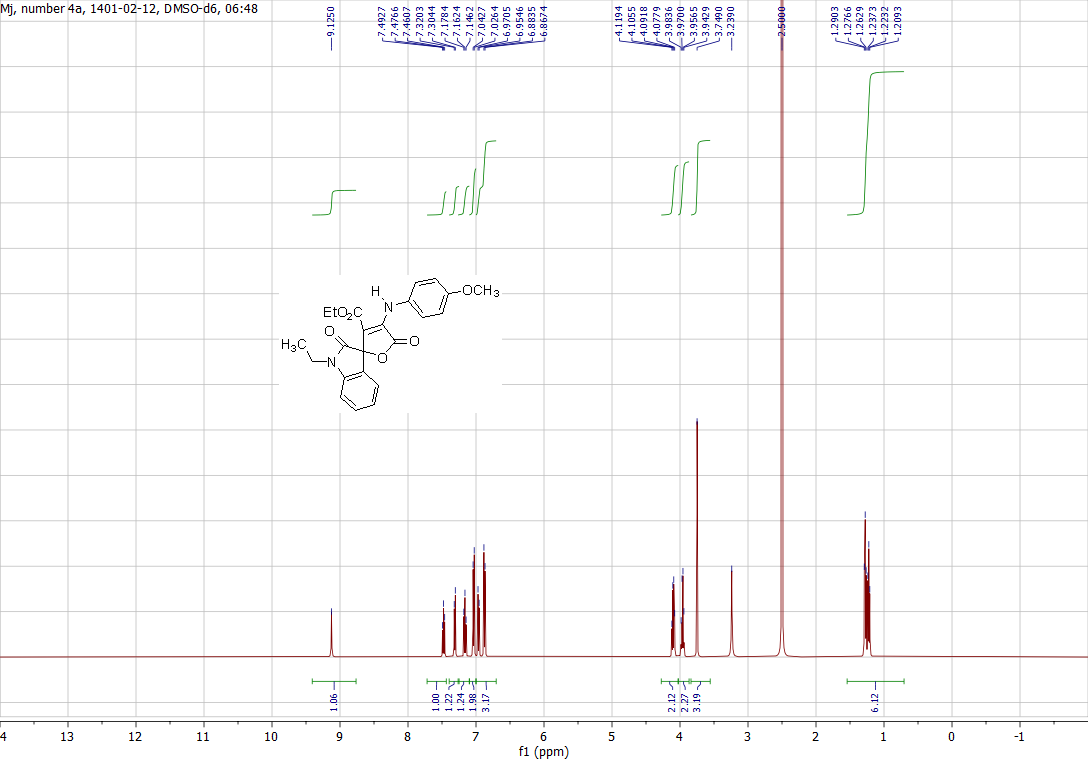


**Figure S_28_:** ^1^H-NMR spectrum of **15a**


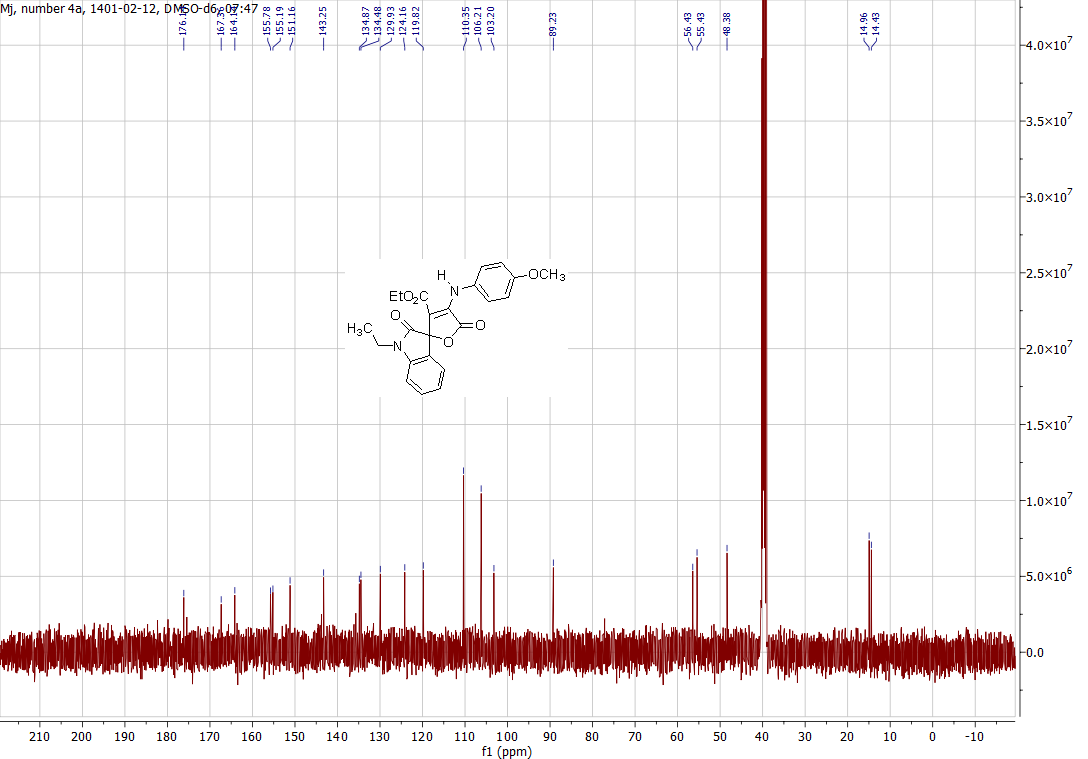


**Figure S_29_:** ^13^C-NMR spectrum of **15a**


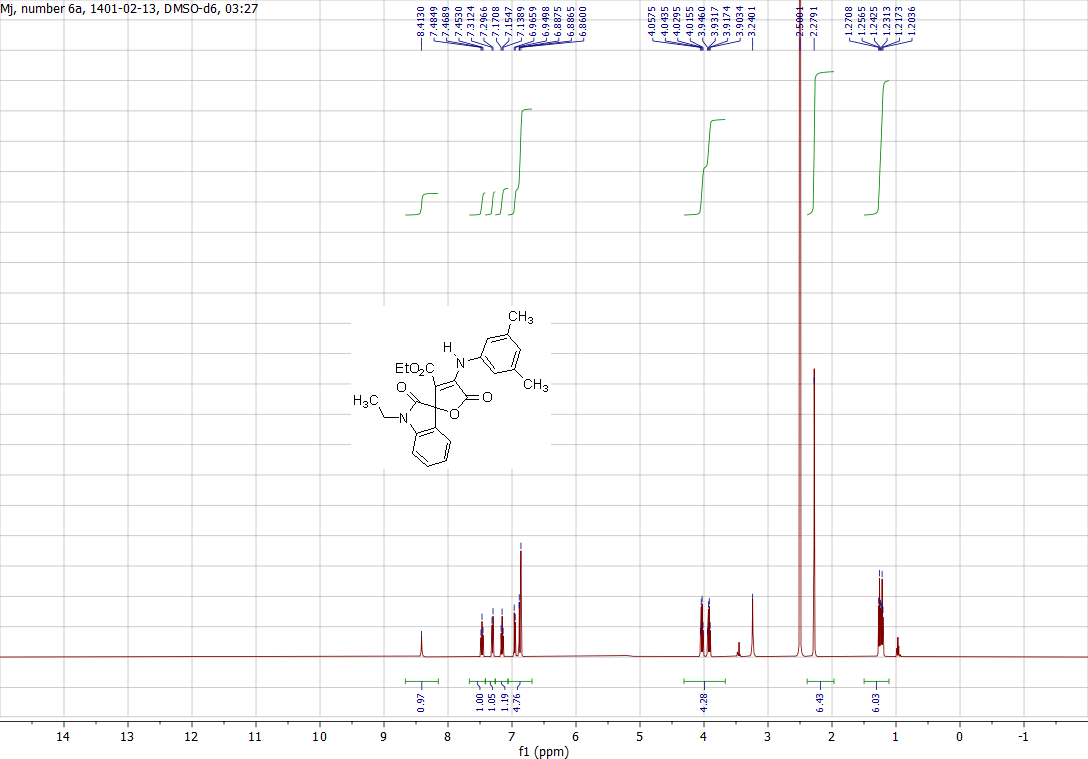


**Figure S_30_:** ^1^H-NMR spectrum of **16a**


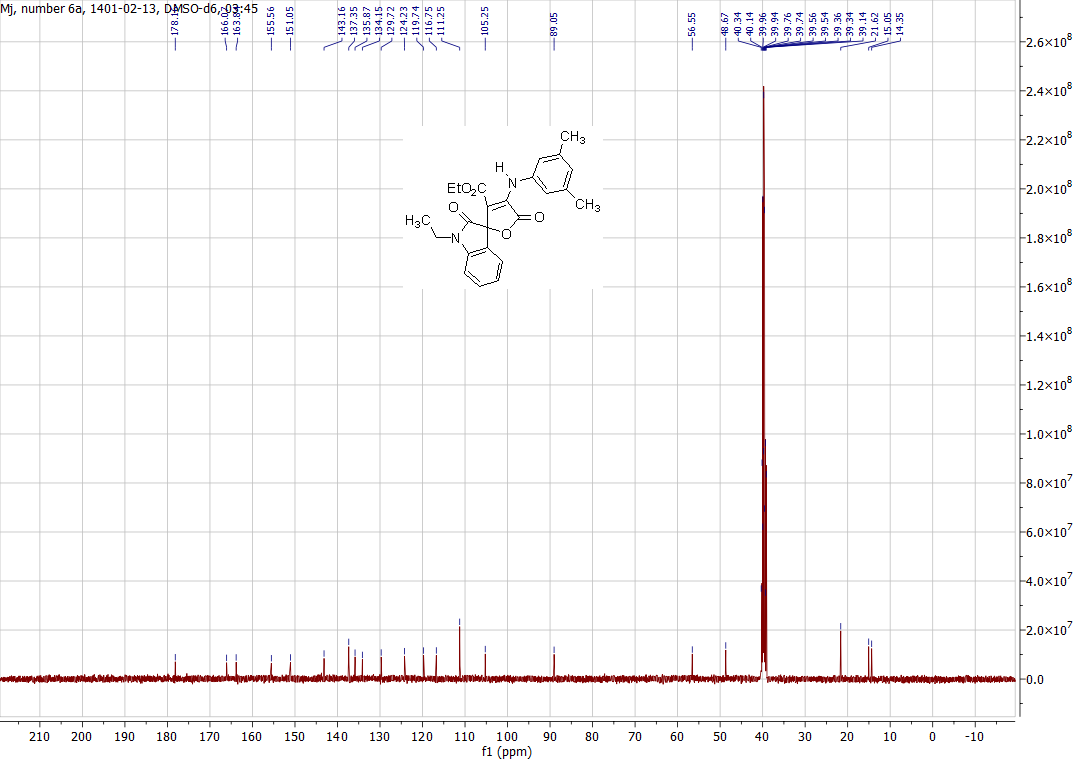


**Figure S_31_:** ^13^C-NMR spectrum of **16a**


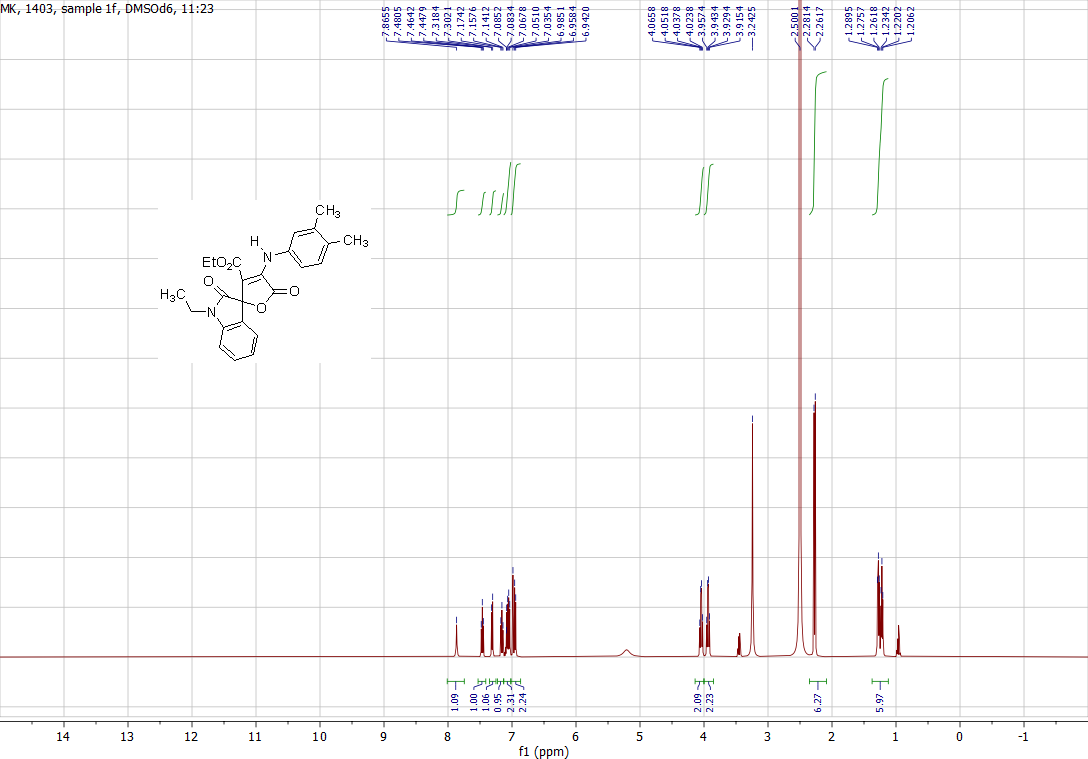


**Figure S_32_:** ^1^H-NMR spectrum of **17a**


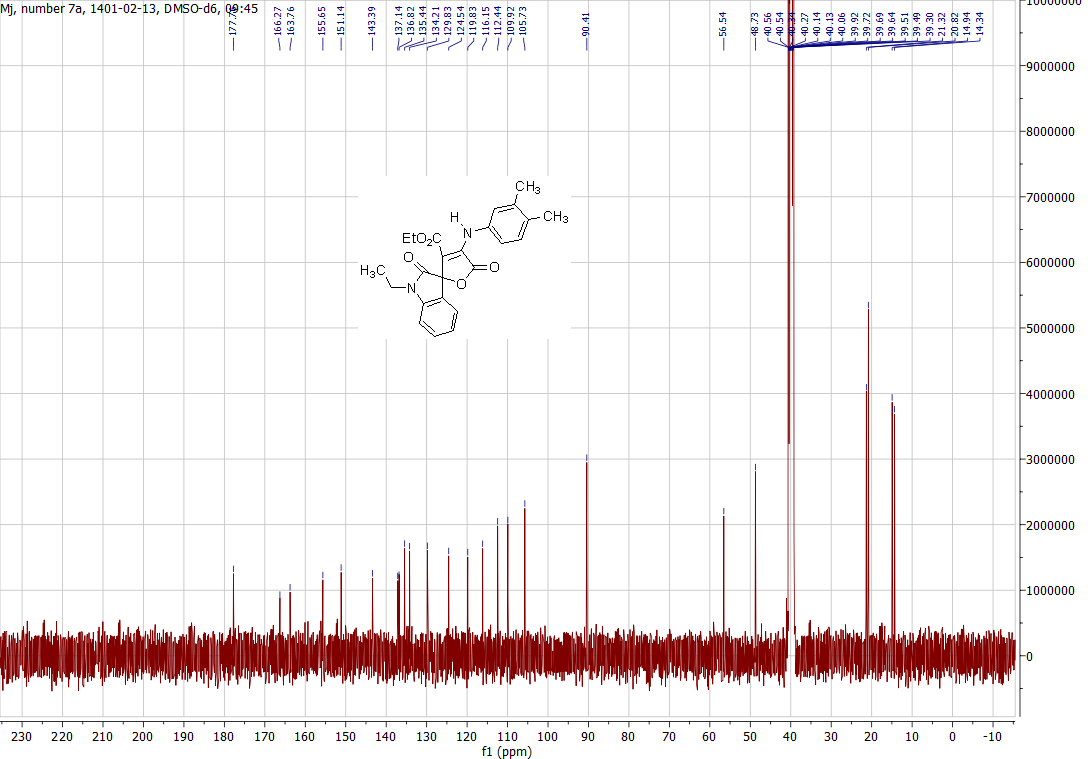


**Figure S_33_:** ^13^C-NMR spectrum of **17a**


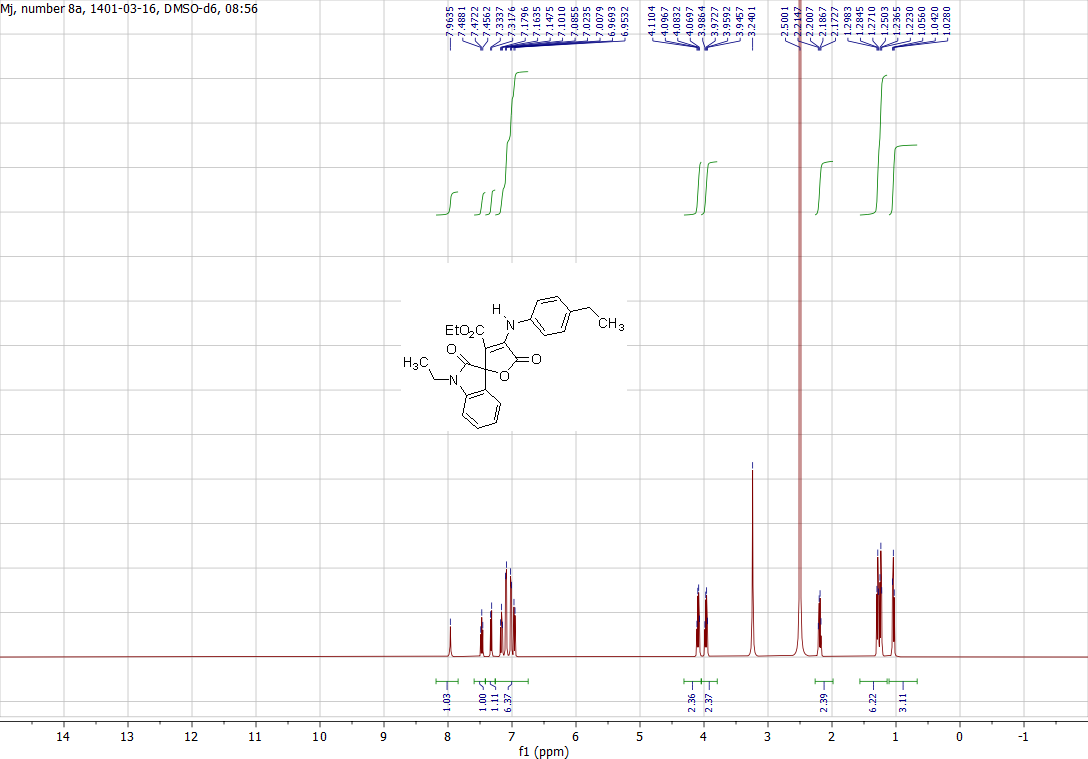


**Figure S_36_:** ^1^H-NMR spectrum of **18a**


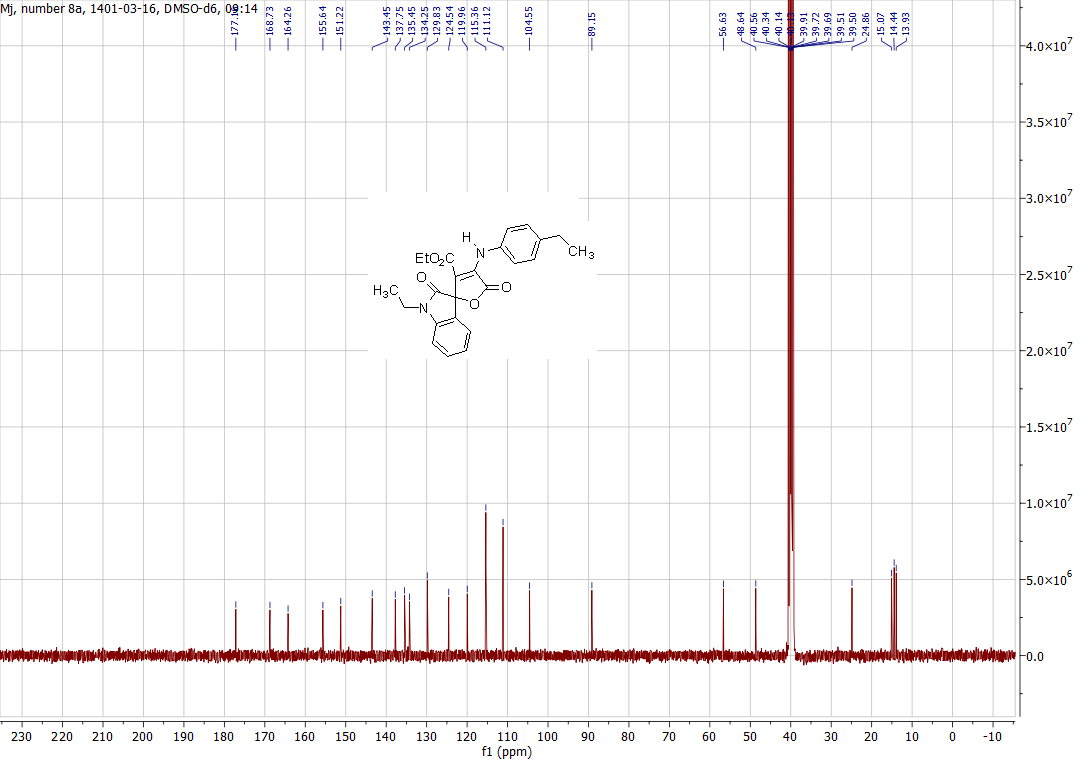


**Figure S_37_:** ^13^C-NMR spectrum of **18a**


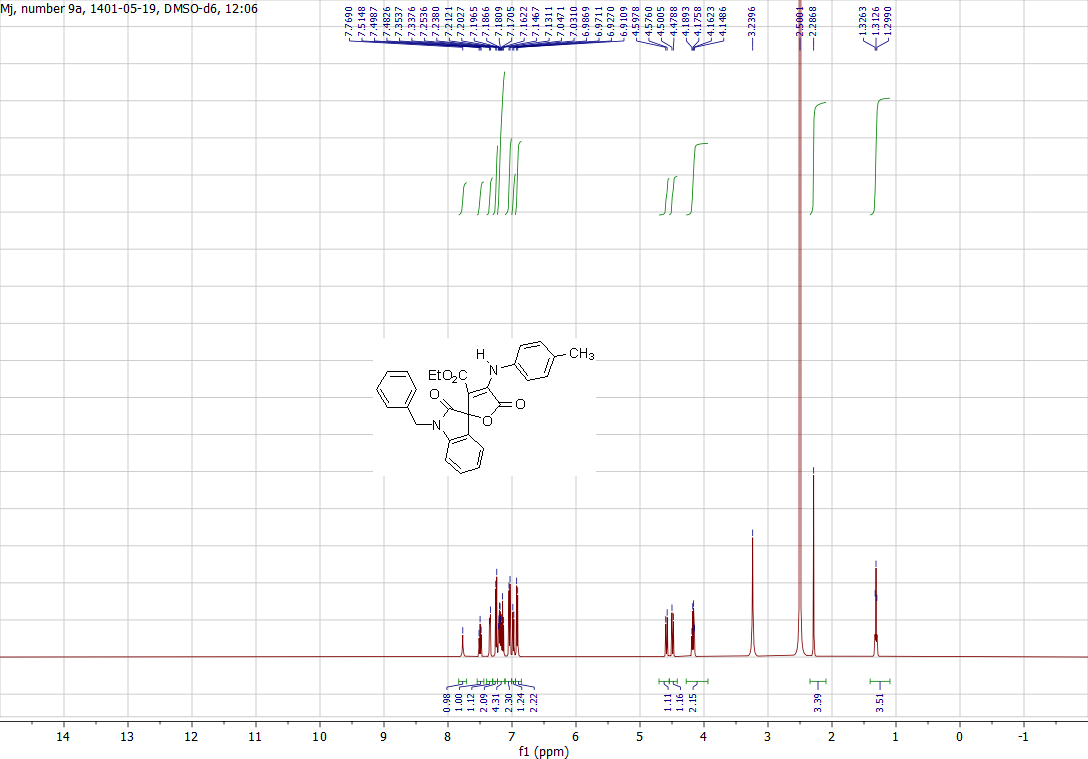


**Figure S_38_:** ^1^H-NMR spectrum of **19a**

**
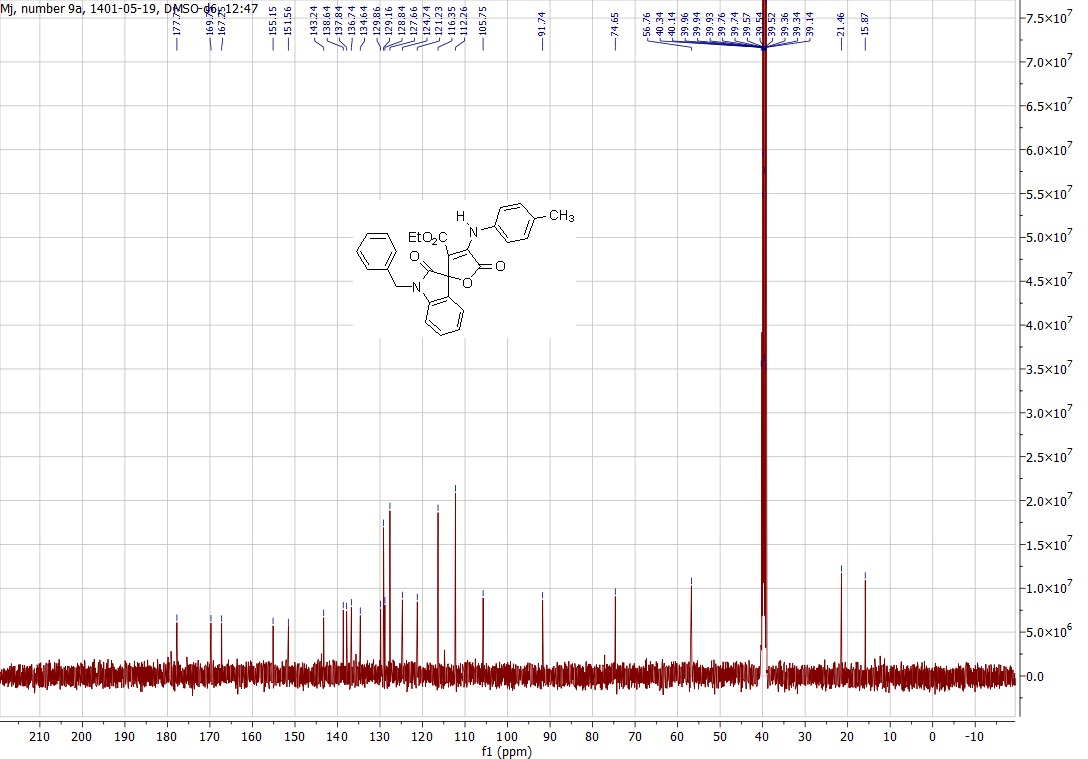
**

**Figure S_39_:** ^13^C-NMR spectrum of **19a**


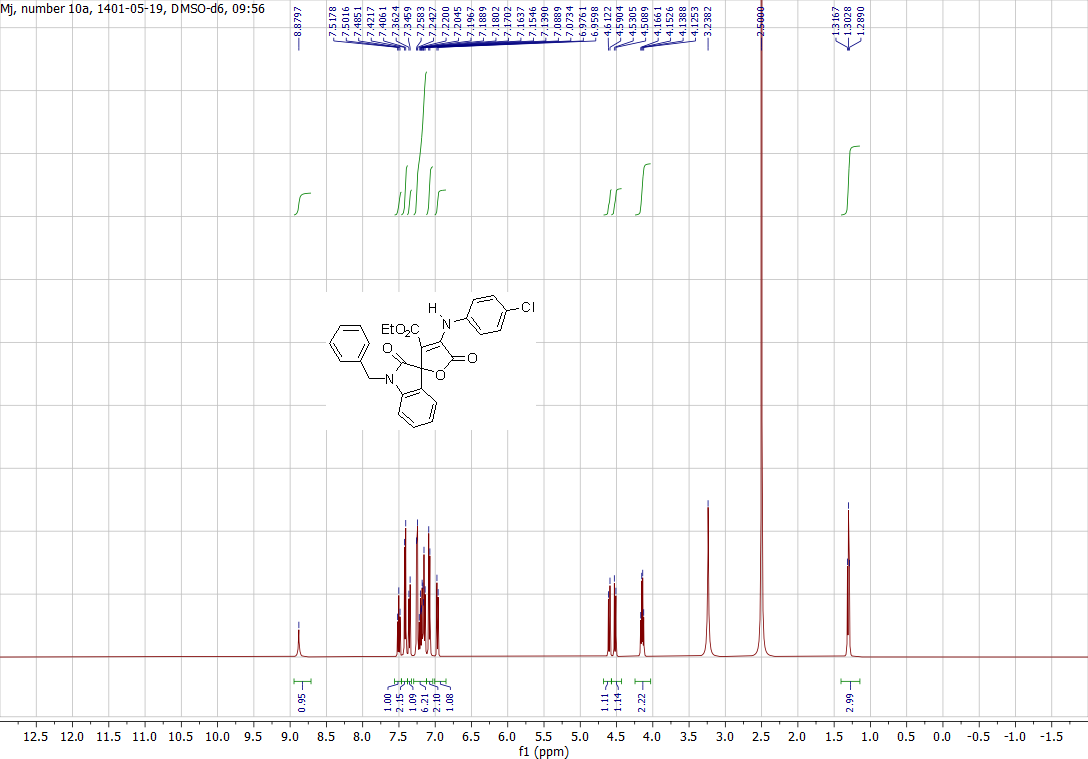


**Figure S_40_:** ^1^H-NMR spectrum of **20a**


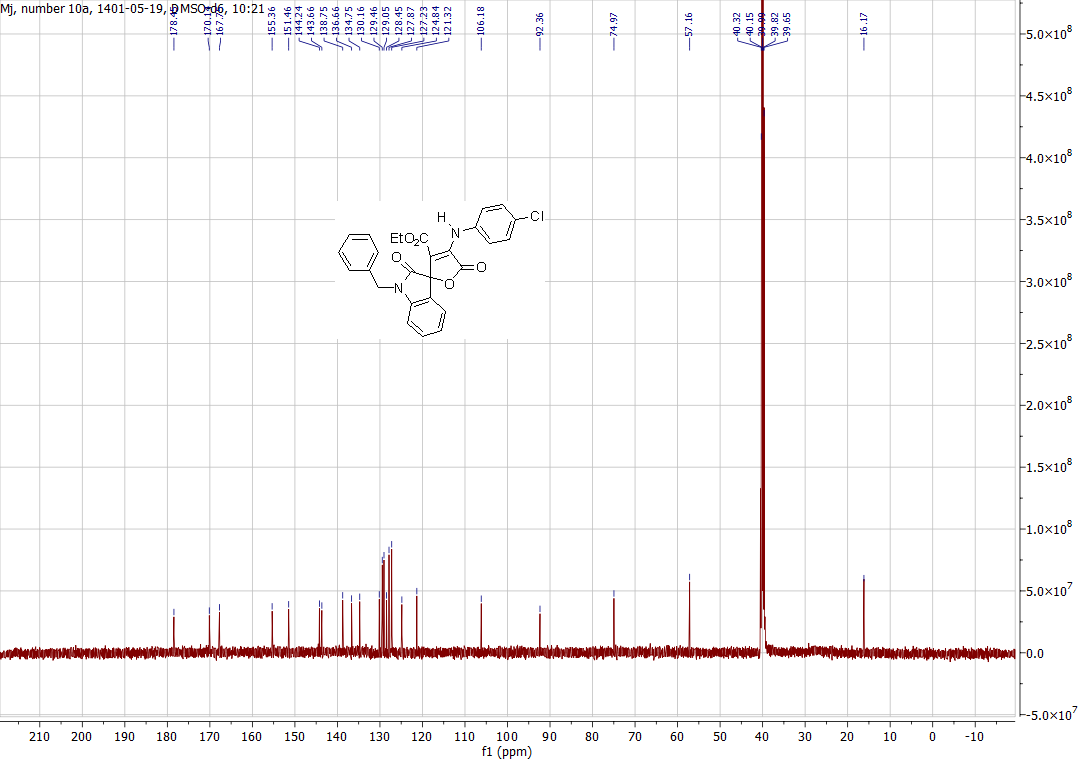


**Figure S_41_:** ^13^C-NMR spectrum of **20a**


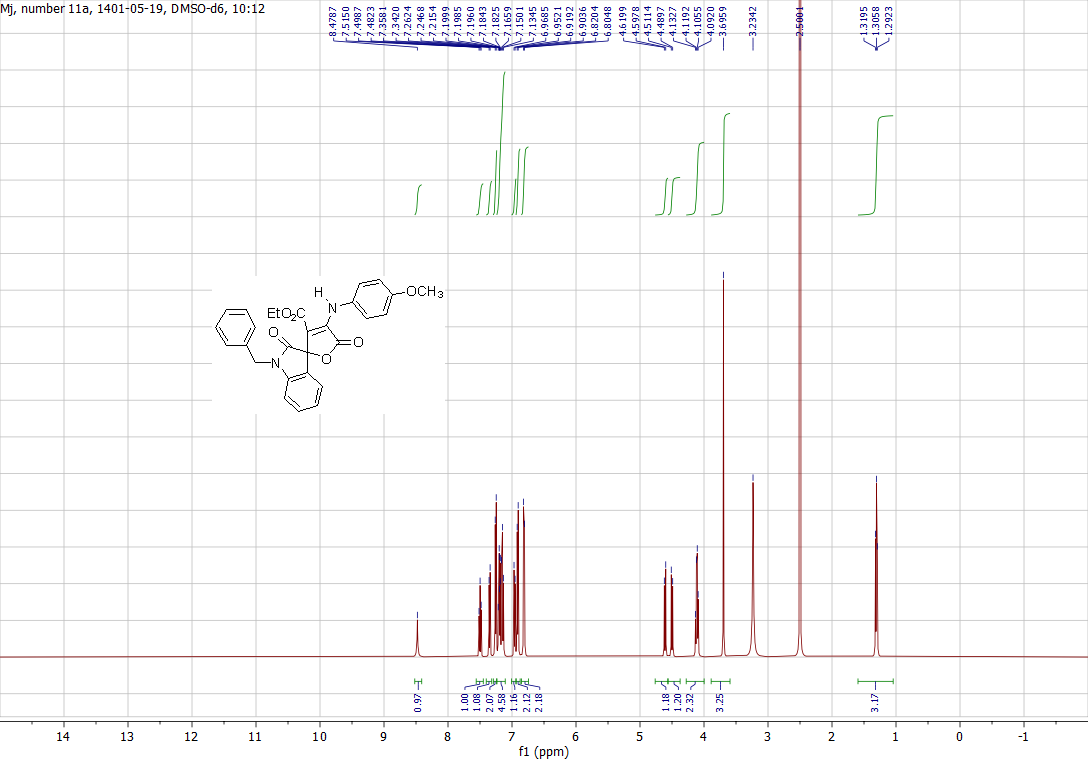


**Figure S_42_:** ^1^H-NMR spectrum of **21a**


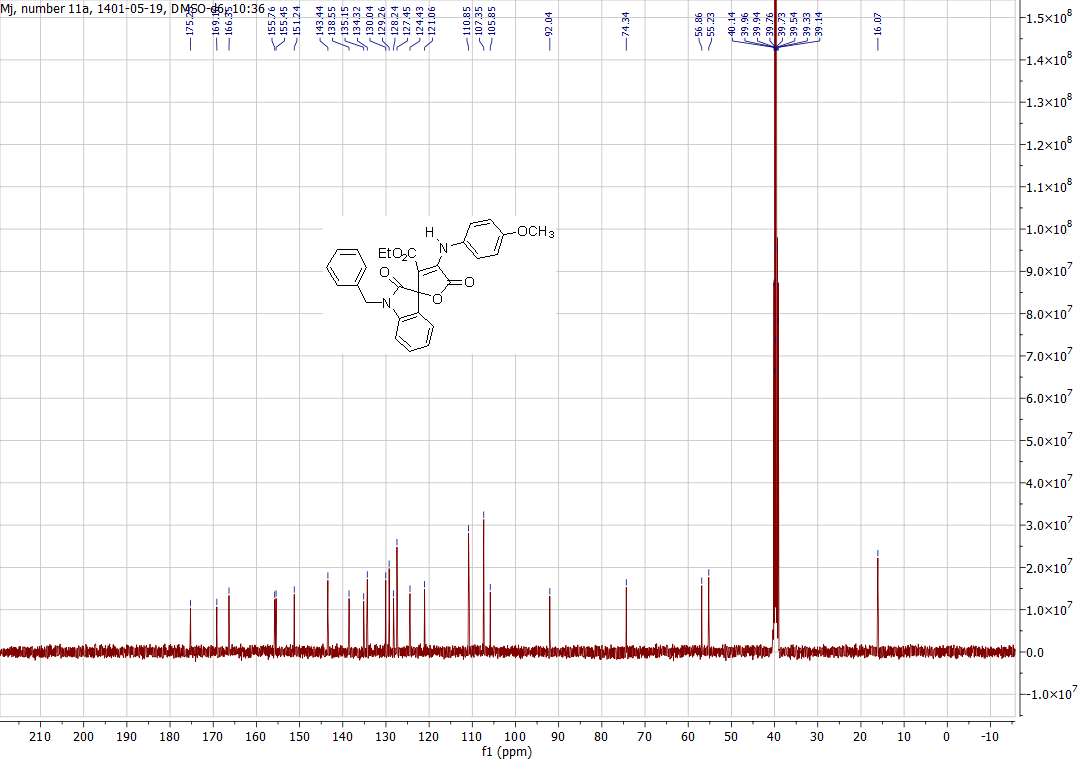


**Figure S_43_:** ^13^C-NMR spectrum of **21a**


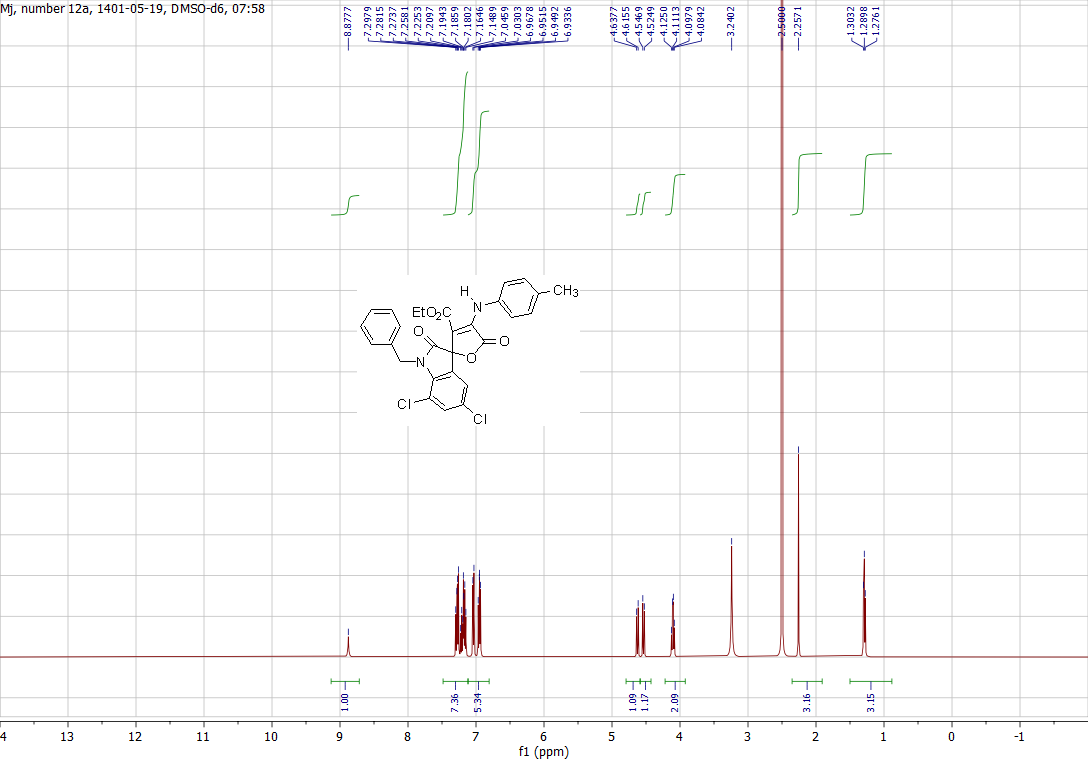


**Figure S_44_:** ^1^H-NMR spectrum of **22a**


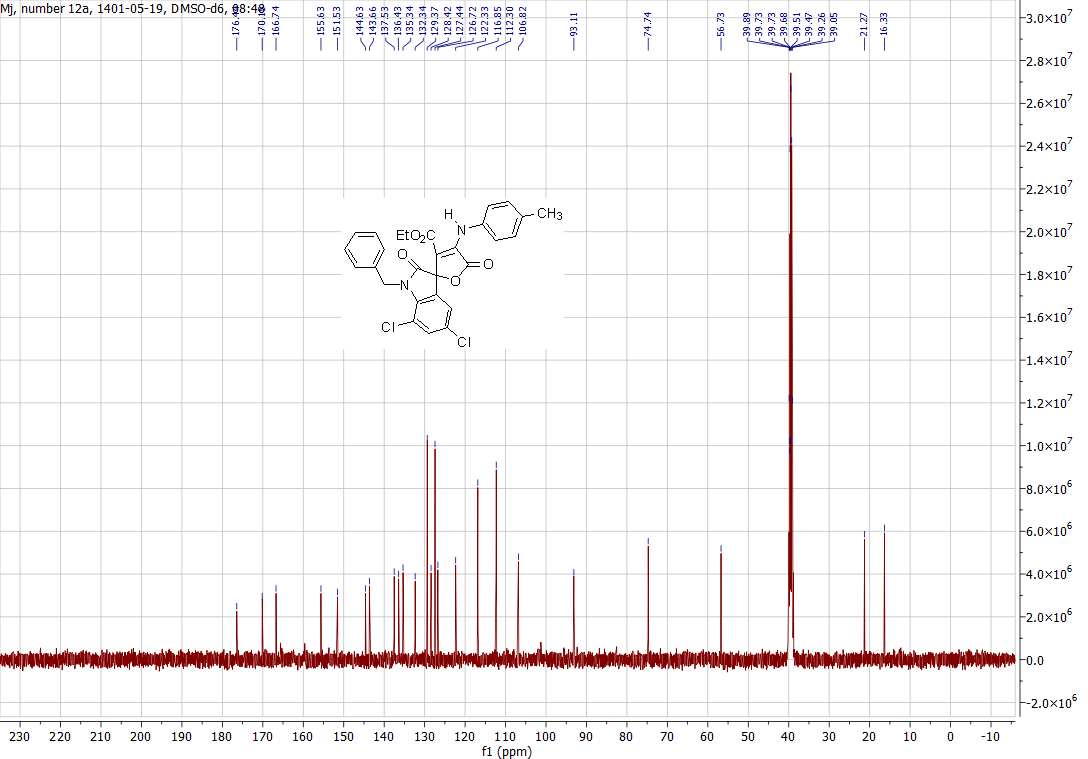


**Figure S_45_:** ^13^C-NMR spectrum of **22a**


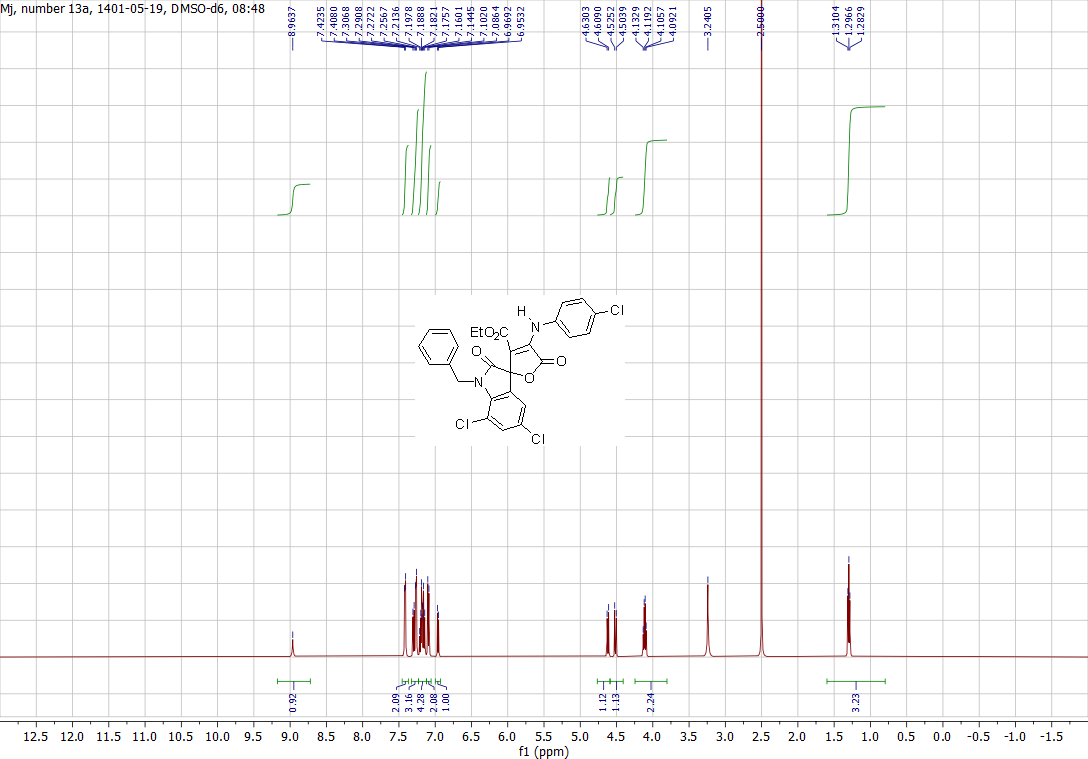


**Figure S_46_:** ^1^H-NMR spectrum of **23a**


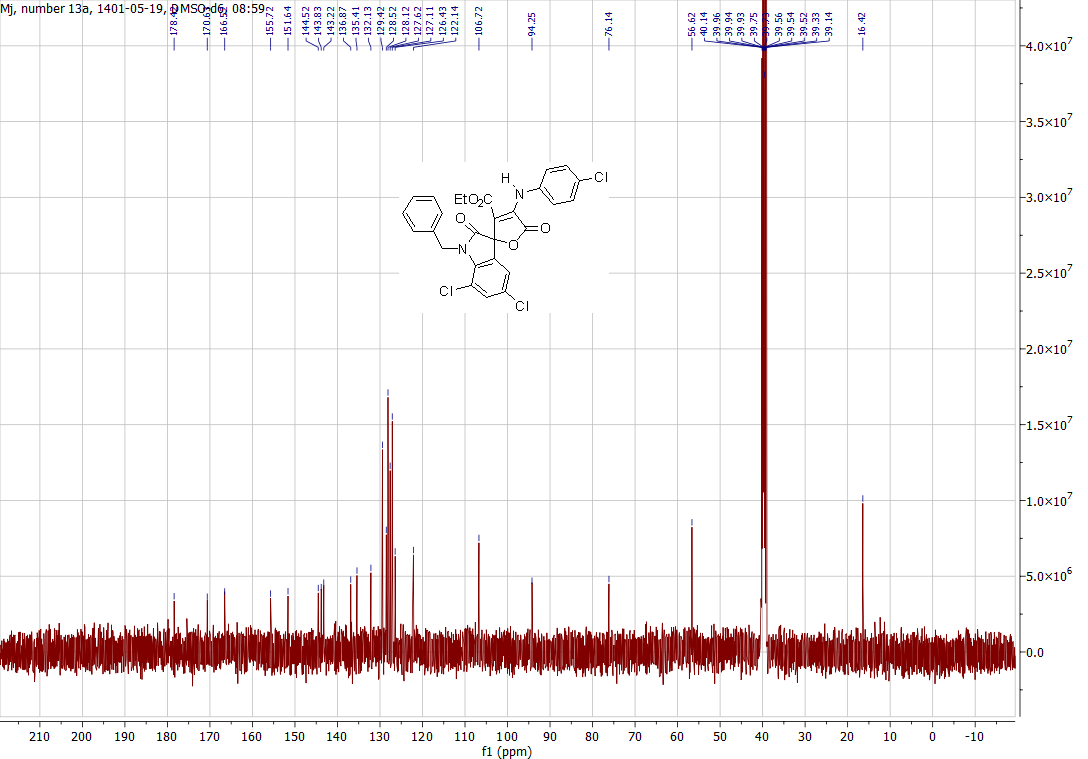


**Figure S_47_:** ^13^C-NMR spectrum of **23a**


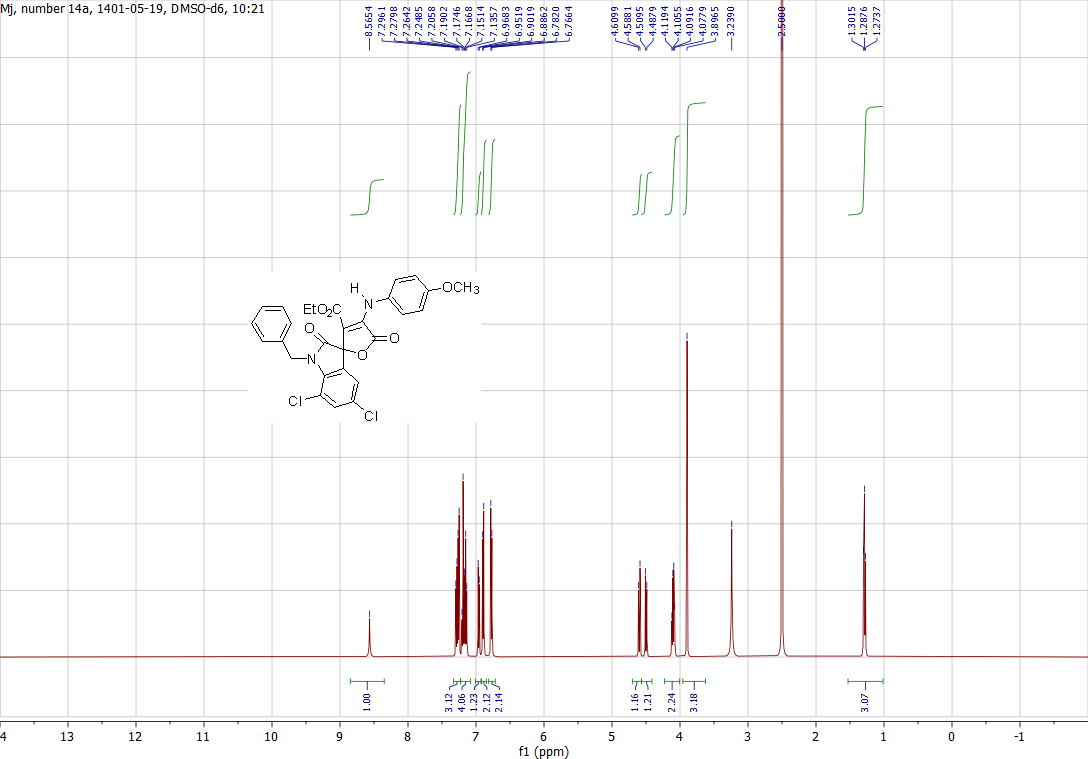


**Figure S_48_:** ^1^H-NMR spectrum of **24a**


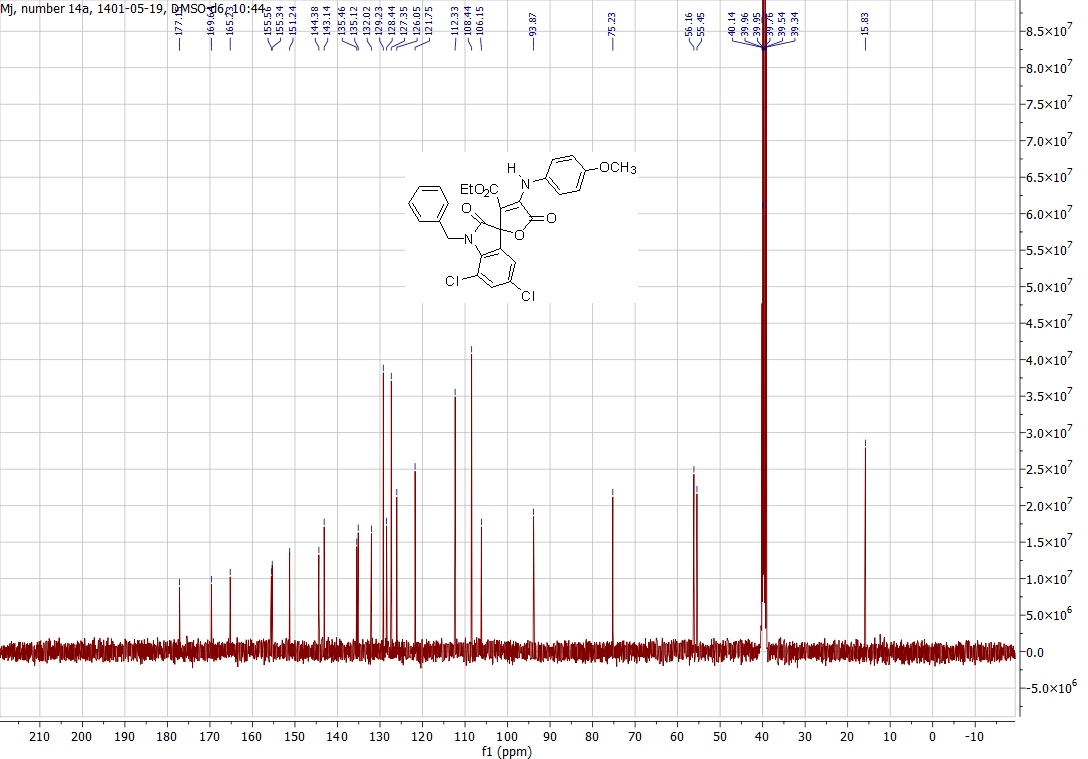


**Figure S_49_:** ^13^C-NMR spectrum of **24a**


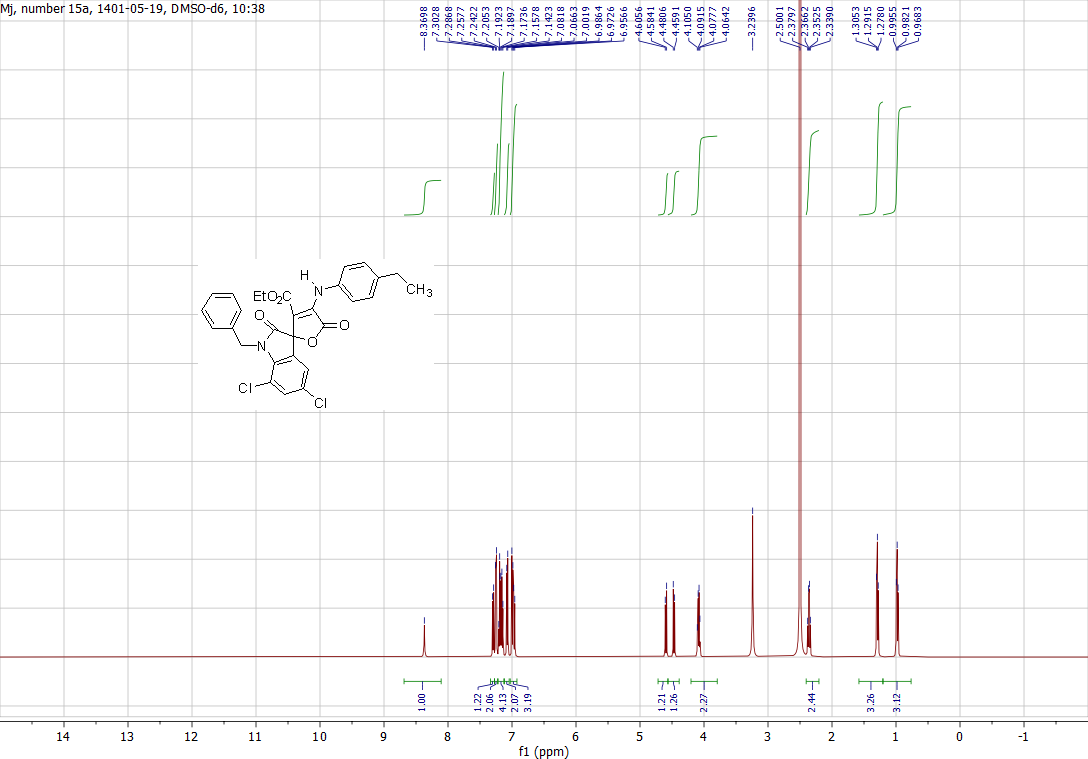


**Figure S_50_:** ^1^H-NMR spectrum of **25a**


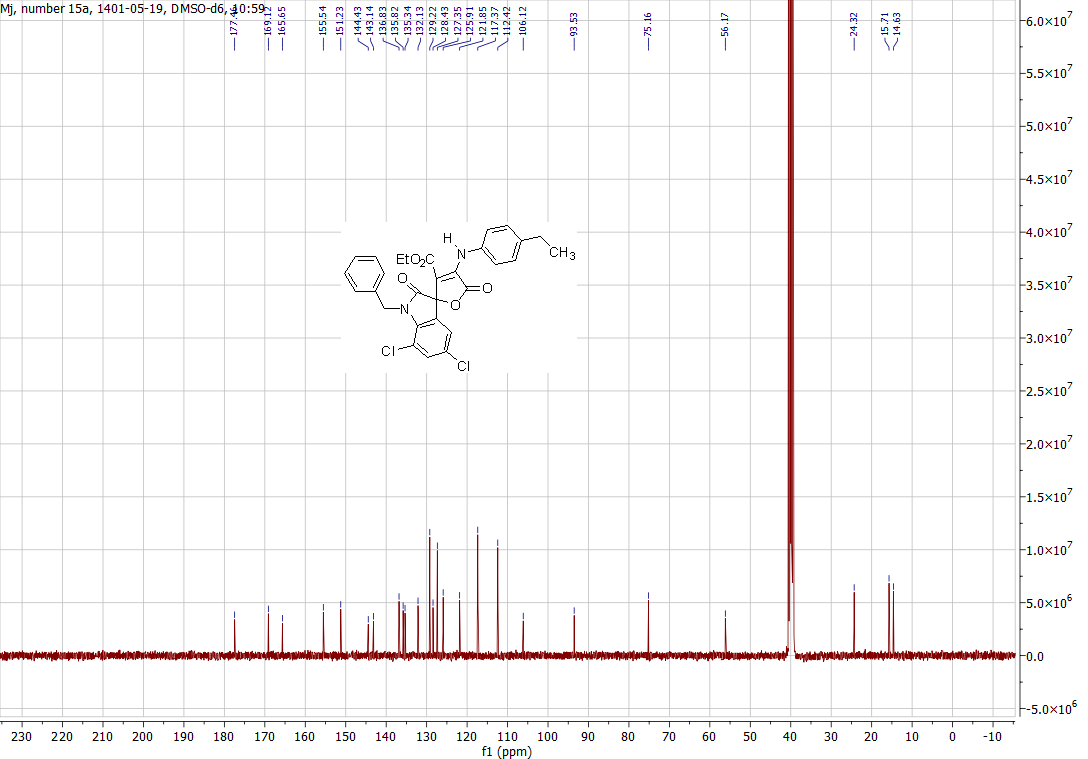


**Figure S_51_:** ^13^C-NMR spectrum of **25a**


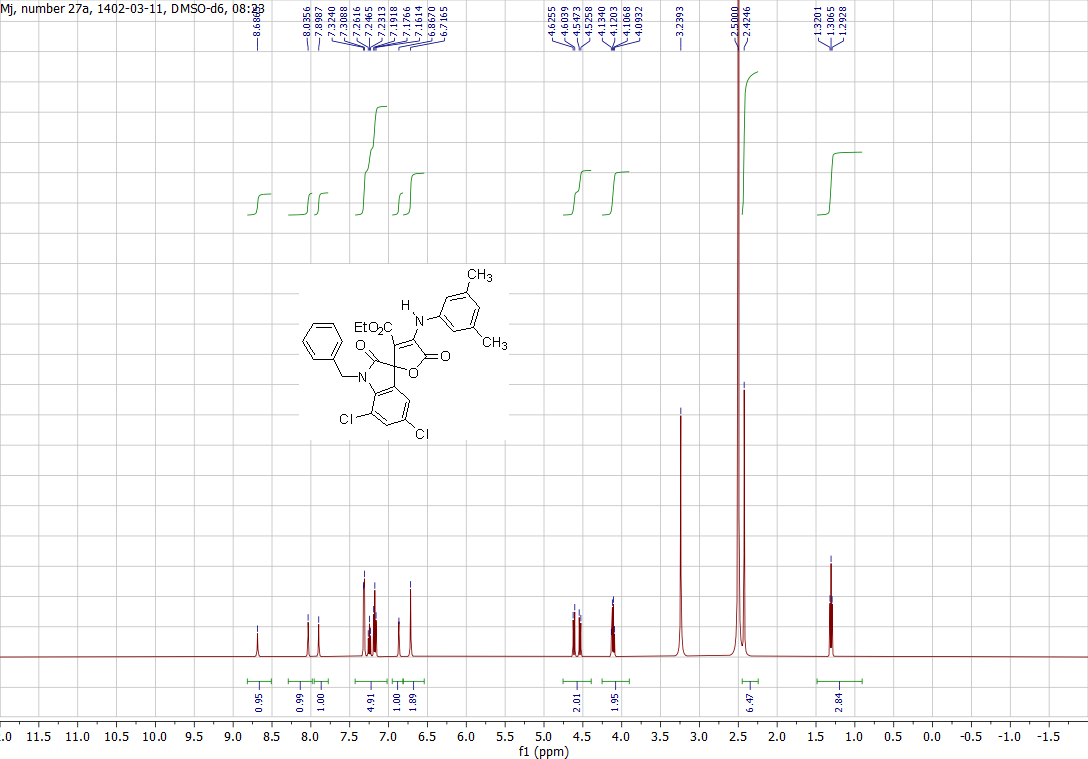


**Figure S_52_:** ^1^H-NMR spectrum of **26a**


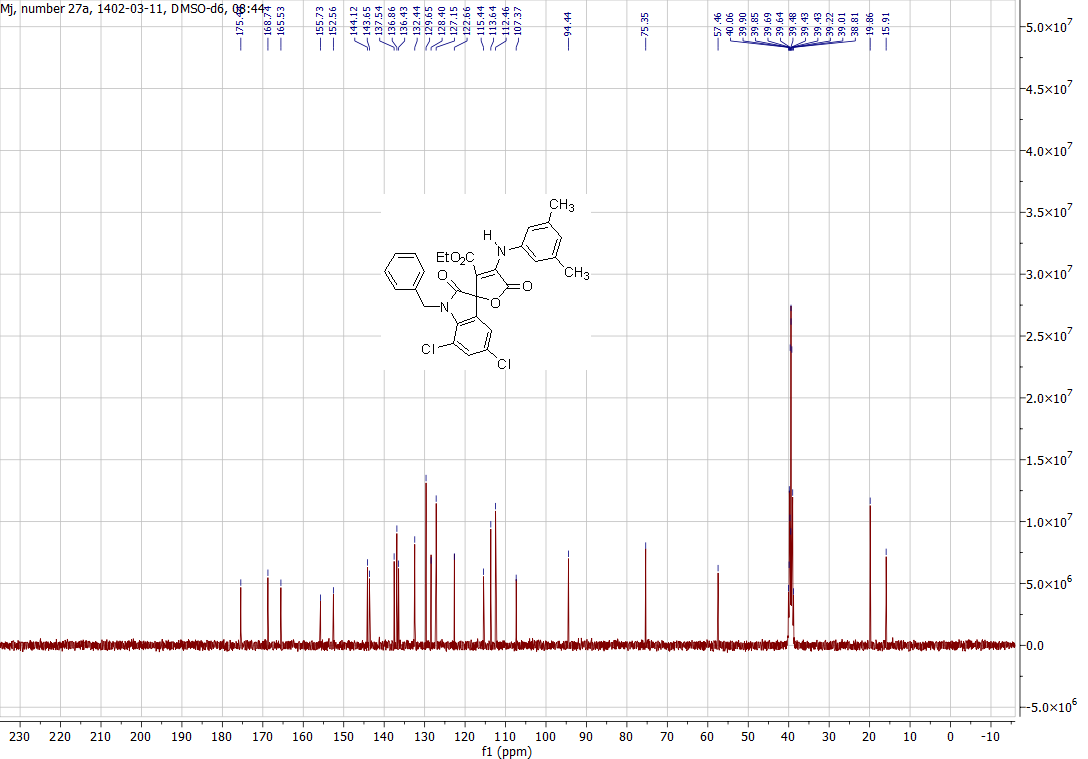


**Figure S_53_:** ^13^C-NMR spectrum of **26a**


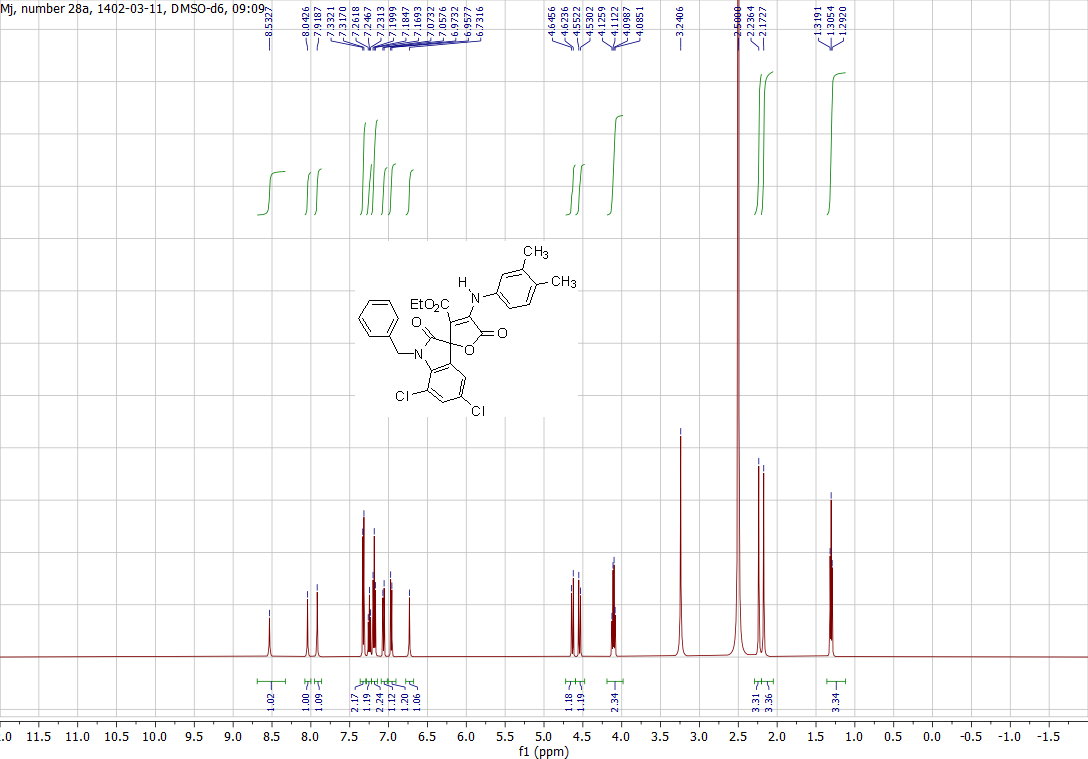


**Figure S_54_:** ^1^H-NMR spectrum of **27a**


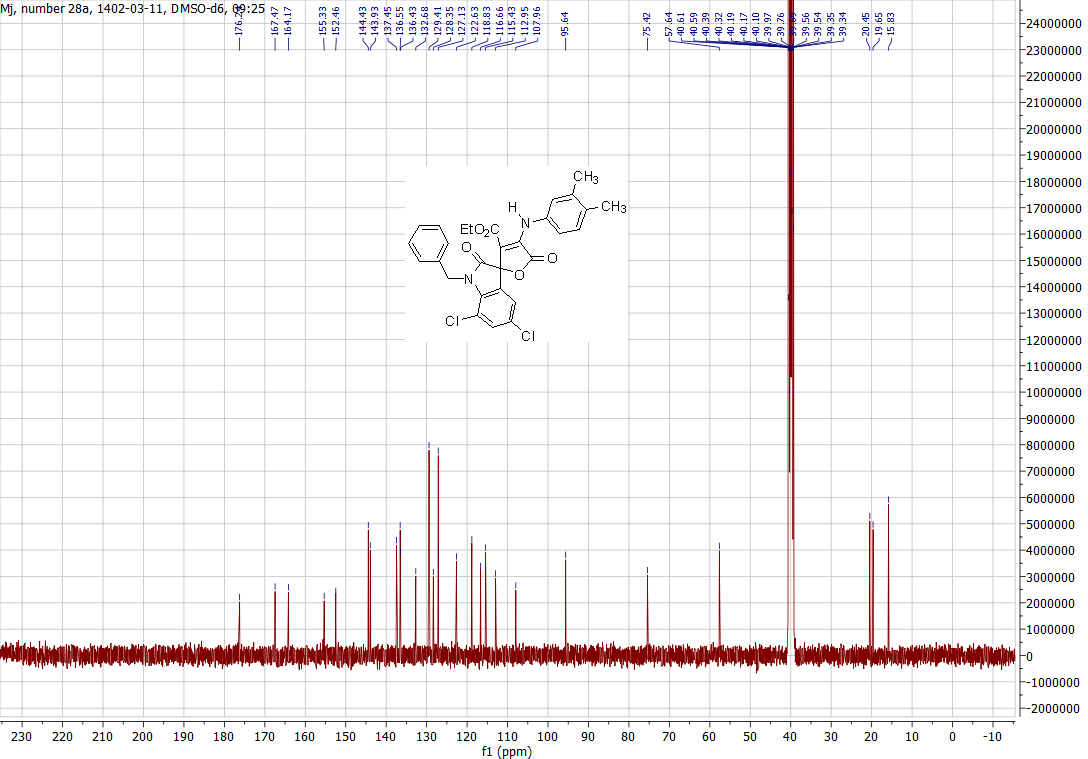


**Figure S_55_:** ^13^C-NMR spectrum of **27a**


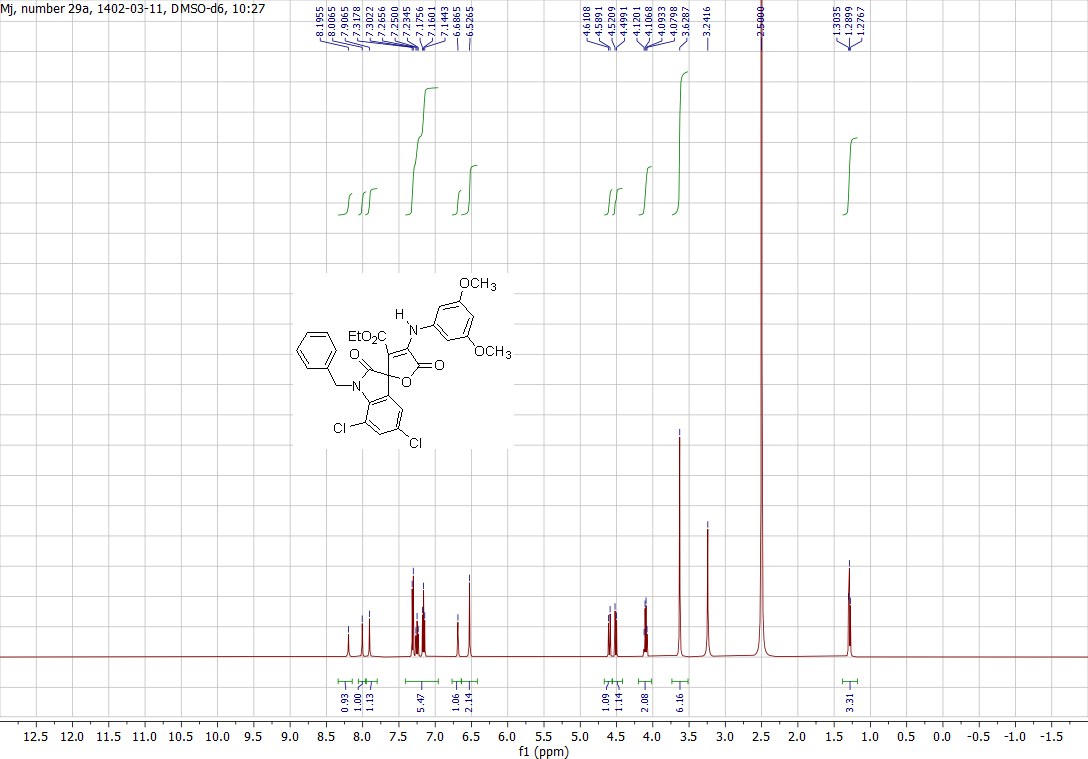


**Figure S_56_:** ^1^H-NMR spectrum of **28a**


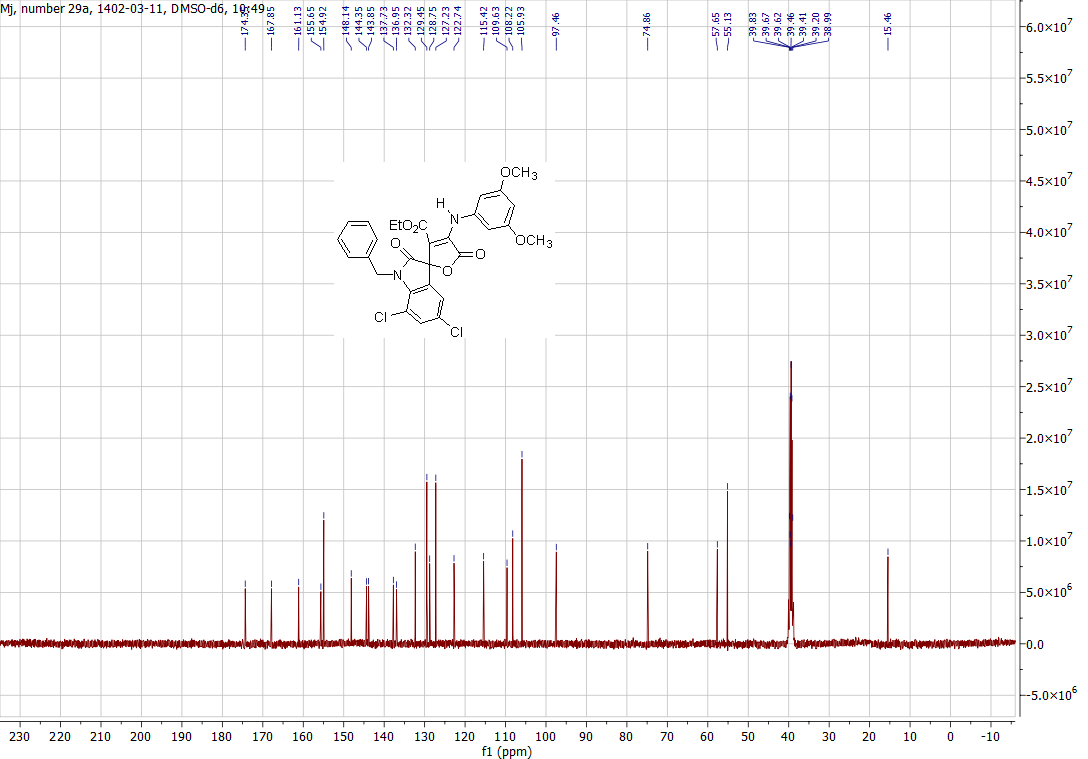


**Figure S_57_:** ^13^C-NMR spectrum of **28a**


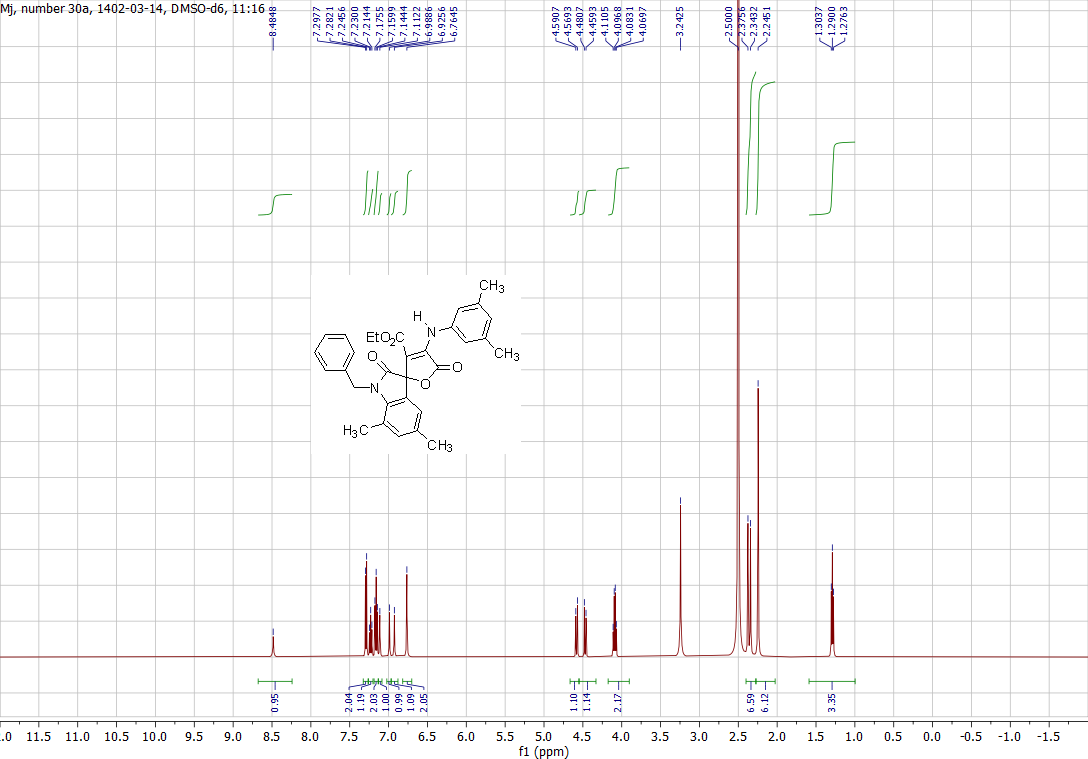


**Figure S_58_:** ^1^H-NMR spectrum of **29a**


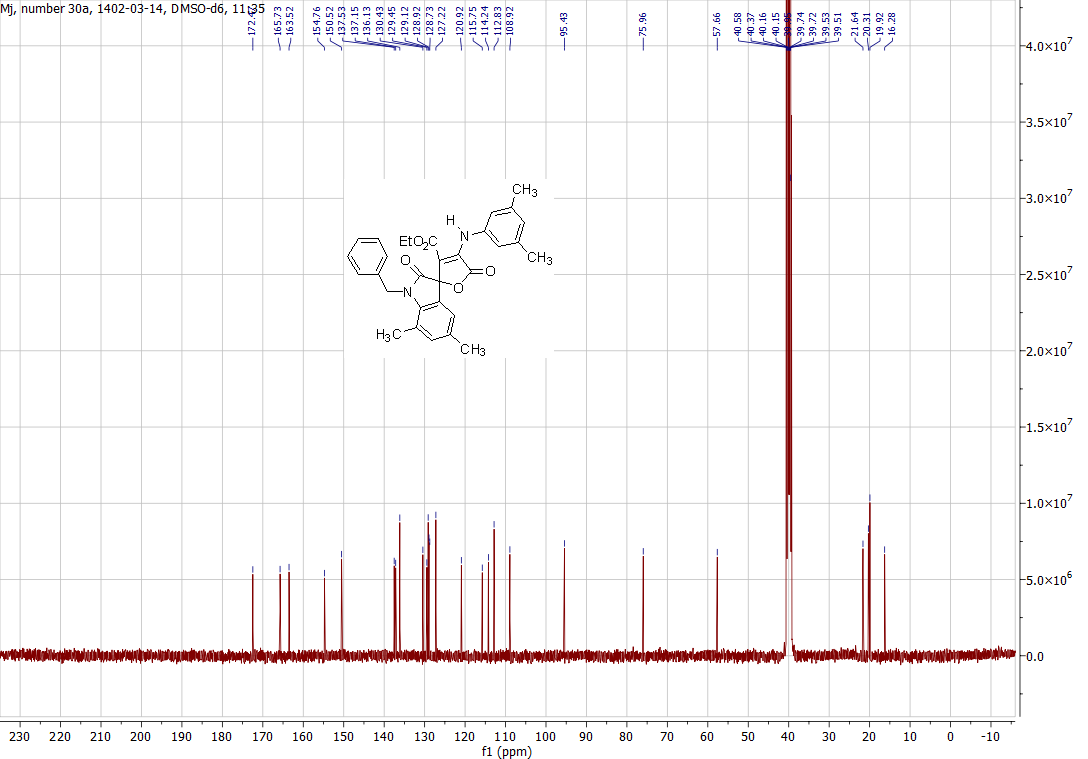


**Figure S_59_:** ^13^C-NMR spectrum of **29a**


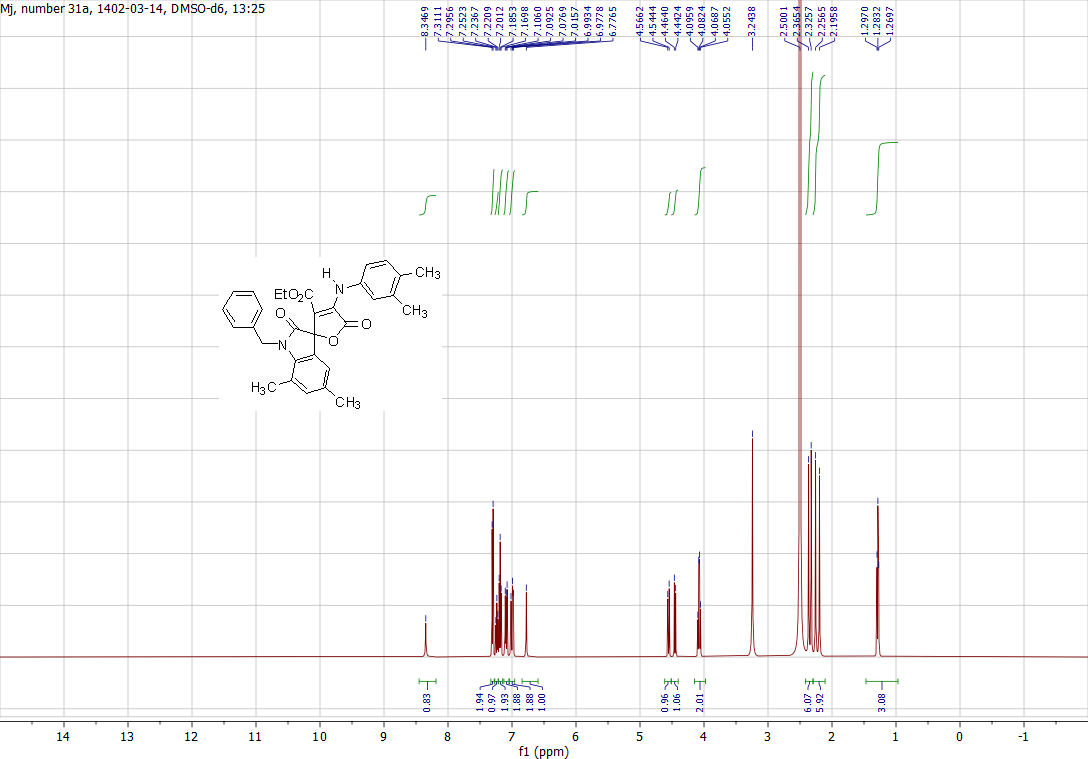


**Figure S_60_:** ^1^H-NMR spectrum of **30a**


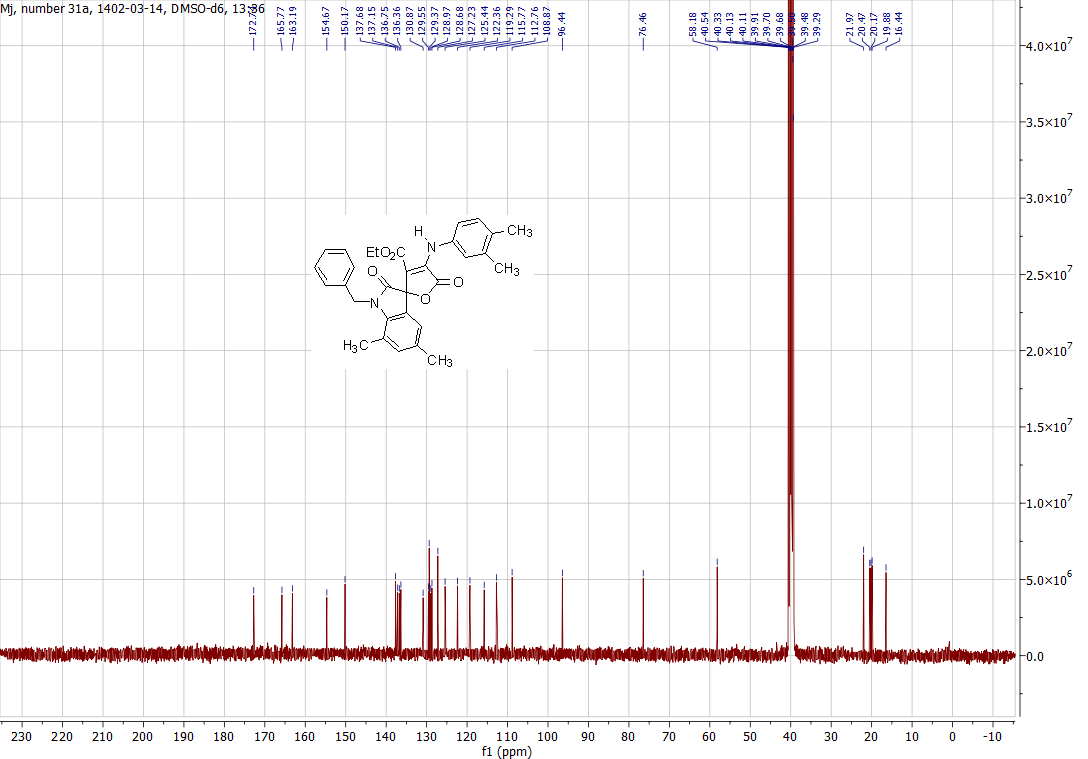


**Figure S_61_:** ^13^C-NMR spectrum of **30a**


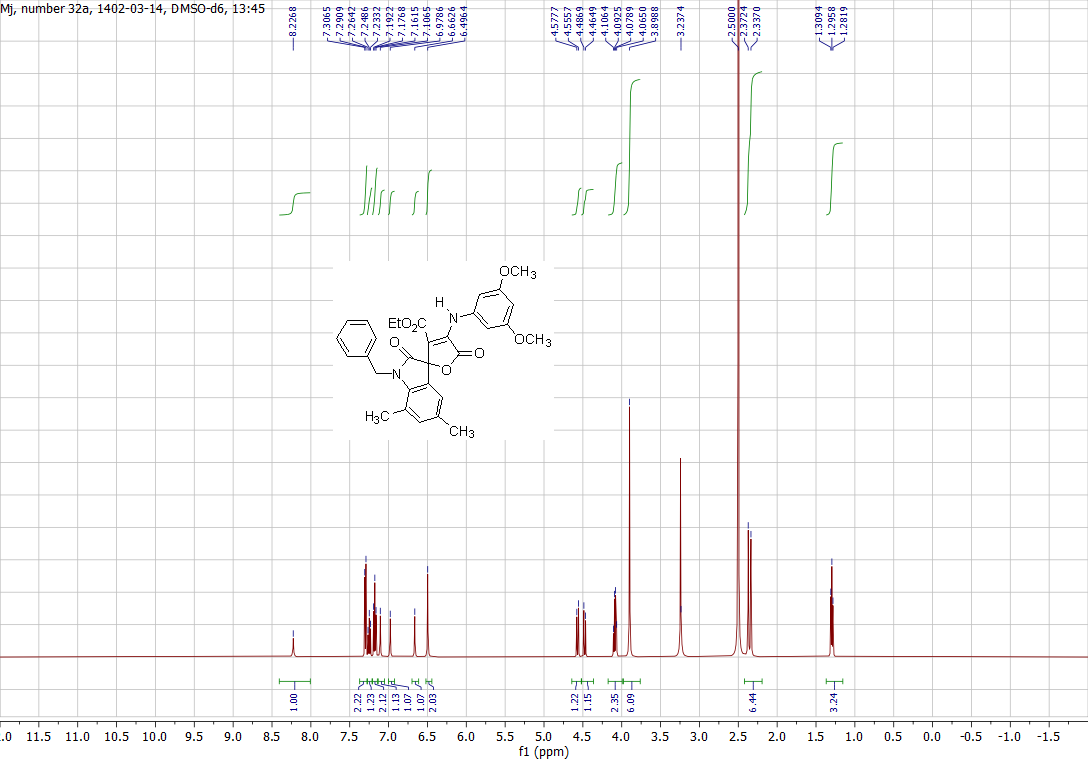


**Figure S_62_:** ^1^H-NMR spectrum of **31a**


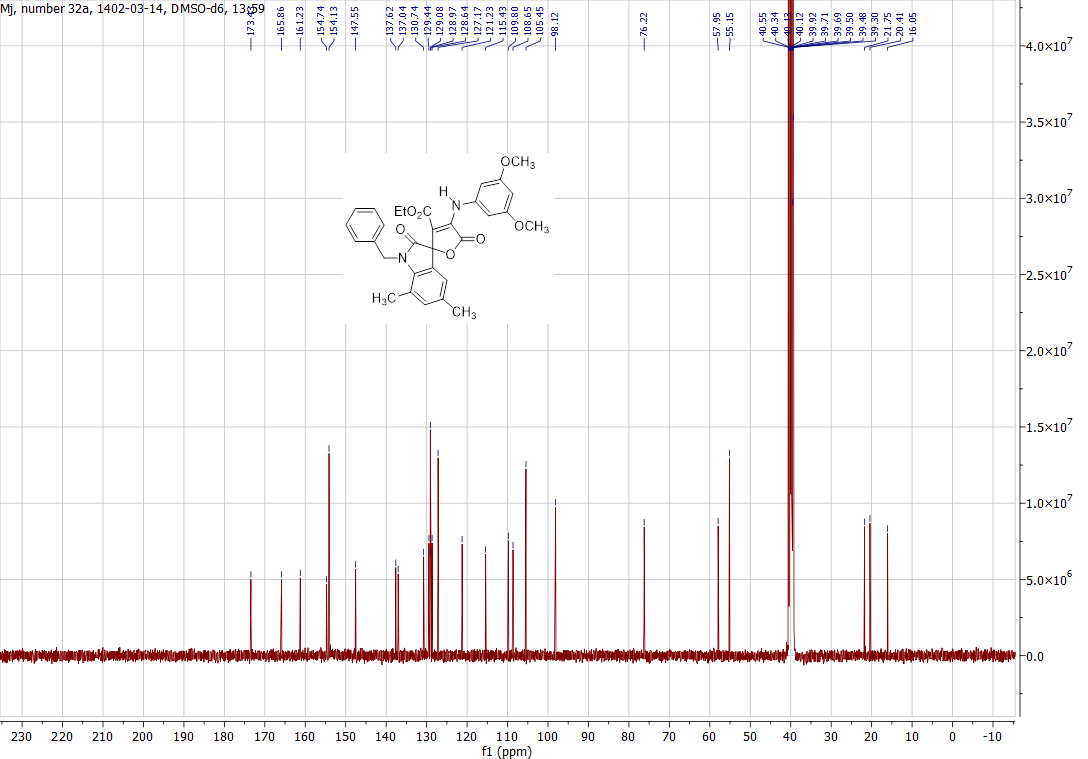


**Figure S_63_:** ^13^C-NMR spectrum of **31a**


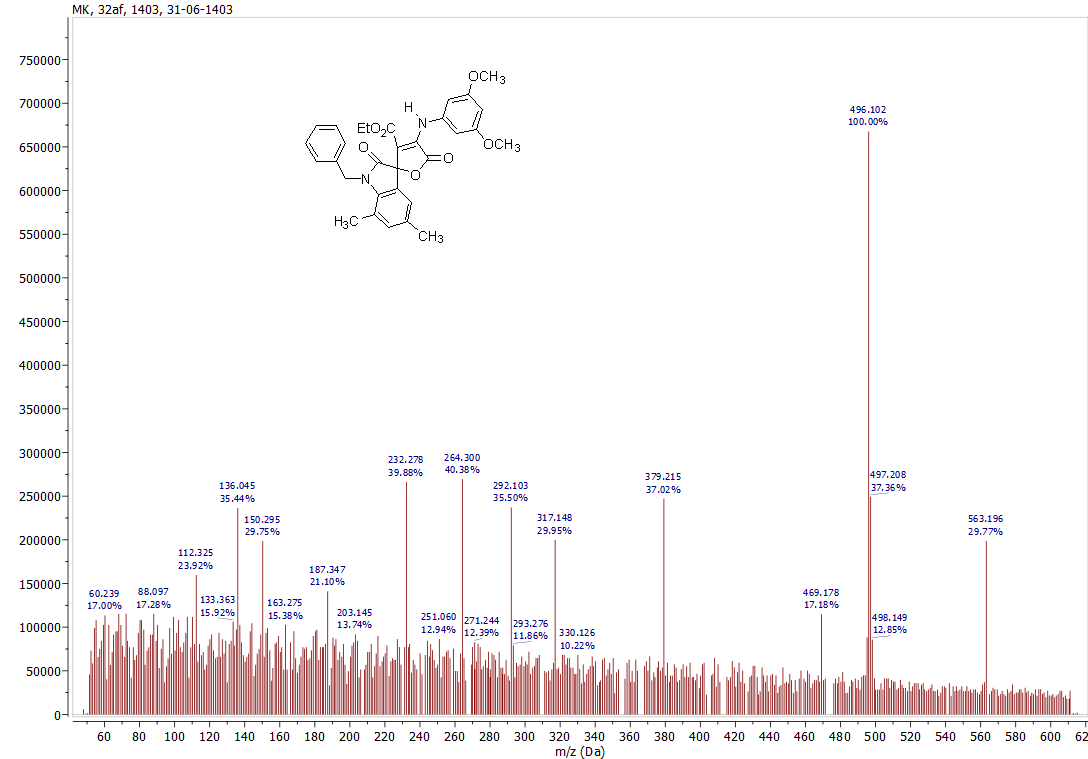


**Figure S_63_:** MASS spectrum of **31a**

1. *Corresponding author.

   *E-mail address:* khalaj_mehdi@yahoo.com (M. Khalaj) and molakhatami@gmail.com (S. M. Khatami) [↑](#footnote-ref-1)
